# SOX2 induces LPCAT1 expression to promote cholesterol metabolic reprogramming-mediated invasion and metastasis in osteosarcoma

**DOI:** 10.3389/fmolb.2025.1679244

**Published:** 2025-11-21

**Authors:** Linchao Zhu, Ying Sun

**Affiliations:** 1 Department of Pediatric Surgery, Henan Provincial People’s Hospital, Zhengzhou, Henan, China; 2 Department of Clinical Laboratory, Third People’s Hospital of Henan Province, Zhengzhou, Henan, China

**Keywords:** osteosarcoma, SOX2, LPCAT1, cholesterol metabolism, metastasis

## Abstract

**Background:**

SOX2 and LPCAT1 are implicated in tumor progression, but their roles in osteosarcoma pathogenesis and cholesterol metabolism remain unclear.

**Method:**

SOX2 and LPCAT1 expression in osteosarcoma tissues and cell lines was assessed via qRT-PCR and Western blot. Functional assays (CCK-8, wound healing and Transwell) evaluated proliferation, migration, and invasion of osteosarcoma cells. SOX2-LPCAT1 binding was confirmed by dual-luciferase reporter assay and ChIP assays. RNA sequencing and bioinformatics analyses explored cholesterol metabolism pathways. *In vitro* and *in vivo* models (xenograft tumor model and lung metastasis model) validated mechanistic roles.

**Result:**

SOX2 and LPCAT1 were overexpressed in osteosarcoma. LPCAT1 or SOX2 overexpression promoted malignant behaviors and cholesterol metabolism (free cholesterol/total cholesterol levels, SREBP1/INSIG1 expression) of osteosarcoma cells, while shSOX2 or shLPCAT1 did the opposite. SOX2 transcriptionally activated LPCAT1. LPCAT1 reversed shSOX2-induced suppression, while LPCAT1 knockdown attenuated SOX2-driven oncogenicity. *In vivo*, LPCAT1 enhanced tumor growth, lung metastasis, and cholesterol metabolism, while these effects were counteracted by SOX2 inhibition.

**Conclusion:**

The SOX2/LPCAT1 axis drives osteosarcoma progression by modulating cholesterol metabolism.

## 1 Introduction

Osteosarcoma is the most common primary malignant bone tumor, predominantly affecting children and adolescents, with a second incidence peak in older adults (Cole et al., 2022). Despite advancements in multimodal therapies—including surgery, chemotherapy, and radiotherapy—the 5-year survival rate for patients with metastatic or recurrent disease remains below 30% (Jiang et al., 2022; Beird et al., 2022). The aggressive nature of osteosarcoma, coupled with its propensity for lung metastasis and chemoresistance, underscores the urgent need to elucidate novel molecular drivers and therapeutic targets (Beird et al., 2022). Recent studies have highlighted metabolic reprogramming, particularly cholesterol dysregulation, as a critical factor in osteosarcoma progression, offering potential avenues for intervention (Xu et al., 2022; Yin et al., 2025).

Cholesterol metabolism plays a pivotal role in cancer cell proliferation, membrane integrity, and signal transduction (King et al., 2022). Elevated cholesterol levels are associated with tumor aggressiveness, metastasis, and therapy resistance in multiple cancers (Xiao et al., 2023; Liu et al., 2023). In osteosarcoma, dysregulated cholesterol biosynthesis has been linked to enhanced survival and migration (Pasello et al., 2022), with key enzymes such as sterol regulatory element-binding protein 1 (SREBP1) contributing to oncogenic phenotypes (Huang X. et al., 2024). However, the upstream regulators of cholesterol metabolism in osteosarcoma remain poorly understood. Identifying these molecular switches could provide new strategies to disrupt osteosarcoma progression by targeting metabolic vulnerabilities.

Lysophosphatidylcholine acyltransferase 1 (LPCAT1), an enzyme critical for phospholipid remodeling, has emerged as a regulator of lipid metabolism and cancer progression (Wang and Tontonoz, 2019). LPCAT1 overexpression is reported in hepatocellular carcinoma, lung cancer, and breast cancer, where it promotes proliferation, metastasis, and chemoresistance (Li et al., 2024; Jiang J. et al., 2024; Korbecki et al., 2024). Mechanistically, LPCAT1 modulates phosphatidylcholine composition, especially for the accumulation of polyunsaturated fatty acids (Korbecki et al., 2024). In osteosarcoma, the functional role and regulatory mechanisms of LPCAT1 remain unexplored. Given the reliance of osteosarcoma on lipid metabolism, investigating LPCAT1’s contribution could uncover novel therapeutic targets.

SOX2, a core transcription factor in pluripotency and stemness, is frequently overexpressed in cancers, including osteosarcoma, where it sustains tumor initiation, metastasis, and therapy resistance (Huang P. et al., 2024). Recent studies implicate SOX2 in metabolic reprogramming, particularly in lipid and glucose metabolism (de Wet et al., 2022). Intriguingly, bioinformatic analysis predicts SOX2-binding sites in the LPCAT1 promoter, suggesting a direct regulatory relationship. While SOX2’s role in osteosarcoma is established (Maurizi et al., 2018), its impact on cholesterol metabolism via LPCAT1 remains unknown. Elucidating this axis could reveal how transcriptional and metabolic networks converge to fuel osteosarcoma progression.

Therefore, we hypothesize that the SOX2/LPCAT1 axis drives osteosarcoma progression by reprogramming cholesterol metabolism, thereby enhancing malignancy and metastatic potential.

## 2 Materials and methods

### 2.1 Tissue samples

This study was approved by the Institutional Review Board of Henan Provincial People’s Hospital. From January 2022 to January 2024, 20 paired osteosarcoma and adjacent non-tumor tissue specimens were collected from Henan Provincial People’s Hospital. Written informed consent was obtained from all participants prior to sample collection.

### 2.2 Cell culture

**FIGURE 1:**
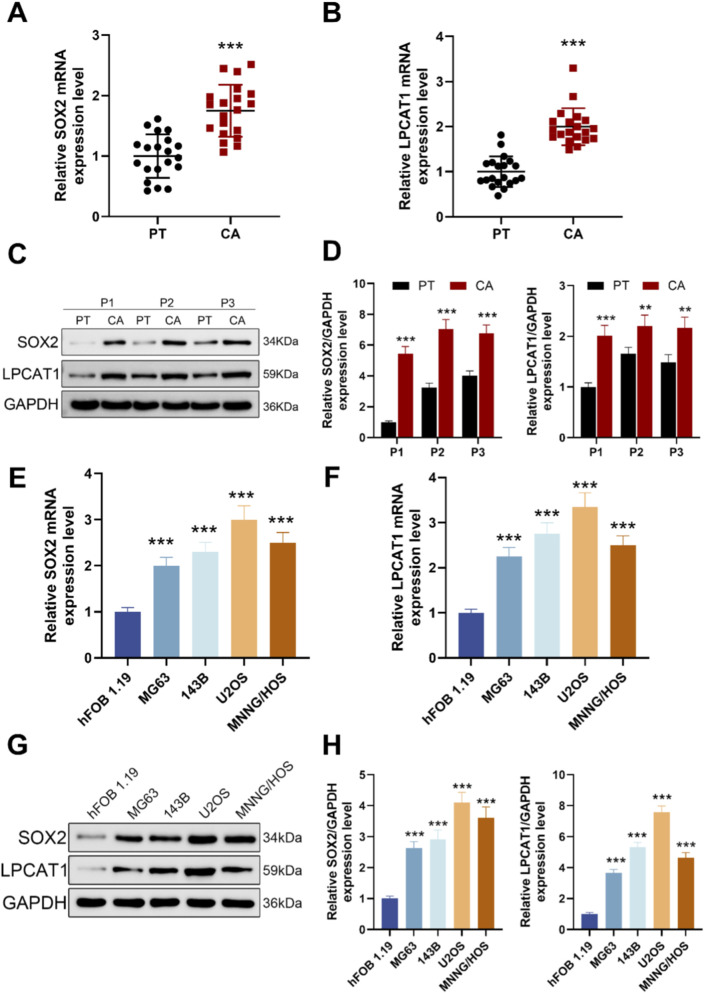
Expression levels of SOX2 and LPCAT1 in osteosarcoma. **(A,B)** qRT-PCR analysis of SOX2 and LPCAT1 expression in tumor tissues (n = 20) and adjacent normal tissues (n = 20) from osteosarcoma patients. **(C,D)** Western blot analysis of SOX2 and LPCAT1 protein levels in osteosarcoma tissues and paired adjacent normal tissues. **(E–H)** qRT-PCR and Western blot detection of SOX2 and LPCAT1 expression in normal osteoblast cell line hFOB1.19 and osteosarcoma cell lines (MG63, 143B, U2OS, and MNNG/HOS). Data are presented as mean ± SD. ^**^ *P* < 0.01, ^***^ *P* < 0.001 vs. PT or hFOB1.19.

Human osteosarcoma cell lines 143B (SNL-192), MG-63 (SNL-229), U-2 OS (SNL-054), and MNNG/HOS (SNL-372) and the human osteoblastic cell line hFOB 1.19 (SNL-217) were obtained from SUNNCELL (China) and cultured in RPMI-1640 complete medium (SNM-001E) at 37 °C in a humidified 5% CO_2_ incubator. Based on baseline LPCAT1 expression across our osteosarcoma panel (Figures 1E,F), we used MG63 (relatively lower endogenous LPCAT1) for gain-of-function (overexpression) studies and U2OS (relatively higher endogenous LPCAT1) for loss-of-function (shRNA knockdown) studies. This pairing minimizes non-physiologic ceiling/floor effects of perturbation and increases generalizability by evaluating LPCAT1 dependence in two distinct osteosarcoma genetic backgrounds.

### 2.3 Cell transfection

Osteosarcoma cells were transfected using a lipo8000 reagent (C0533, Beyotime, China). The full-length cDNA sequences of LPCAT1 (RefSeq NM_024830.5; NP_079110.3) and SOX2 (RefSeq NM_003106.4; NP_003097.1) were cloned into the pcDNA3.1 vector (VT1001, Youbio, China) to generate overexpression plasmids (oe-LPCAT1 and oe-SOX2), with the empty vector serving as a negative control (NC). Short hairpin RNAs (shRNAs) targeting LPCAT1 (shLPCAT1, sc-91777-SH, target sequence: 5′-GGAACTCTGATCCAGTATATA-3′) and SOX2 (shSOX2, sc-38408-SH, target sequence: 5′-AGGAGCACCCGGATTATAAAT-3′) were purchased from Santa Cruz (United States). Twenty-four hours prior to transfection, 5 × 10^5^ cells were seeded into 6-well plates. For each well, the transfection mixture was prepared by combining transfection reagent, plasmid, and serum-free medium, followed by incubation with cells for 48 h. Transfection efficiency was assessed via quantitative reverse transcription-polymerase chain reaction (qRT-PCR) and Western blot (WB).

### 2.4 qRT-PCR

Total RNA was extracted from cells or tissues using the Total RNA Extraction Kit (AG21024, Agbio, China). cDNA was synthesized using the cDNA Synthesis Kit (AG11615, Agbio), followed by quantitative PCR with SYBR Green qPCR MasterMix (AG11762, Agbio) on an ABI7900-HT-Fast system (Applied Biosystems, United States). Gene expression was normalized to glyceraldehyde-3-phosphate dehydrogenase (GAPDH) and analyzed using the 2^−ΔΔCT^ method. Primer sequences are listed in Table 1.

**TABLE 1:** Primers used in this study.

Genes	Forward primer 5′–3′	Reverse primer 5′–3′	Product size (bp)
SOX2	AACCAGCGCATGGACAGTTA	GACTTGACCACCGAACCCAT	278
LPCAT1	CATGAGGCTGCGGGGATG	TTCCCCAGATCGGGATGTCT	494
INSIG1	CTCTCGGCCAGGAAGCG	AGGCGGAGGAAAAGATGGTG	417
SREBP1	TTCCGAGGAACTTTTCGCCG	GGGAGGGCTTCCTGTAGAGA	700
Vimentin	CTCTGGCACGTCTTGACCTT	TTGCGCTCCTGAAAAACTGC	871
N-cadherin	TCAGGCGTCTGTAGAGGCTT	ATGCACATCCTTCGATAAGACTG	94
GAPDH	GATTCCACCCATGGCAAATTC	CTGGAAGATGGTGATGGGATT	87

### 2.5 Western blot

Cells or tissues were lysed in 0.5 mL of RIPA buffer (G3424, GBCBIO, China). Protein concentrations were quantified using the BCA Protein Assay Kit (G3522, GBCBIO). Proteins were separated by 10% SDS-PAGE (G3402, GBCBIO) and transferred onto PVDF membranes (36125ES03, Yeasen, China). After blocking with 5% BSA (0332, GBCBIO), membranes were incubated overnight at 4 °C with primary antibodies (Table 2), followed by HRP-conjugated secondary antibodies for 1 h at room temperature. Protein bands were visualized using ECL reagent (G3308, GBCBIO) and imaged on an iBright FL1500 system (A44115, Invitrogen, United States). GAPDH served as the loading control. Band intensities were quantified using ImageJ (version 1.8.0, NIH, Bethesda, MD, United States).

**TABLE 2:** Antibodies used in this study.

Name	Catalog	Dilution	Species	Manufacturer
SOX2	AF5140	1/1000	Rabbit	Affinity
SOX2 (ChIP)	CST #23064	1/50	Rabbit	Cell signaling Tech, USA
LPCAT1	DF12033	1/1000	Rabbit	Affinity
INSIG1	DF12412	1/1000	Rabbit	Affinity
SREBP1	AF6283	1/1000	Rabbit	Affinity
Vimentin	AF7013	1/1000	Rabbit	Affinity
N-cadherin	AF5239	1/1000	Rabbit	Affinity
GAPDH	ab8245	1/2000	Mouse	Abcam
Goat anti rabbit	ab205718	1/10000	Goat	Abcam
Goat anti mouse	ab205719	1/10000	Goat	Abcam

### 2.6 Cell counting kit 8 (CCK-8)

Cells (3 × 10^3^/well) were seeded in 96-well plates and cultured for 0–72 h. Cell viability was assessed using the CCK-8 kit (R22305, Shyuanye, China) by measuring absorbance at 490 nm after 2 h of incubation.

### 2.7 Wound healing assay

Cells (5 × 10^5^/well) were seeded in 6-well plates. A scratch was made using a sterile pipette tip, and migration was monitored in serum-free medium. Images were captured at 0 h and 24 h using an optical microscope (Nikon, Japan). Migration distance was quantified with ImageJ.

### 2.8 Transwell assay

Transwell inserts (Millipore, United States) were pre-coated with Matrigel (1 mg/mL; 356234, BD Biosciences, United States). Cells (1 × 10^5^) in serum-free medium were seeded into the upper chamber, while the lower chamber contained 10% FBS as a chemoattractant. After 24 h, invaded cells were fixed, stained with 0.1% crystal violet (G9507, GBCBIO), and counted under a microscope.

### 2.9 Dual-luciferase reporter assay

Potential SOX2-binding sites in the LPCAT1 promoter were predicted using JASPAR (https://jaspar.elixir.no/). A 2.2 kb promoter fragment of human LPCAT1 (−2000 to +200 bp relative to the transcription start site [TSS] of RefSeq transcript NM_024830.5, GRCh38) was amplified and cloned into the pGL3.0-Basic vector (VT1554, Youbio, China). The corresponding hg38 genomic span is chr5: 1,523,760–1,525,960. Within this region, a SOX2 motif was identified at −1961 to −1971 bp upstream of the TSS, with the wild-type (WT) sequence 5′-TTTCAATGGAA-3’. A mutant (MUT) reporter was generated by replacing the core motif “CAAT” with “GCCT” (mutant sequence 5′-TTTGCCTGGAA-3′), abolishing SOX2 binding. For reporter assays, osteosarcoma cells (1 × 10^5^/well) were co-transfected with WT or MUT promoter-luciferase constructs, the Renilla control plasmid pRL-TK (VT1568, Youbio, China), and oe-SOX2 or negative control (NC) using Lipo8000 (C0533, Beyotime, China). Luciferase activity was measured 48 h post-transfection using the Dual-Luciferase Reporter Assay Kit (11402ES60, Yeasen, China). Firefly luciferase activity was normalized to Renilla luciferase activity.

### 2.10 Chromatin immunoprecipitation (ChIP)

ChIP was performed using a ChIP assay kit (NR3302M, Newonebio, China). Briefly, cells (1 × 10^6^) were crosslinked with 1% formaldehyde, lysed, and sonicated to obtain 200–500 bp chromatin fragments. Lysates were immunoprecipitated overnight with anti-SOX2 (23064, Cell signaling technology, United States) or IgG, followed by protein A/G agarose beads. After washing, DNA was eluted and analyzed by qPCR.

### 2.11 RNA sequencing and analysis

RNA-seq was performed in MG63 for oe-LPCAT1 vs. NC (lower endogenous baseline) and in U2OS for shLPCAT1 vs. shNC (higher endogenous baseline) to maintain perturbations within a physiologic dynamic range and to reduce cell-line-specific bias. Total RNA from MG63 (oe-LPCAT1/NC) and U2OS (shLPCAT1/shNC) cells were extracted for RNA-seq (Illumina NovaSeq 6000). Differentially expressed genes (DEGs) were identified using the limma package (|log2FC| > 1, adj. *P* < 0.05). Functional enrichment was analyzed via Gene Ontology (GO) and Reactome pathways (*P* < 0.05).

### 2.12 Cholesterol quantification

Free cholesterol (FC, E-BC-K004-M) and total cholesterol (TC, E-BC-K109-M) levels in cells (5 × 10^6^) or tissues (50 mg) were measured using commercial kits (Elabscience, China).

### 2.13 Animals

All animal procedures were approved by the Institutional Animal Care and Use Committee of Henan Provincial People’s Hospital. BALB/c nude mice (5–6 weeks old; Vital River, China) were subcutaneously injected with MG63 cells (1 × 10^7^) transfected with oe-LPCAT1 and/or shSOX2 (n = 6/group) as previously described (Fu et al., 2024). Tumor volume was measured every 3 days. Mice were anesthetized with isoflurane inhalation (2%–3% for induction, 1%–2% for maintenance) during all surgical and invasive procedures to minimize pain and distress. At day 28, mice were humanely euthanized by intraperitoneal injection of an overdose of pentobarbital sodium (>150 mg/kg), and tumors were excised and weighed. For lung metastasis, cells (2 × 10^6^) were injected via the tail vein as previously described (Fu et al., 2024). After 8 weeks, mice were euthanized using the same method (overdose pentobarbital sodium), and metastatic nodules were counted.

### 2.14 Statistical analysis

Statistical analysis was experimented by GraphPad Prism 8.0. The measurement data were expressed by mean ± standard deviation. The independent sample t-test was used for the comparison between groups. One-way or two-way ANOVA was used for multiple group comparisons. Figures 1A,B was analyzed by Paired t-test. Differences with *P* < 0.05 were considered statistically significant.

## 3 Results

### 3.1 SOX2 and LPCAT1 were highly expressed in osteosarcoma tissues and cells

Scatter plots of 20 matched patient pairs showed higher SOX2 and LPCAT1 mRNA in osteosarcoma tissues and adjacent normal tissues (Figures 1A,B). Immunoblotting of three representative pairs confirmed increased SOX2 and LPCAT1 protein in tumor relative to matched normal tissue, with GAPDH as loading control (Figures 1C,D). Across cell models, qRT-PCR showed higher SOX2 and LPCAT1 transcripts in osteosarcoma lines (MG-63, 143B, U2OS, MNNG/HOS) than in the human osteoblastic line hFOB 1.19 (Figures 1E,F). Western blotting further corroborated these findings at protein level in these cell models (Figure G, H).

### 3.2 LPCAT1 overexpression or knockdown modulated the biological behavior of osteosarcoma cells

**FIGURE 2:**
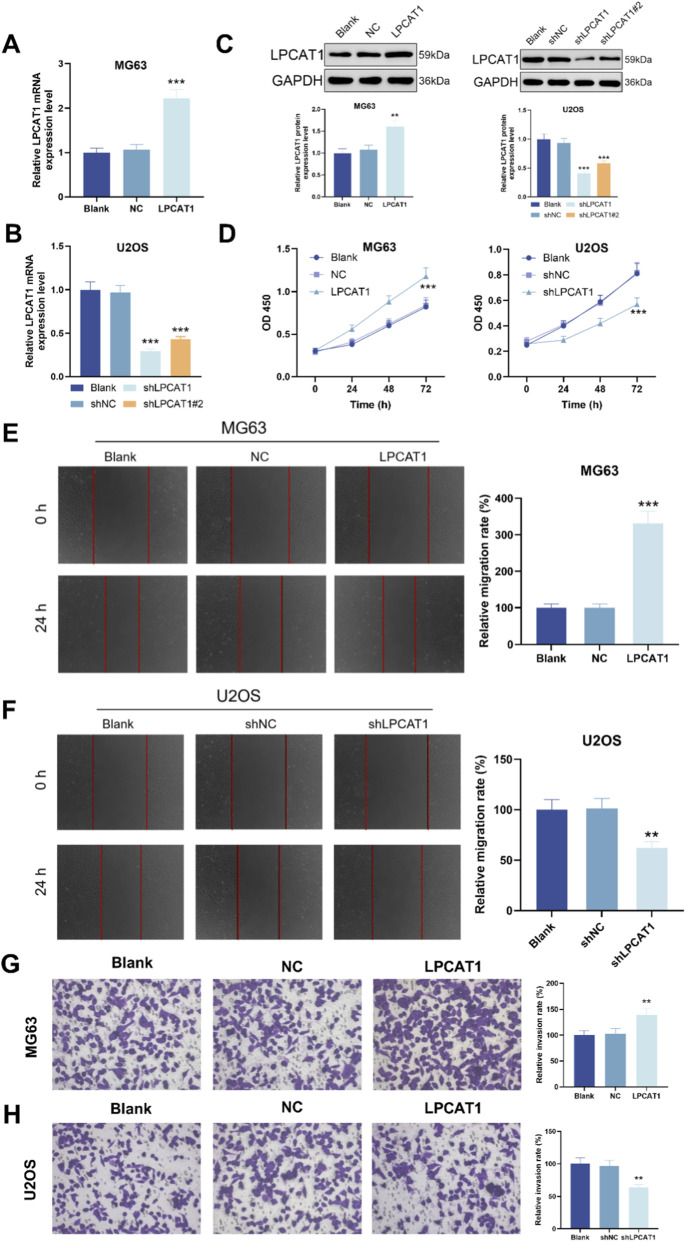
LPCAT1 promotes osteosarcoma of LPCAT1 on osteosarcoma cell viability, migration and invasion. **(A–C)** qRT-PCR and Western blot verification of transfection efficiency for LPCAT1 overexpression plasmid and shLPCAT1. For **(D–H)**, MG63 cells were transfected with NC or LPCAT1 overexpression plasmid, while U2OS cells were transfected with shNC or shLPCAT1. **(C,D)** Cell viability assessed by CCK-8 assay. **(E,F)** Cell migration ability was evaluated by wound healing assay. **(G,H)** Cell invasion capacity was determined by Transwell assay. Scale bars: 200 μm. Data are representative of three independent experiments (mean ± SD). ^**^ *P* < 0.01, ^***^ *P* < 0.001 vs. NC or shNC.

To investigate the functional role of LPCAT1 in osteosarcoma, we overexpressed LPCAT1 in MG63 cells and silenced it in U2OS cells with two independent shRNAs. qRT–PCR and Western blot verified the intervention efficiency of LPCAT1 (Figures 2A–C). LPCAT1 overexpression significantly enhanced cell viability, migration, and invasion, whereas LPCAT1 knockdown exerted the opposite effects (Figures 2C–H).

### 3.3 SOX2 mediated LPCAT1 transcription

**FIGURE 3:**
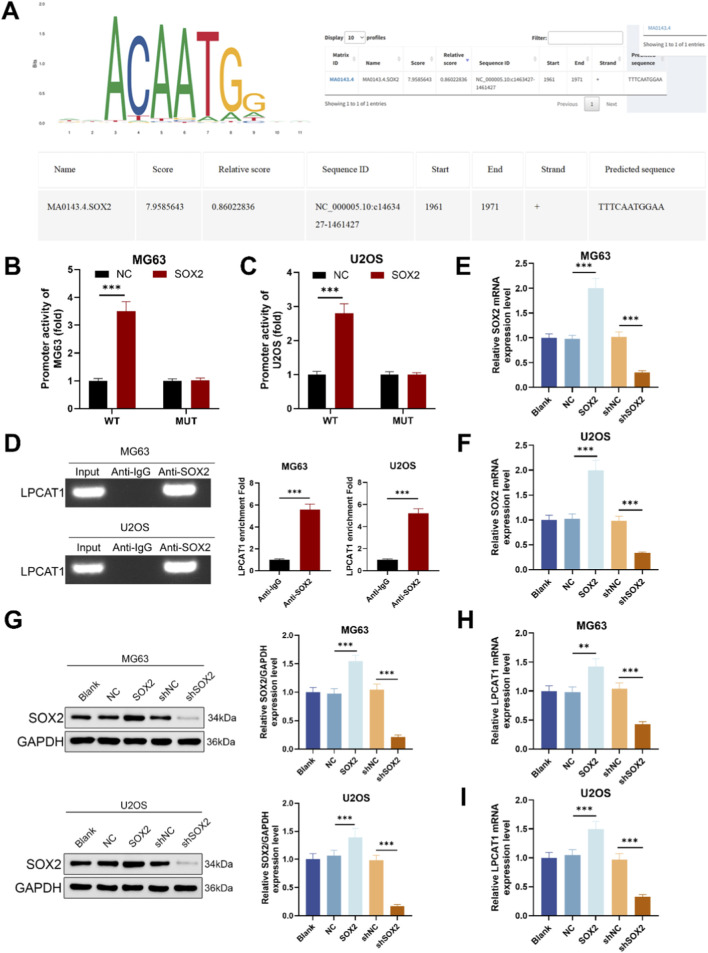
SOX2 mediated transcriptional regulation of LPCAT1. **(A)** JASPAR prediction of SOX2 binding sites in the LPCAT1 promoter region. **(B,C)** Dual-luciferase reporter assay validating the interaction between SOX2 and LPCAT1 promoter. **(D)** ChIP assay demonstrating the binding of SOX2 to LPCAT1 promoter region. **(E,F)** qRT-PCR confirmation of SOX2 overexpression and knockdown efficiency. **(G–I)** Western blot and qRT-PCR analysis of LPCAT1 expression following SOX2 modulation. Data are representative of three independent experiments (mean ± SD). ^**^ *P* < 0.01, ^***^ *P* < 0.001 vs. NC, anti-IgG or shNC.

Bioinformatic analysis using JASPAR predicted a potential SOX2-binding site in the promoter region of LPCAT1 (Figure 3A). Reporter assays with a motif-mutant construct showed reduced activity upon motif disruption (Figures 3B,C). ChIP–qPCR using anti-SOX2 demonstrated significant enrichment of LPCAT1 promoter amplicons compared with IgG in MG63 and U2OS (Figure 3D), validated the direct binding of SOX2 to the LPCAT1 promoter. To assess the regulatory role of SOX2, we transfected MG63 and U2OS cells with SOX2 overexpression plasmid or shSOX2, verifying transfection efficiency (Figures 3E,F). qRT-PCR and Western blot analyses revealed that SOX2 overexpression upregulated LPCAT1 expression, while SOX2 knockdown suppressed it (Figures 3G,H).

### 3.4 Bioinformatic analysis of LPCAT1’s role in cholesterol metabolism in osteosarcoma

**FIGURE 4:**
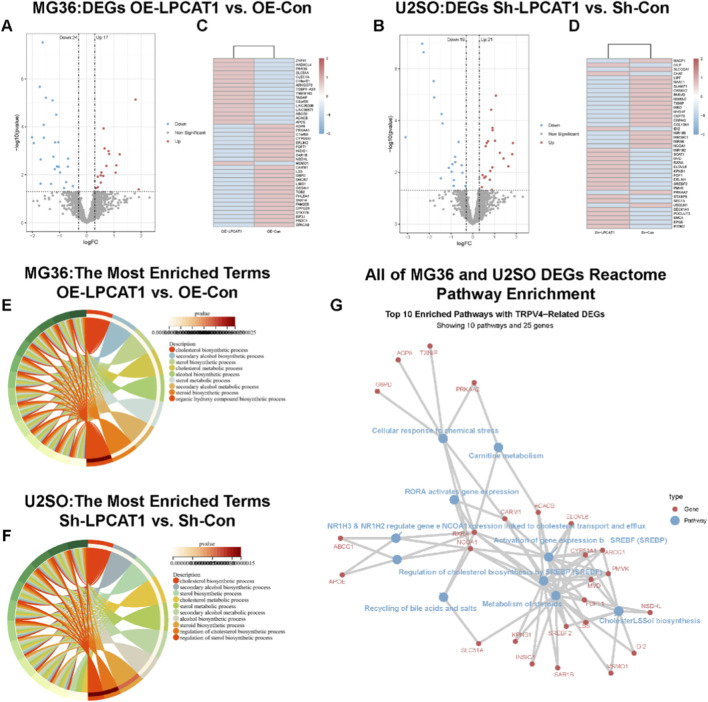
RNA sequencing transcriptomic analysis. **(A,B)** Volcano plot and heatmap showing DEGs in MG63 cells after LPCAT1 overexpression. **(C,D)** Volcano plot and heatmap displaying DEGs in U2OS cells following LPCAT1 knockdown. **(E,F)** GO enrichment analysis revealing LPCAT1-regulated cholesterol biosynthesis processes in both MG63 and U2OS cells. **(G)** Reactome pathway analysis visualizing LPCAT1-affected genes in osteosarcoma cells.

RNA sequencing was performed on MG63 cells transfected with NC/LPCAT1 and U2OS cells transfected with shNC/shLPCAT1, with DEGs illustrated in Figures 4A–D. GO enrichment analysis indicated that LPCAT1 significantly influenced cholesterol biosynthesis in both cell lines (Figures 4E,F). Further pathway analysis using the Reactome database revealed that LPCAT1 overexpression or silencing altered cholesterol biosynthesis pathways (Figure 4G).

### 3.5 *In vitro* validation of LPCAT1’s role in cholesterol metabolism in osteosarcoma cells

**FIGURE 5:**
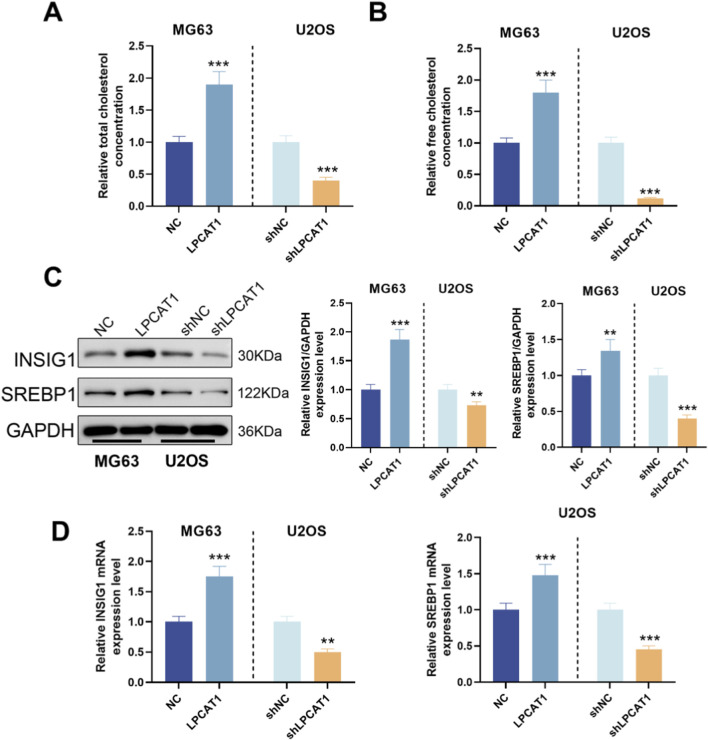
Role of LPCAT1 in cholesterol metabolic reprogramming of osteosarcoma cells. **(A,B)** Cholesterol content measurement in osteosarcoma cells after LPCAT1 modulation using commercial assay kits. **(C,D)** Western blot and qRT-PCR analysis of cholesterol metabolism-related proteins following LPCAT1 overexpression or knockdown. Data are representative of three independent experiments (mean ± SD). ^**^ *P* < 0.01, ^***^ *P* < 0.001 vs. NC.

We measured TC and FC levels in osteosarcoma cells following LPCAT1 modulation. LPCAT1 overexpression increased TC and FC levels, whereas LPCAT1 knockdown reduced them (Figures 5A,B). Additionally, LPCAT1 upregulated the expression of cholesterol metabolism-related proteins (INSIG1 and SREBP1), while its silencing had the opposite effect (Figures 5C,D). Intervention efficiency for these assays was confirmed by RT–qPCR and Western blot (Supplementary Figure S1).

### 3.6 SOX2/LPCAT1 axis regulates osteosarcoma cell behavior and cholesterol metabolism

**FIGURE 6:**
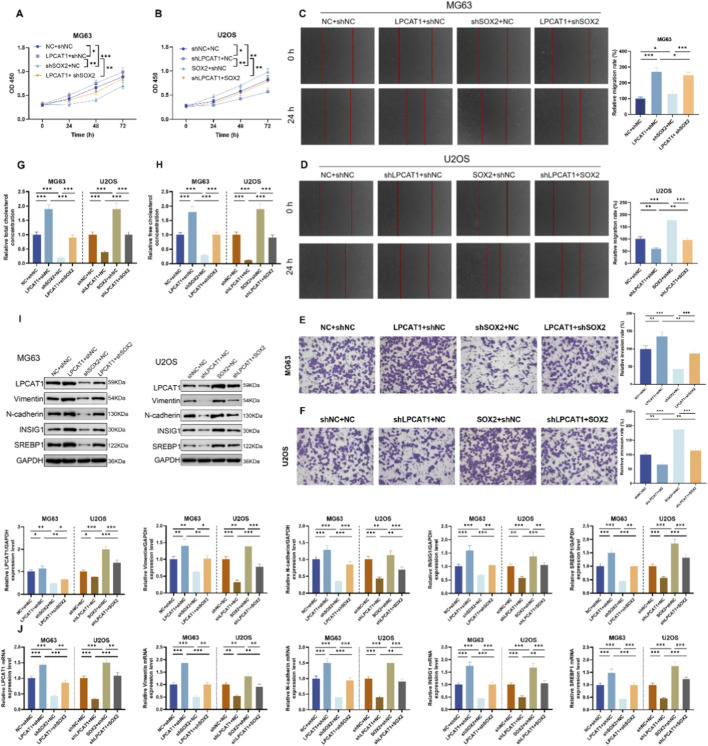
*In vitro* validation of SOX2/LPCAT1 axis in regulating osteosarcoma cell biological behaviors and cholesterol metabolism. MG63 cells were co-transfected with oe-LPCAT1 and/or shSOX2, while U2OS cells were co-transfected with shLPCAT1 and/or SOX2. **(A,B)** Cell viability was determined by CCK-8 assay. **(C,D)** Migration ability was assessed by wound healing assay. **(E,F)** Invasion capacity was evaluated by Transwell assay. **(G,H)** Intracellular cholesterol level measurement. **(I,J)** Western blot and qRT-PCR analysis of metastasis- and cholesterol metabolism-related proteins. Data are representative of three independent experiments (mean ± SD). ^*^ *P* < 0.05, ^**^ *P* < 0.01, ^***^ *P* < 0.001 vs. NC+shNC, shLPCAT1+NC or LPCAT1+shNC, SOX2+shNC or shSOX2+NC.

Epistasis assays were performed in MG63 and U2OS, and intervention efficiency was confirmed by RT–qPCR and Western blot (Supplementary Figures S2A,B). In MG63 cells, shSOX2 suppressed cell viability (Figures 6A,B), migration (Figures 6C,D), and invasion (Figures 6E,F), reduced TC and FC levels (Figures 6G,H), as well as downregulated LPCAT1, metastasis-related proteins (Vimentin and N-cadherin), and cholesterol metabolism-related proteins (Figures 6I,J). These effects were rescued by LPCAT1 overexpression (Figures 6A–J). Conversely, in U2OS cells, SOX2 overexpression enhanced above-mentioned malignant phenotypes and cholesterol metabolism, which were attenuated by LPCAT1 knockdown (Figures 6A–J).

### 3.7 *In vivo* validation of SOX2/LPCAT1 in osteosarcoma growth, metastasis, and cholesterol metabolism

**FIGURE 7:**
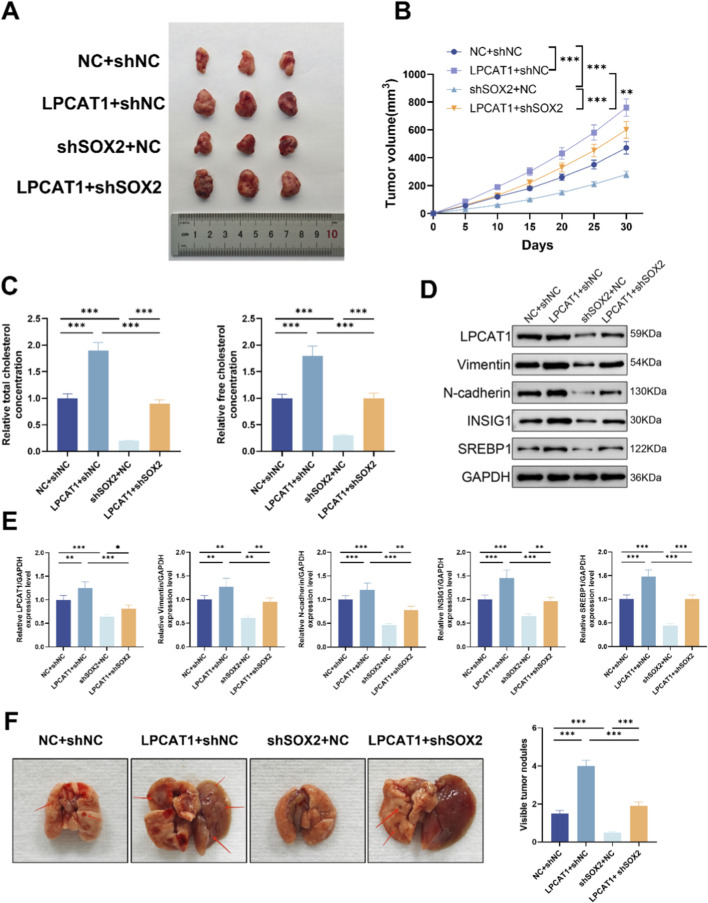
*In vivo* validation of SOX2/LPCAT1 axis in osteosarcoma progression and cholesterol metabolism. For **(A–D)**, MG63 cells were transfected with oe-LPCAT1 and/or shSOX2, and then subcutaneously injected into nude mice to construct a xenograft model. **(A)** Representative images of xenograft tumors. **(B)** Tumor volume. **(C)** Cholesterol content measurement in tumor tissues. **(D,E)** Western blot analysis of metastasis- and cholesterol metabolism-related proteins in xenograft tumors. **(F)** MG63 cells transfected with oe-LPCAT1 and/or shSOX2 were injected into the tail vein of nude mice to construct a lung metastasis model. The number of pulmonary nodules was counted. Data are representative of three independent experiments (mean ± SD). ^**^ *P* < 0.01, ^***^ *P* < 0.001 vs. NC+shNC, LPCAT1+shNC, and shSOX2+NC.

In a xenograft tumor model, LPCAT1 overexpression increased tumor volume (Figures 7A,B), elevated TC and FC levels (Figure 7C), and upregulated LPCAT1, metastasis-related proteins, and cholesterol metabolism-related proteins (Figures 7D,E), whereas shSOX2 exerted opposing effects (Figures 7A–E). In a lung metastasis model, LPCAT1 increased metastatic nodule formation, while shSOX2 reduced it (Figure 7F). Notably, LPCAT1 overexpression reversed the inhibitory effects of shSOX2 on osteosarcoma growth, metastasis, and cholesterol metabolism. The intervention used in the xenograft tumor model were verified for the intended perturbations by RT–qPCR and Western blot (Supplementary Figure S3).

## 4 Discussion

This study provides compelling evidence establishing the SOX2/LPCAT1/cholesterol metabolism axis as a novel molecular mechanism driving osteosarcoma progression and metastasis. Through integrated analysis of clinical samples, molecular biology experiments, and animal models, we demonstrated that both SOX2 and LPCAT1 are significantly overexpressed in osteosarcoma tissues and cell lines compared to normal controls. Functional studies revealed that this axis plays a dual role in regulating both malignant behaviors and metabolic reprogramming. Specifically, we identified that LPCAT1 serves as a key mediator connecting SOX2 transcriptional regulation with cholesterol biosynthesis, thereby creating a vicious cycle that promotes tumor aggressiveness. Additionally, our *in vivo* experiments provided direct evidence that targeting this axis significantly inhibits both primary tumor growth and pulmonary metastasis, suggesting its therapeutic potential.

The significance of cholesterol metabolism in osteosarcoma pathogenesis has been increasingly recognized but remains incompletely understood. Our current work substantially advances this field by elucidating how aberrant cholesterol biosynthesis contributes to osteosarcoma progression at multiple levels. On one hand, cholesterol levels can modulate lipid raft activity (Zaborowska-Mazurkiewicz et al., 2025), while on the other hand, increased cholesterol content reduces membrane fluidity, which may render tumors more aggressive as characterized by enhanced cell migration and metastatic potential (Yin et al., 2025; Jiang W. et al., 2024). This finding helps explain the frequent lung metastasis observed in osteosarcoma patients, as pulmonary tissue is known to be cholesterol-rich (Dang and Reboldi, 2024).

LPCAT1 emerges from our study as a central player in osteosarcoma biology with multifaceted functions. Beyond its well-characterized role in phospholipid remodeling, we uncovered its previously unrecognized capacity to orchestrate cholesterol metabolism in osteosarcoma cells. Mechanistically, LPCAT1 appears to exert its oncogenic effects through both metabolic and non-metabolic mechanisms. On one hand, it directly modulates the activity of SREBP signaling pathway to enhance cholesterol biosynthesis (Tao et al., 2021). On the other hand, it facilitates epithelial-mesenchymal transition (EMT) by regulating the expression of vimentin and N-cadherin, thereby promoting migratory and invasive capacities of tumor cells (Shen et al., 2022). The striking observation that LPCAT1 overexpression alone could induce lung metastasis in our animal model highlights its critical role in metastatic progression. Clinically, the consistent upregulation of LPCAT1 across different osteosarcoma subtypes suggests its potential as a universal therapeutic target.

Our ChIP and dual-luciferase reporter assays unequivocally demonstrated that SOX2 directly binds to the LPCAT1 promoter to activate its transcription. SOX2 is a critical transcription factor that plays an important role in embryonic development and the maintenance of stem cell pluripotency (Ding et al., 2023). However, its aberrant expression in cancer is closely associated with tumor initiation, progression, and therapy resistance (Grimm et al., 2020). SOX2 can also induce epithelial-mesenchymal transition (EMT) by regulating ZEB1, N-cadherin, and other factors, thereby enhancing tumor cell migration and invasion and promoting metastasis (Li et al., 2023). Notably, elevated SOX2 expression has been reported to promote tumorigenesis and metastasis in osteosarcoma, while SOX2 inhibition can ameliorate these malignant phenotypes (Chen et al., 2023; Wang et al., 2024). However, no studies to date have demonstrated that SOX2 regulates cholesterol metabolism. This study is the first to report that SOX2 indirectly participates in cholesterol metabolism in osteosarcoma cells by transcriptionally regulating LPCAT1. Our data nominate SOX2/LPCAT1 driven cholesterol accumulation as actionable. Prior studies show efficacy of statins, BET/CDK7 inhibitors, and SOAT1 blockade (e.g., avasimibe) in osteosarcoma or related cancer models, with SREBP-axis inhibitors (e.g., fatostatin) providing complementary upstream control. These agents outline immediate, literature-supported strategies to test this pathway therapeutically in future work.

Several limitations of our study should be acknowledged to properly interpret the findings. First, while we established the SOX2-LPCAT1 regulatory relationship *in vitro* and *in vivo*, the clinical correlation between their expression patterns needs validation in larger patient cohorts with long-term follow-up. Second, the precise mechanisms by which cholesterol metabolites influence osteosarcoma cell invasion require further elucidation, particularly regarding their potential roles in modifying the tumor microenvironment. Third, while our luciferase reporter and ChIP-PCR assays provide strong locus-specific evidence for SOX2 binding to the LPCAT1 promoter, we recognize that high-resolution genome-wide analyses such as ATAC-seq or ChIP-seq would offer more comprehensive insight into chromatin accessibility and SOX2 occupancy. These advanced analyses are planned for future work to expand upon our current findings. Finally, while shRNA knockdowns demonstrate functional effects, CRISPR/Cas9 knockout models would offer stronger causal evidence and will be pursued in follow-up studies.

In conclusion, our study advances the understanding of osteosarcoma pathogenesis by identifying the SOX2/LPCAT1/cholesterol metabolism axis as a driver of tumor progression and metastasis. These findings have important translational implications on multiple fronts. Diagnostically, SOX2 and LPCAT1 expression levels may serve as valuable biomarkers for risk stratification. Therapeutically, our work provides strong rationale for developing targeted interventions against this axis, either through direct inhibition of LPCAT1 enzymatic activity or via disruption of SOX2 transcriptional function.

## Data Availability

The datasets generated and analyzed during the current study are available in the Zenodo repository, DOI: 10.5281/zenodo.17588323.
