# Supplementary material for: SOX2 induces LPCAT1 expression to promote cholesterol metabolic reprogramming-mediated invasion and metastasis in osteosarcoma
Source: Front Mol Biosci. 2025 Nov 21;12:1679244. doi: 10.3389/fmolb.2025.1679244 (PMC12678303; doi:10.3389/fmolb.2025.1679244)
Supplement: Supplementary file 1 [file Supplementaryfile1.docx]

**Supplementary Figures**


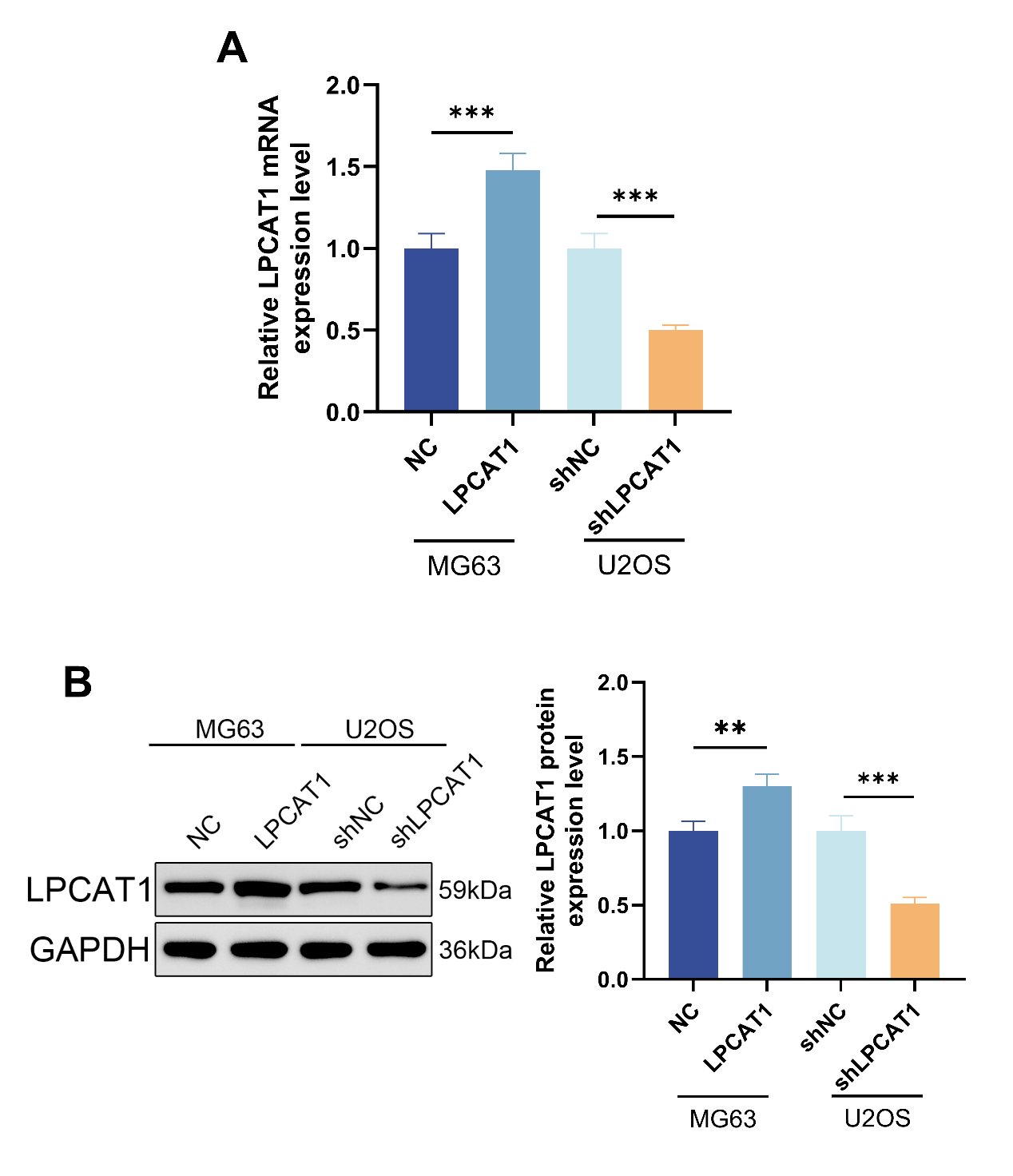


**Supplementary Figure S1.** **Validation of LPCAT1 perturbation efficiency.** (A-B) RT–qPCR and immunoblot confirm efficient LPCAT1 overexpression in MG63 and knockdown in U2OS cells. Data are representative of three independent experiments (mean ± SD). **P < 0.01, ***P < 0.001


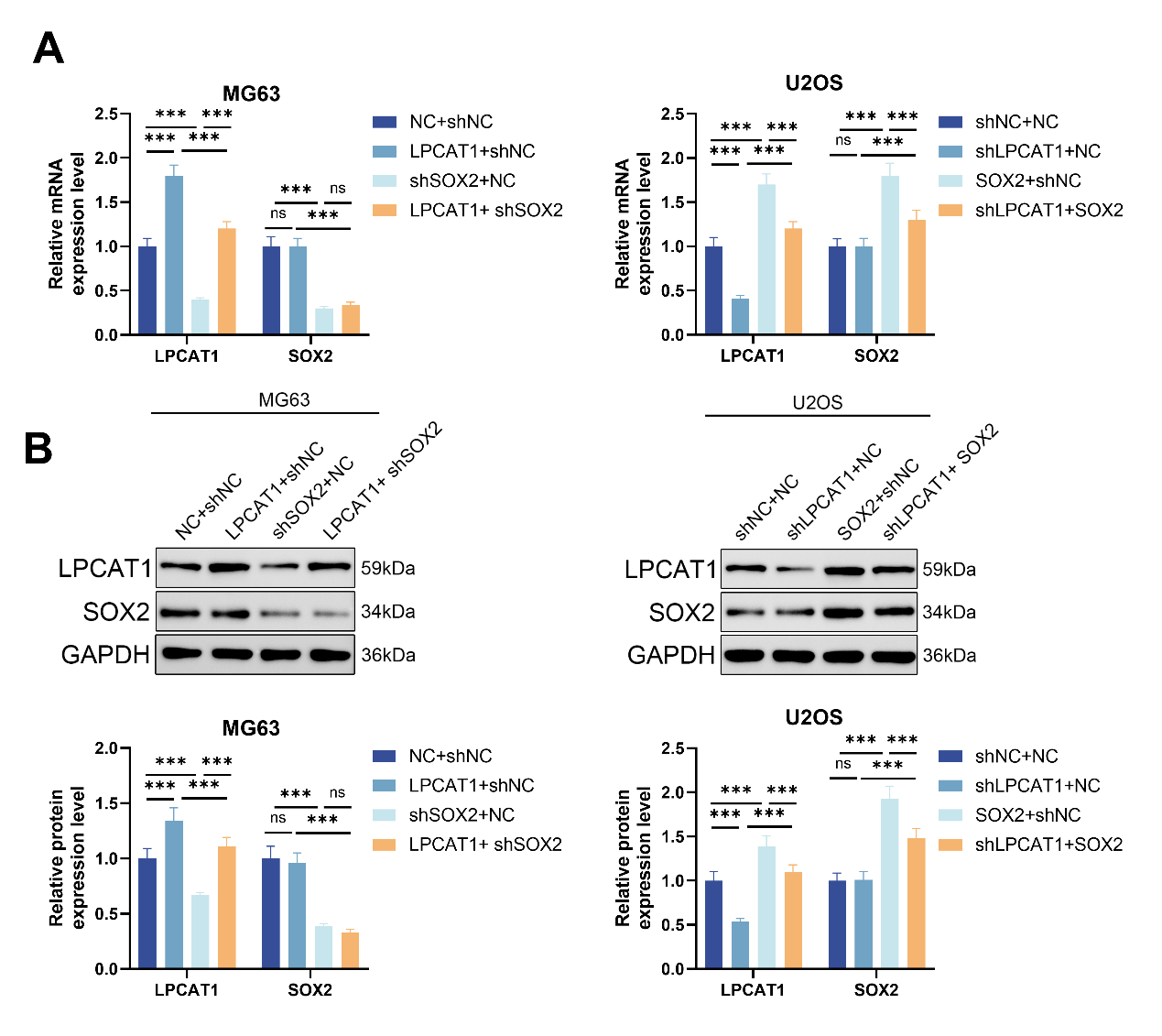


**Supplementary Figure S2**. **Verification of epistasis-intervention efficiency.** (A-B) RT–qPCR and immunoblot confirm co-manipulation of SOX2 and LPCAT1 in MG63 and U2OS cells used for epistasis assays. Data are representative of three independent experiments (mean ± SD). **P < 0.01, ***P < 0.001


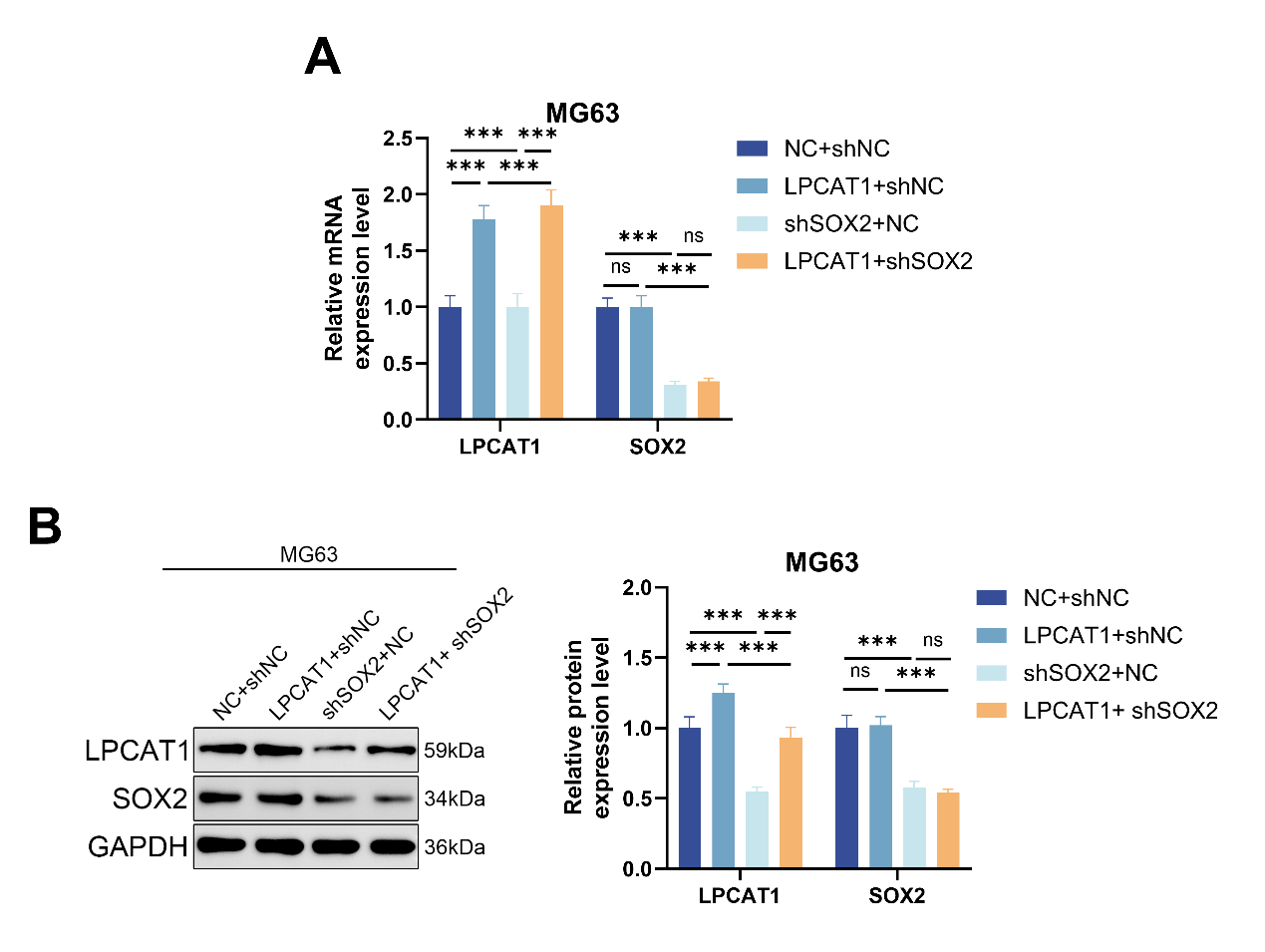


**Supplementary Figure S3. Validation of genetic manipulations in xenograft cohorts.**

RT–qPCR (A) and immunoblot (B) confirm LPCAT1 overexpression and/or SOX2 knockdown in MG63 cells used for tumor implantation. Data are representative of three independent experiments (mean ± SD). **P < 0.01, ***P < 0.001, ns= non-significant.
