# Supplementary figures and images for: SOX2 induces LPCAT1 expression to promote cholesterol metabolic reprogramming-mediated invasion and metastasis in osteosarcoma
Source: Front Mol Biosci. 2025 Nov 21;12:1679244. doi: 10.3389/fmolb.2025.1679244 (PMC12678303; doi:10.3389/fmolb.2025.1679244)

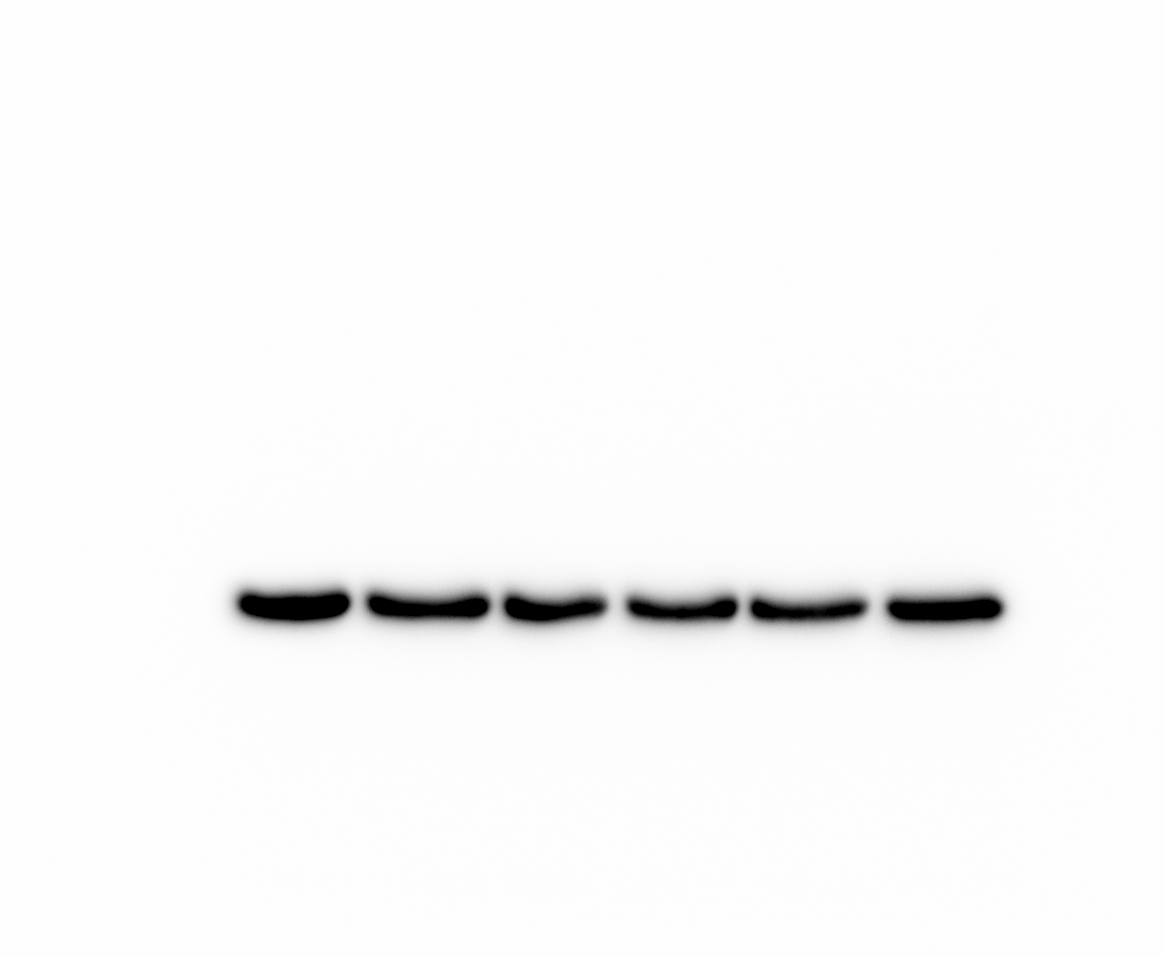

Supplement: Supplementary file 2 [file DataSheet1.zip › Original Western blot images collection/Experiment 1 Chemiluminescence image GAPDH.tif]

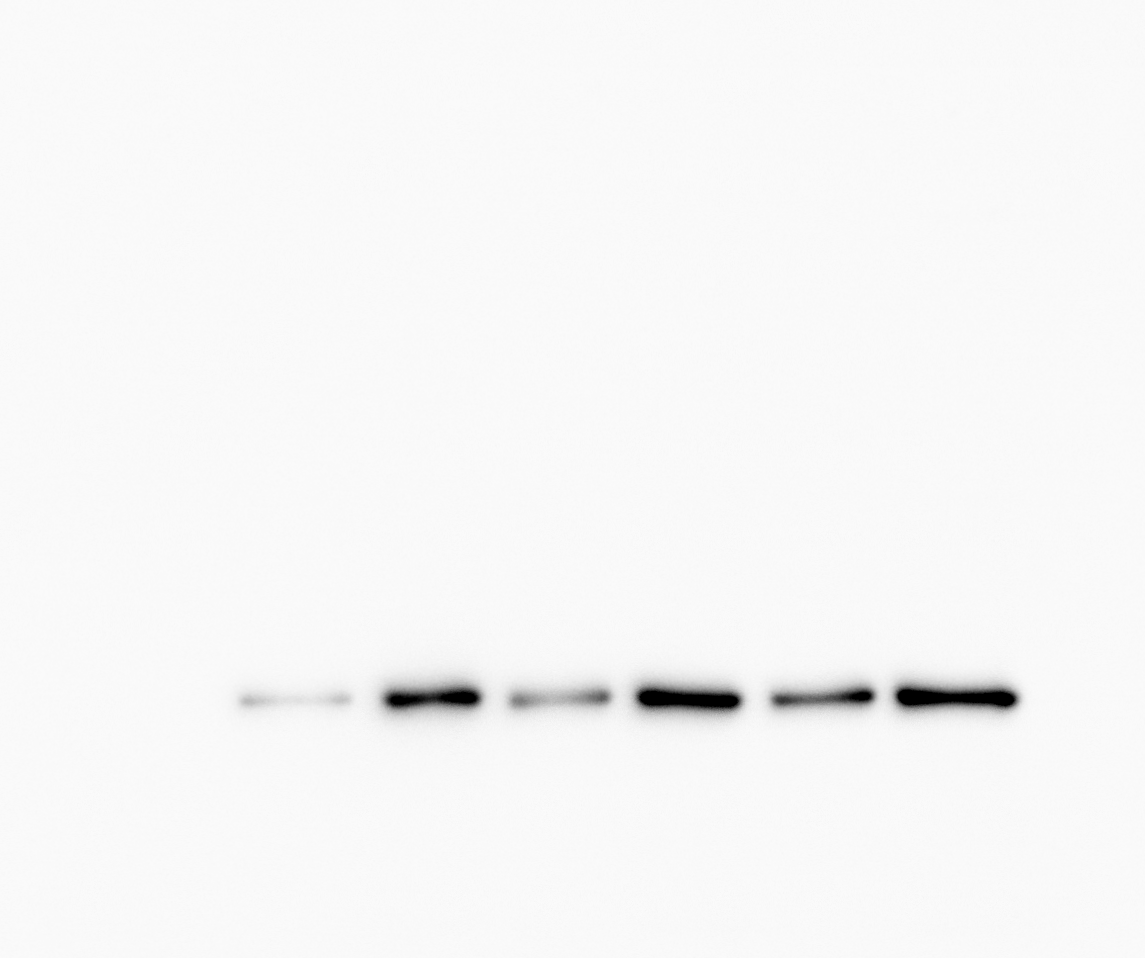

Supplement: Supplementary file 2 [file DataSheet1.zip › Original Western blot images collection/Experiment 1 Chemiluminescence image SOX2.tif]

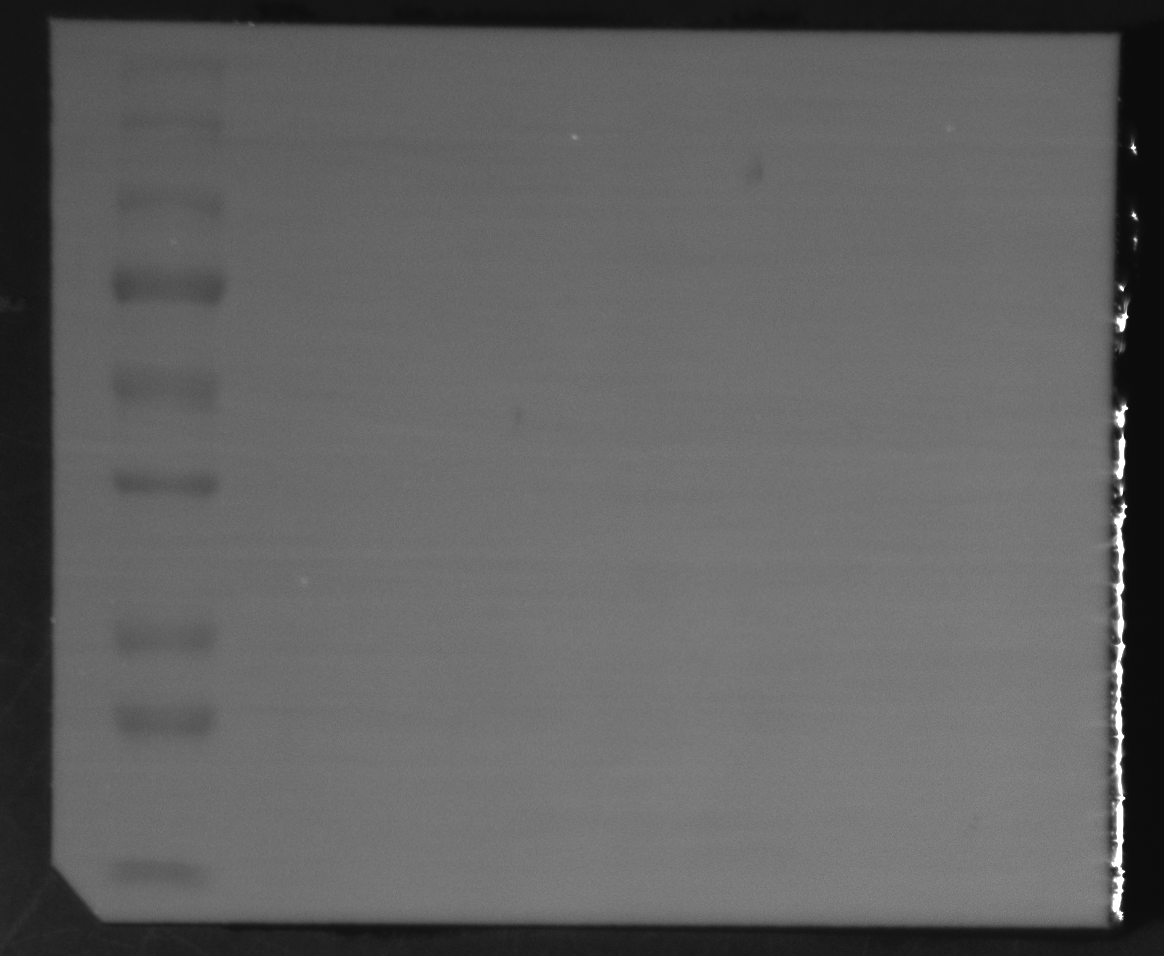

Supplement: Supplementary file 2 [file DataSheet1.zip › Original Western blot images collection/Experiment 1 White light image GAPDH.tif]

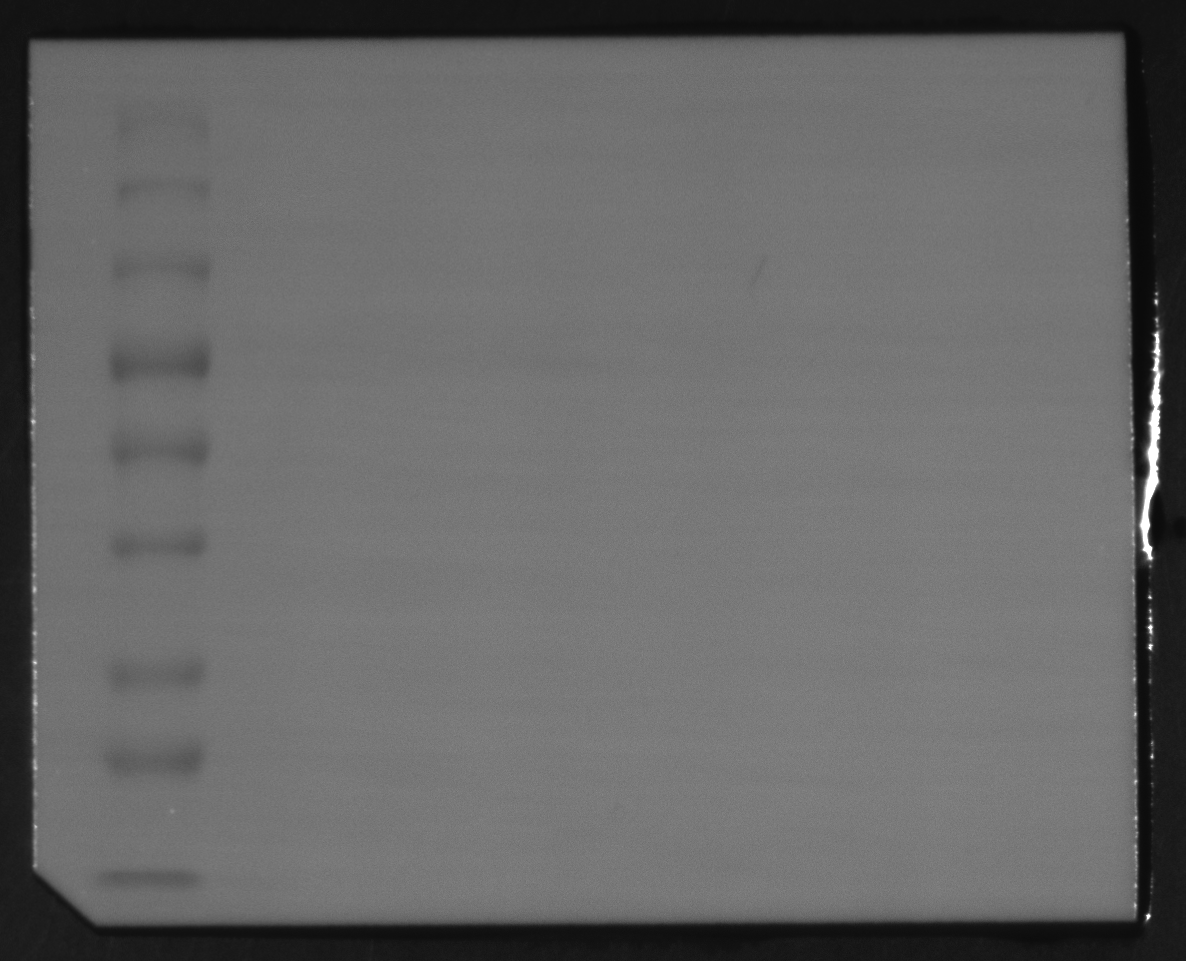

Supplement: Supplementary file 2 [file DataSheet1.zip › Original Western blot images collection/Experiment 1 White light image LPCAT1.tif]

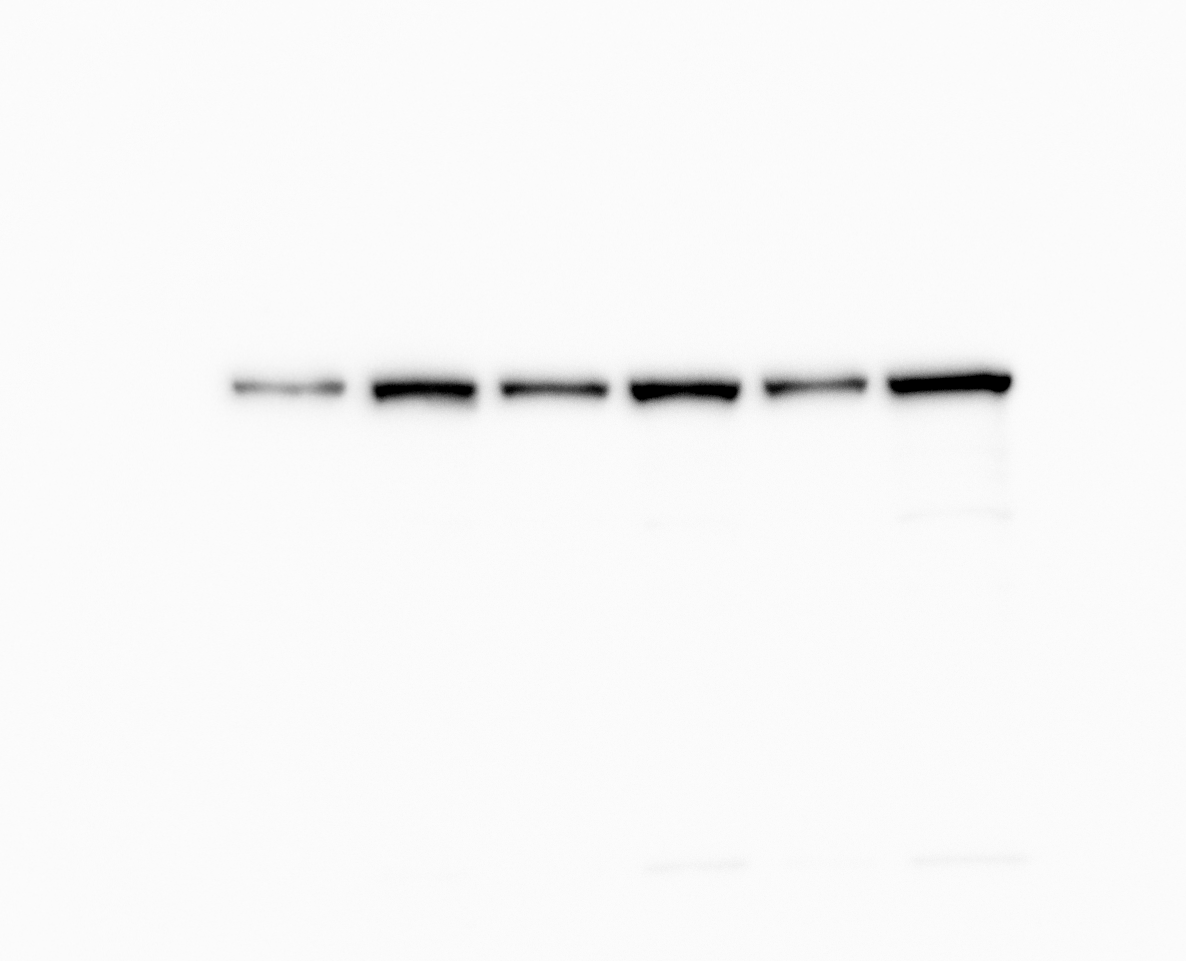

Supplement: Supplementary file 2 [file DataSheet1.zip › Original Western blot images collection/Experiment 1Chemiluminescence image LPCAT1.tif]

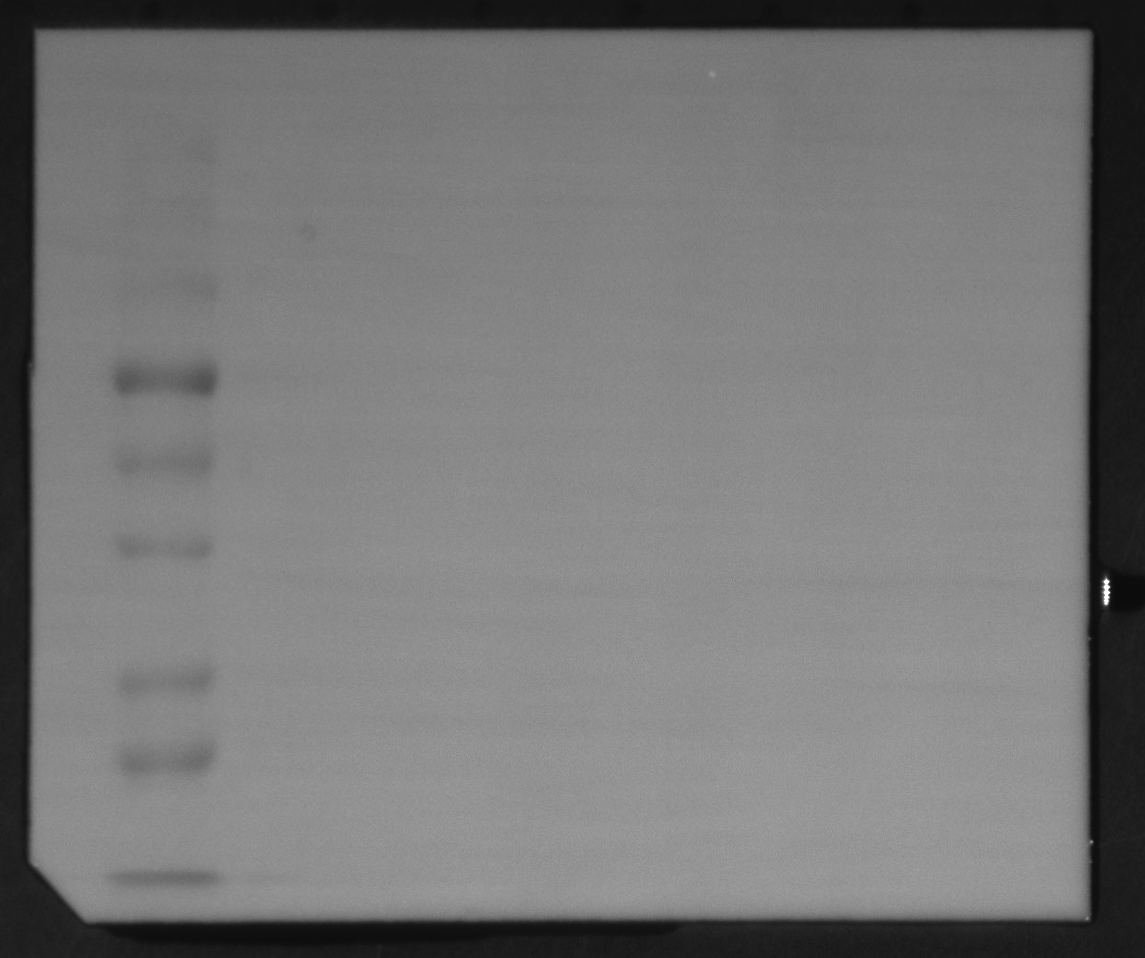

Supplement: Supplementary file 2 [file DataSheet1.zip › Original Western blot images collection/Experiment 1White light image SOX2.tif]

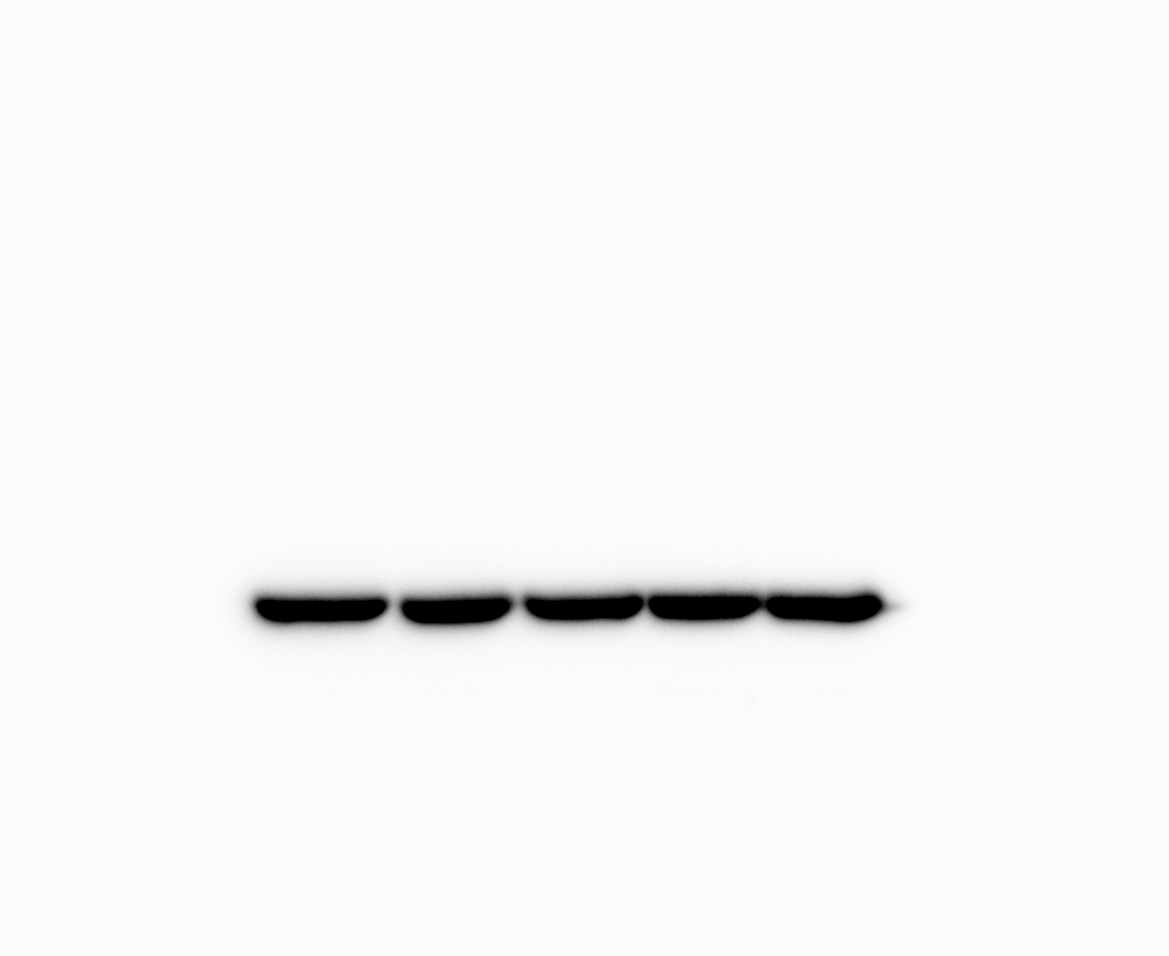

Supplement: Supplementary file 2 [file DataSheet1.zip › Original Western blot images collection/Experiment 2 Chemiluminescence image GAPDH.tif]

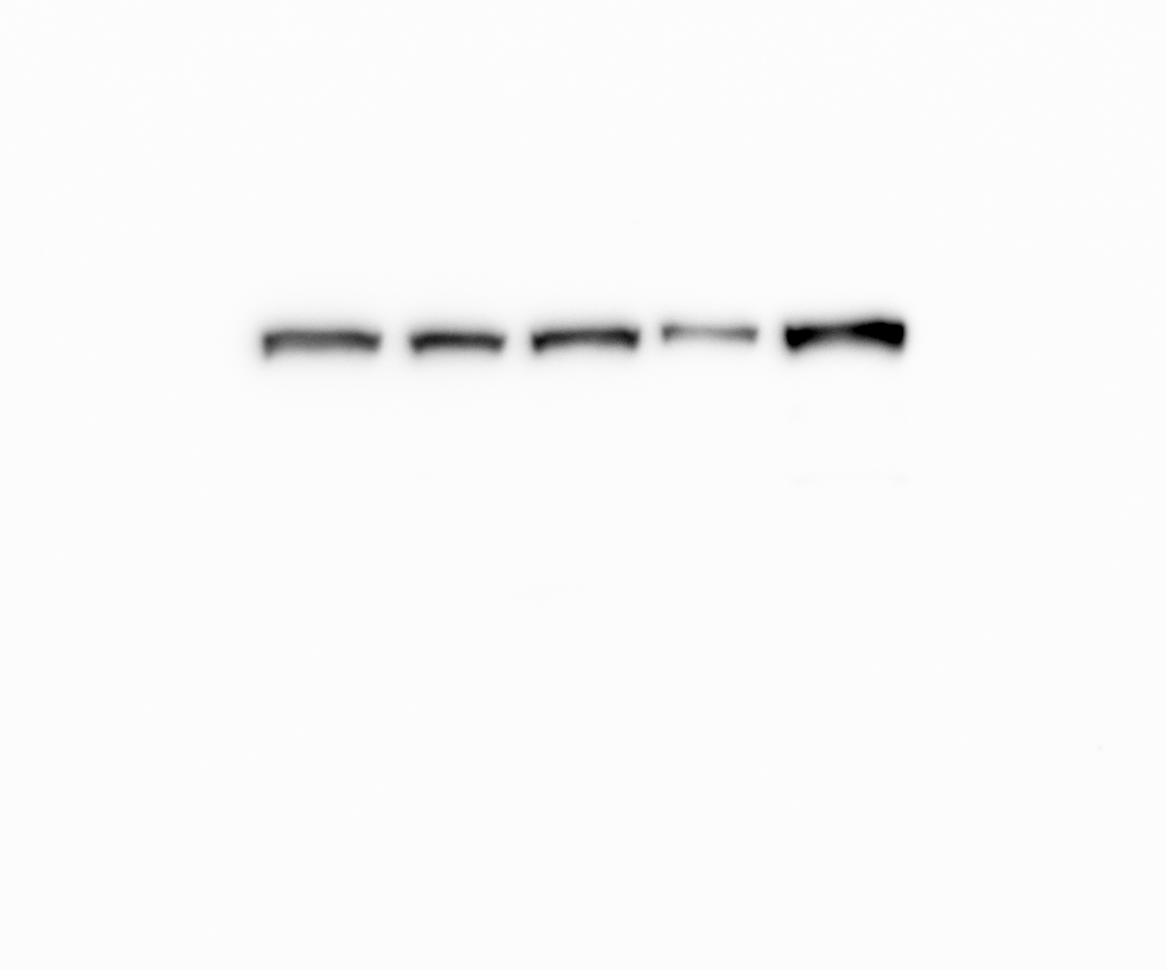

Supplement: Supplementary file 2 [file DataSheet1.zip › Original Western blot images collection/Experiment 2 Chemiluminescence image LPCAT1(MG63).tif]

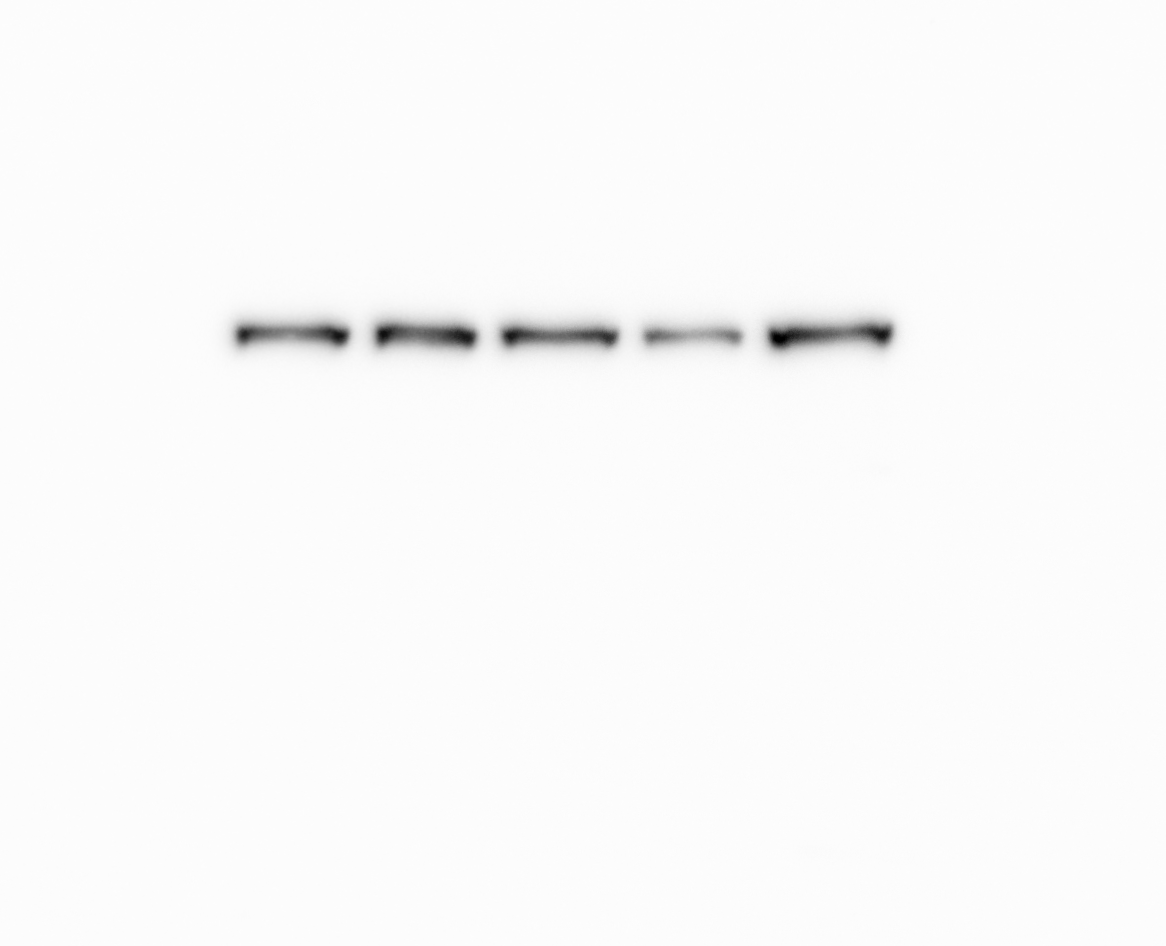

Supplement: Supplementary file 2 [file DataSheet1.zip › Original Western blot images collection/Experiment 2 Chemiluminescence image LPCAT1(U20S).tif]

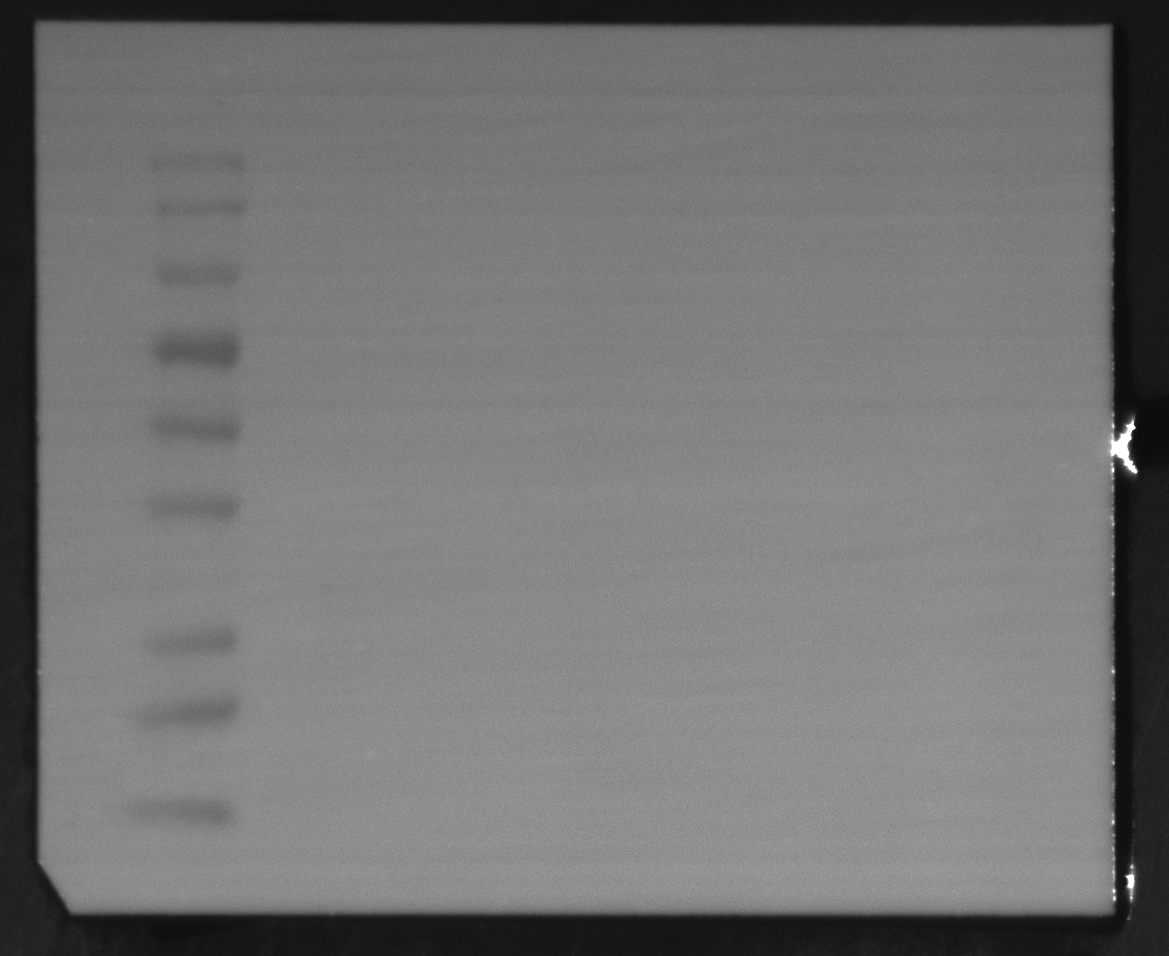

Supplement: Supplementary file 2 [file DataSheet1.zip › Original Western blot images collection/Experiment 2 White light image GAPDH.tif]

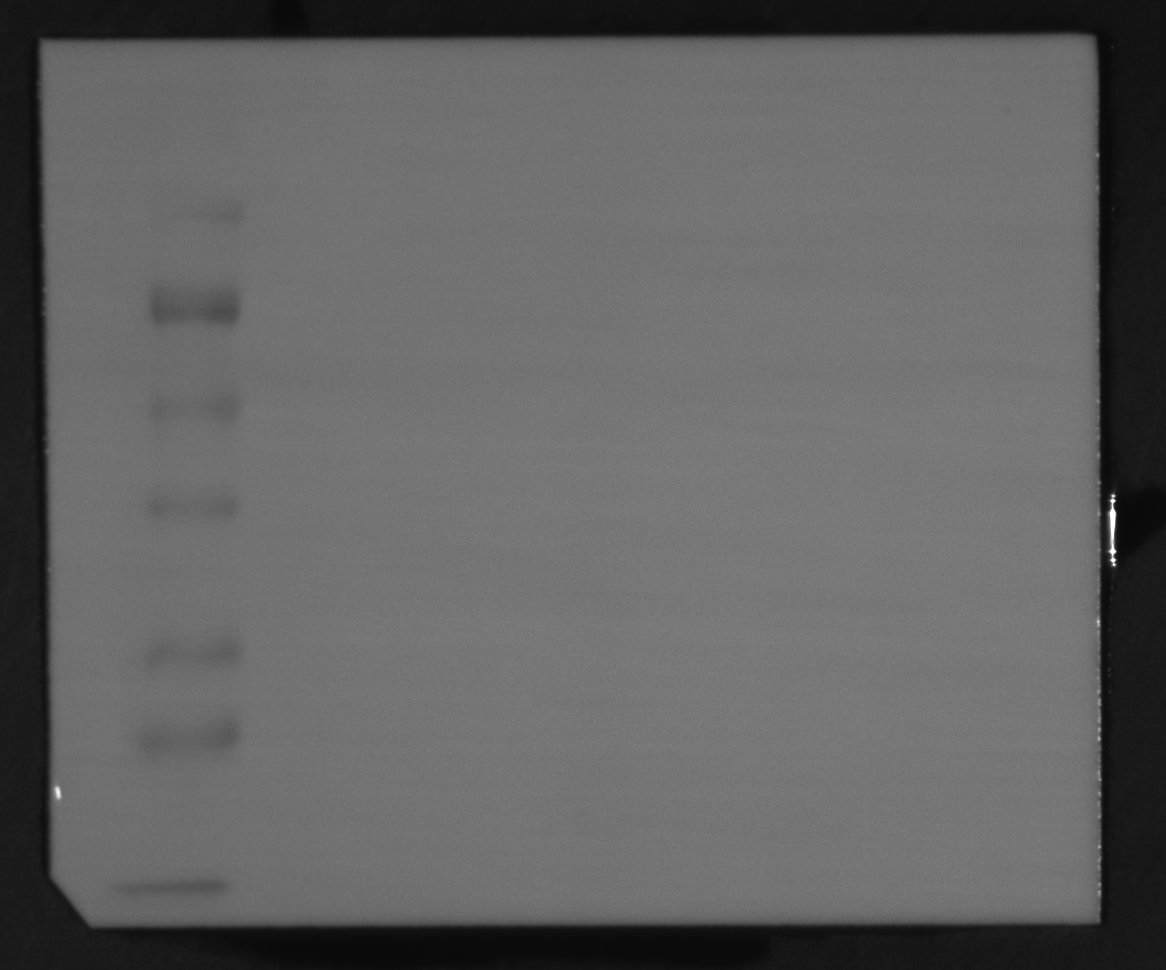

Supplement: Supplementary file 2 [file DataSheet1.zip › Original Western blot images collection/Experiment 2 White light image LPCAT1(MG63).tif]

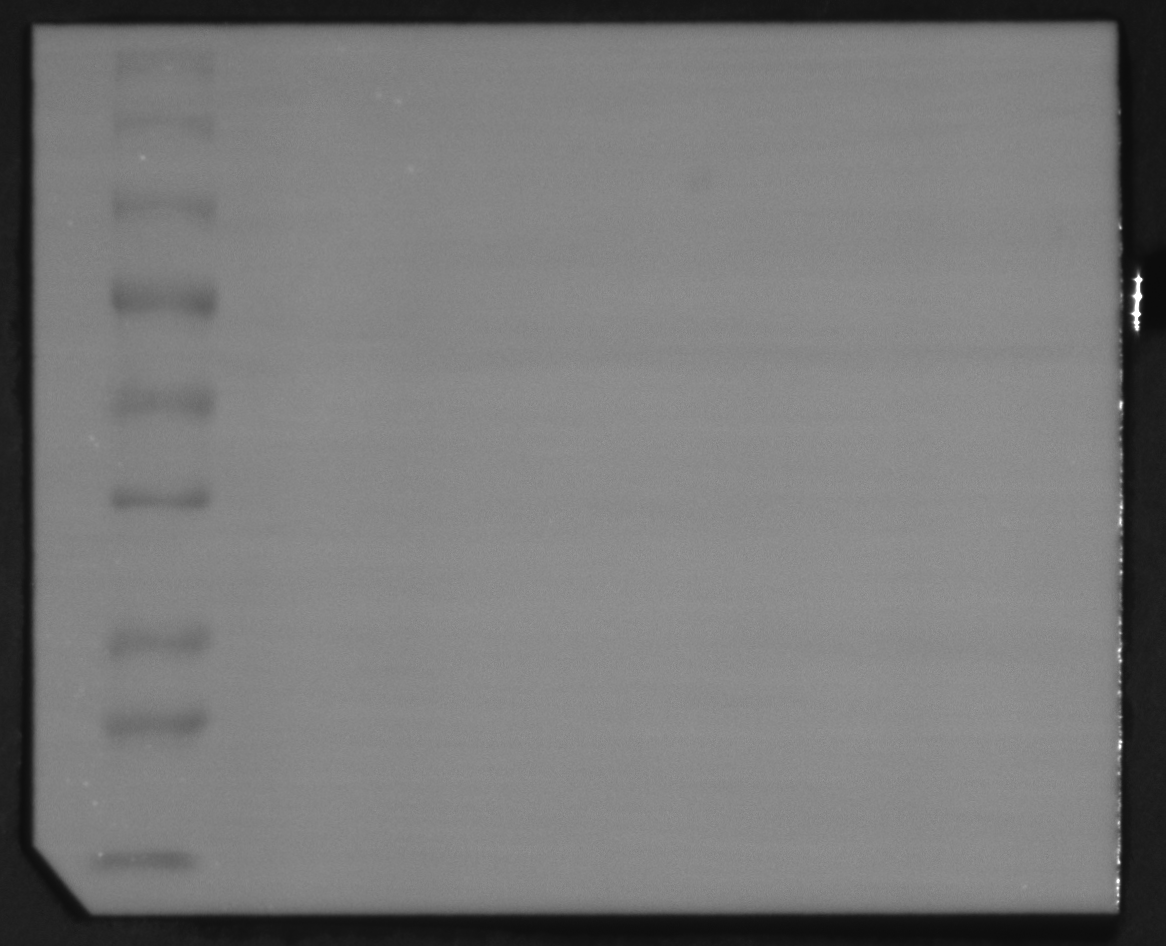

Supplement: Supplementary file 2 [file DataSheet1.zip › Original Western blot images collection/Experiment 2 White light image LPCAT1(U20S).tif]

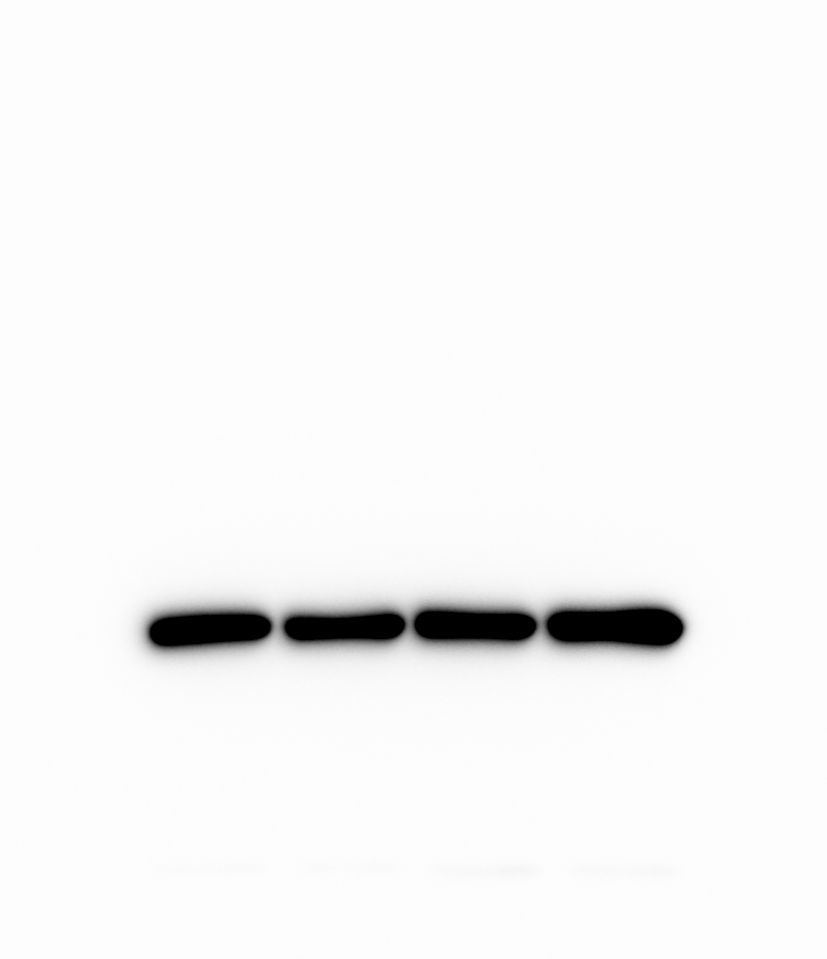

Supplement: Supplementary file 2 [file DataSheet1.zip › Original Western blot images collection/Experiment 3 Chemiluminescence image GAPDH.tif]

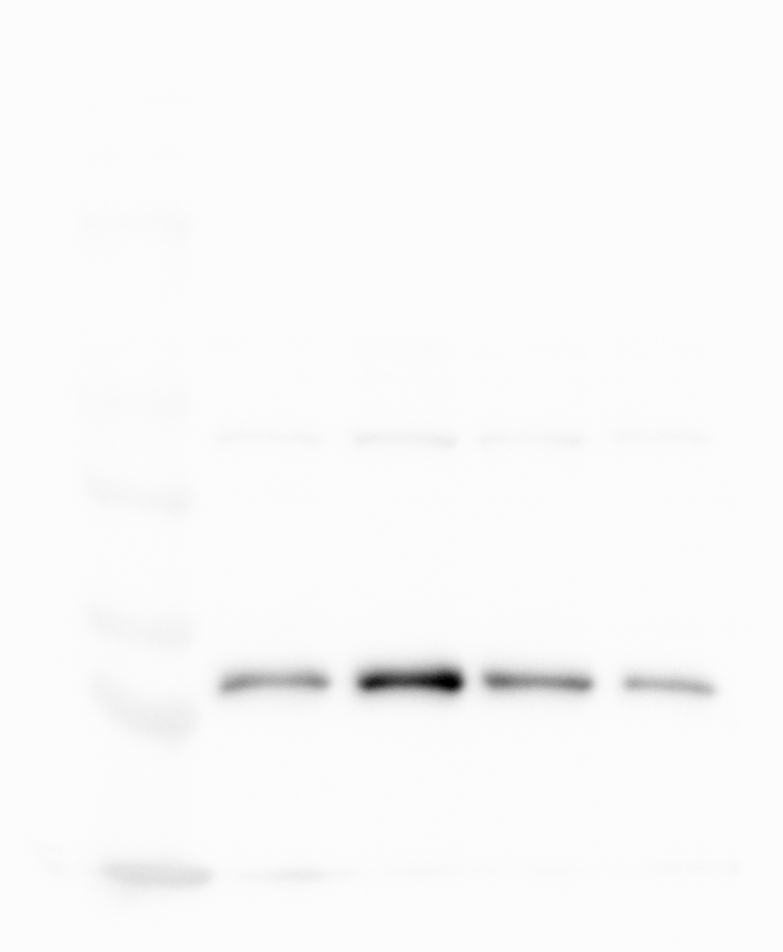

Supplement: Supplementary file 2 [file DataSheet1.zip › Original Western blot images collection/Experiment 3 Chemiluminescence image INSIG1.tif]

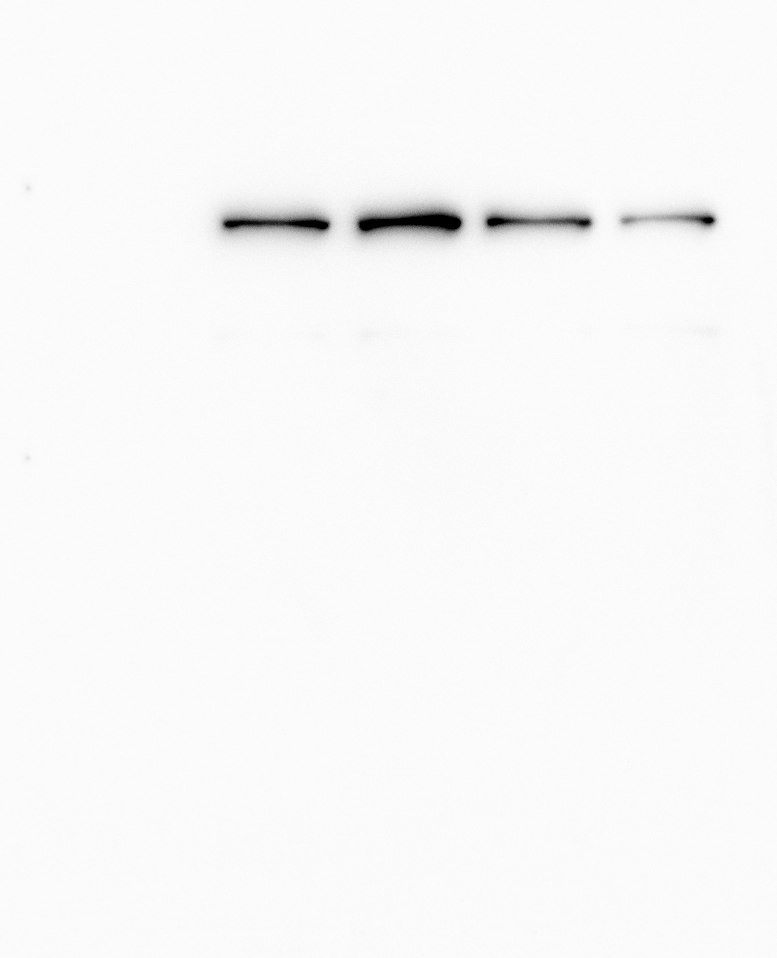

Supplement: Supplementary file 2 [file DataSheet1.zip › Original Western blot images collection/Experiment 3 Chemiluminescence image SREBP1.tif]

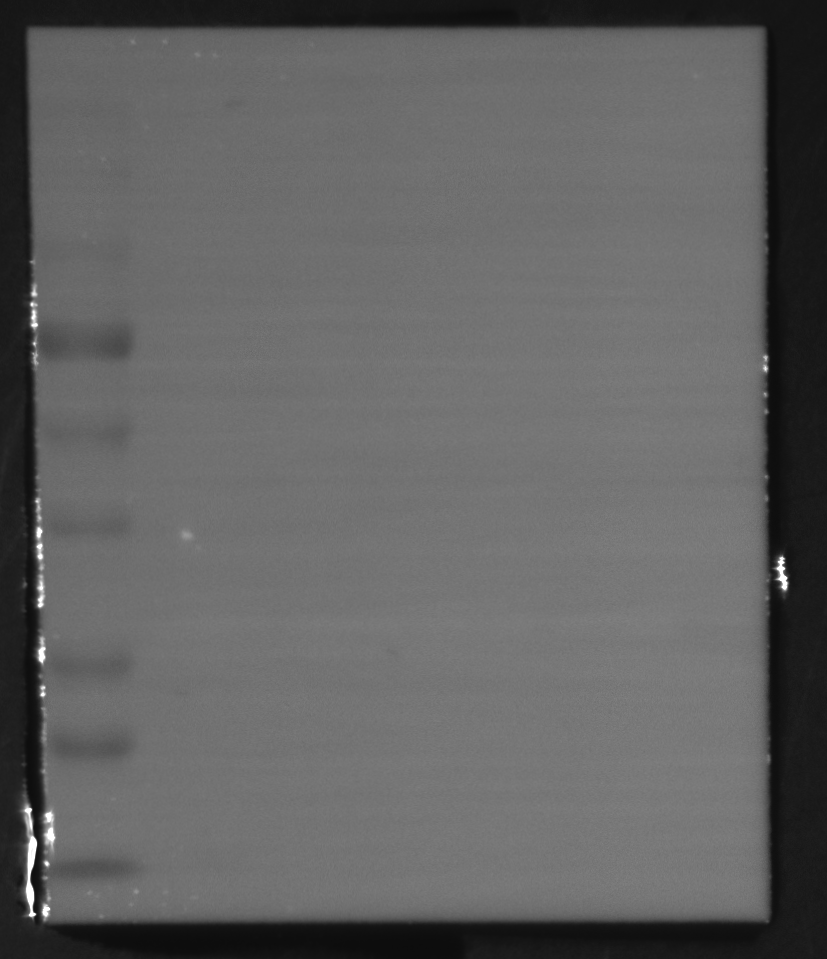

Supplement: Supplementary file 2 [file DataSheet1.zip › Original Western blot images collection/Experiment 3 White light image GAPDH.tif]

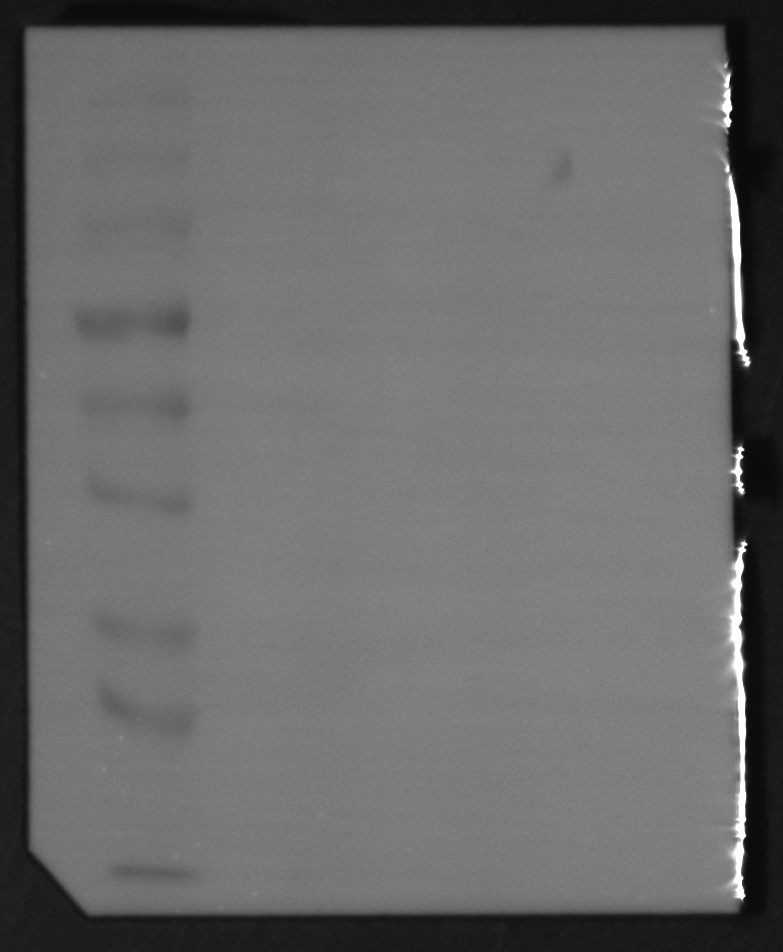

Supplement: Supplementary file 2 [file DataSheet1.zip › Original Western blot images collection/Experiment 3 White light image INSIG1.tif]

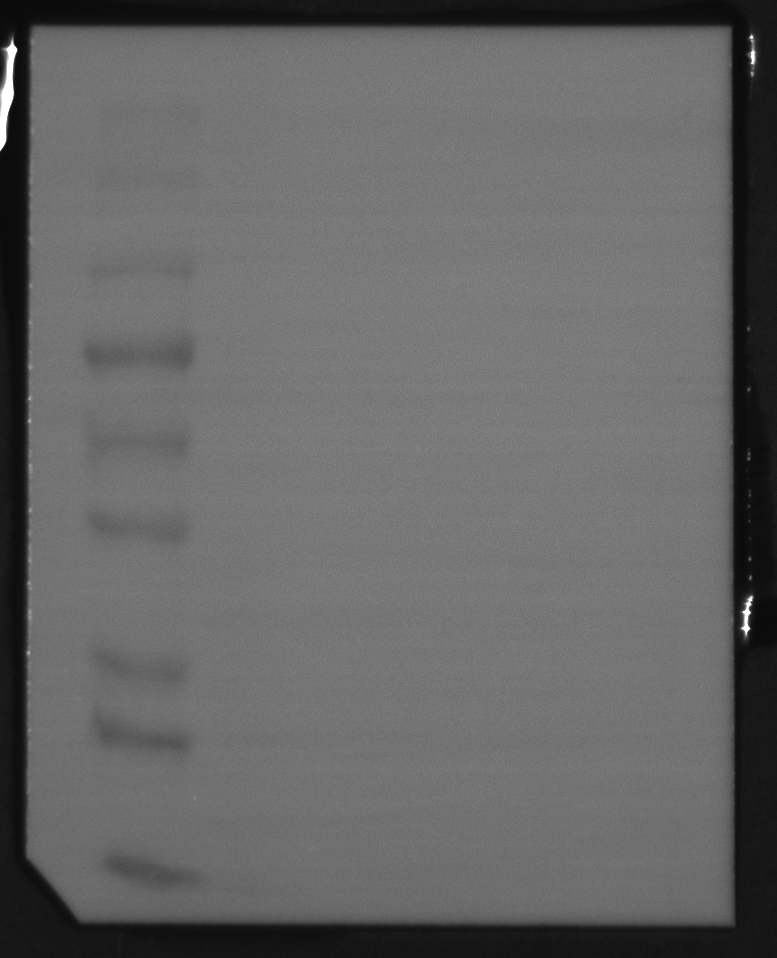

Supplement: Supplementary file 2 [file DataSheet1.zip › Original Western blot images collection/Experiment 3 White light image SREBP1.tif]

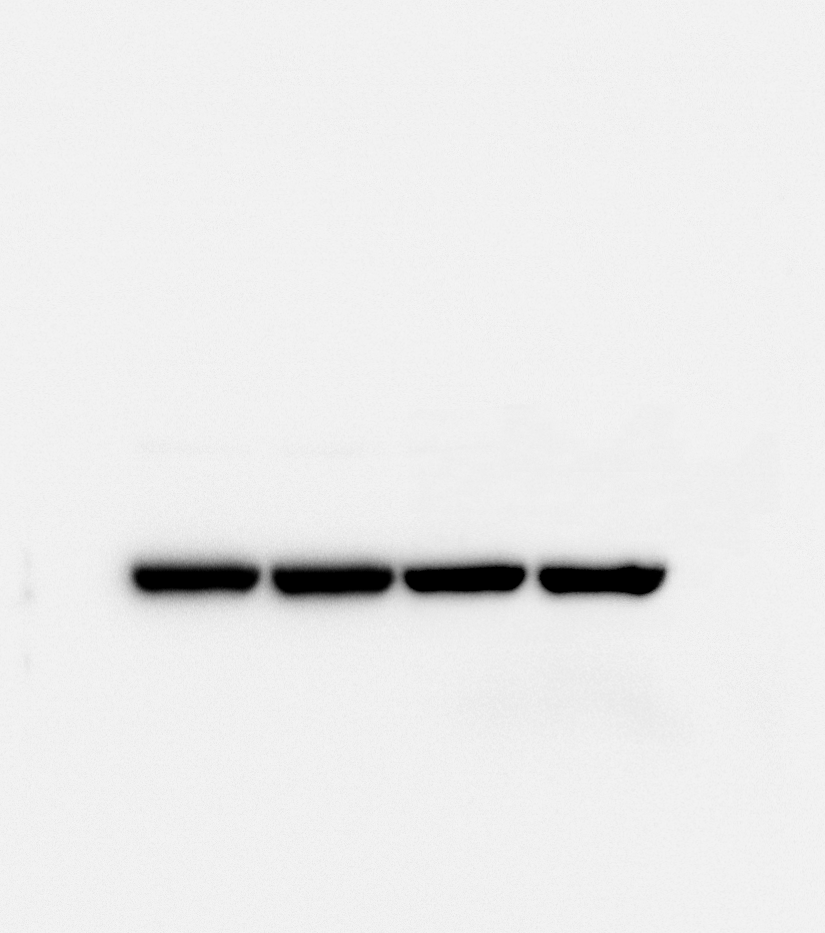

Supplement: Supplementary file 2 [file DataSheet1.zip › Original Western blot images collection/Experiment 4 Chemiluminescence image GAPDH U20S).tif]

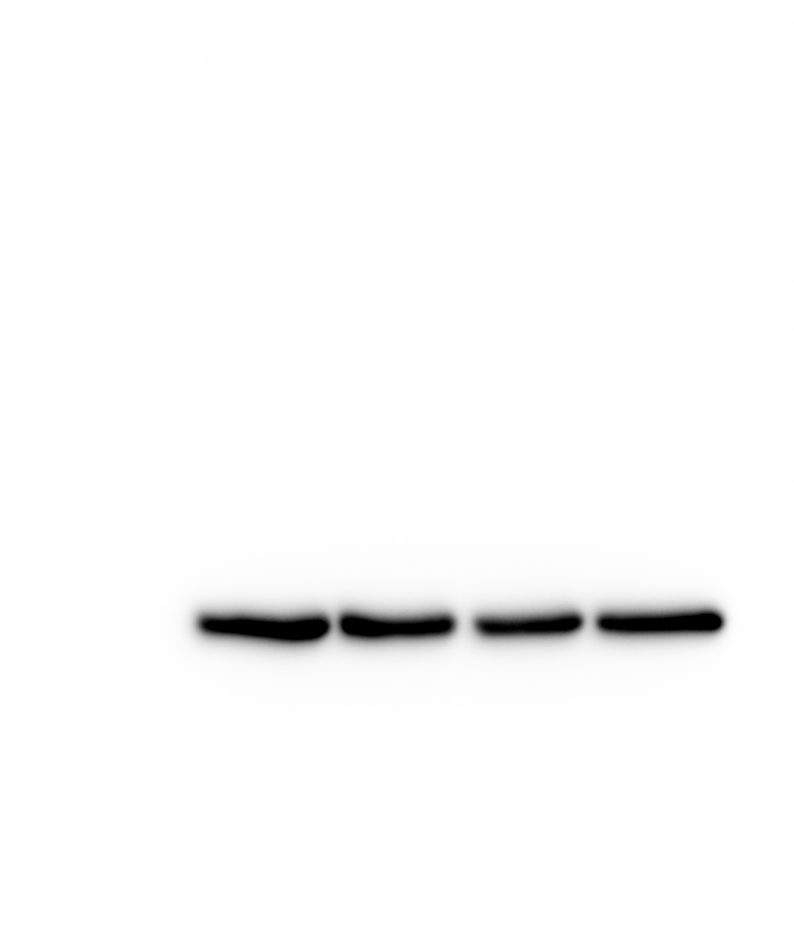

Supplement: Supplementary file 2 [file DataSheet1.zip › Original Western blot images collection/Experiment 4 Chemiluminescence image GAPDH(MG63).tif]

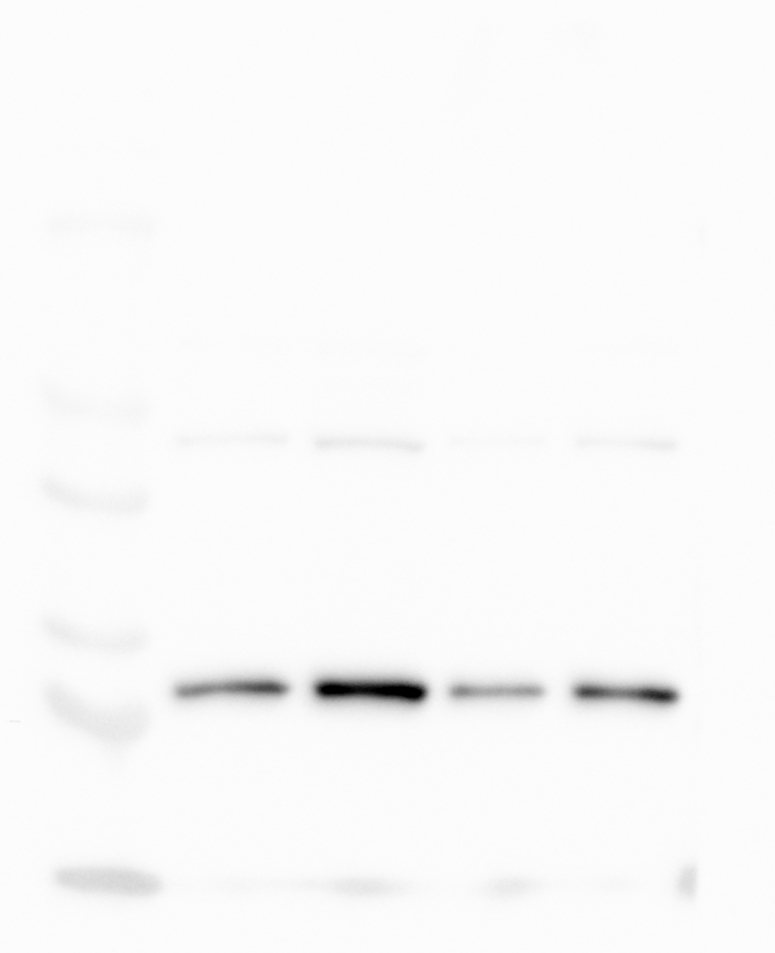

Supplement: Supplementary file 2 [file DataSheet1.zip › Original Western blot images collection/Experiment 4 Chemiluminescence image INSIG1(MG63).tif]

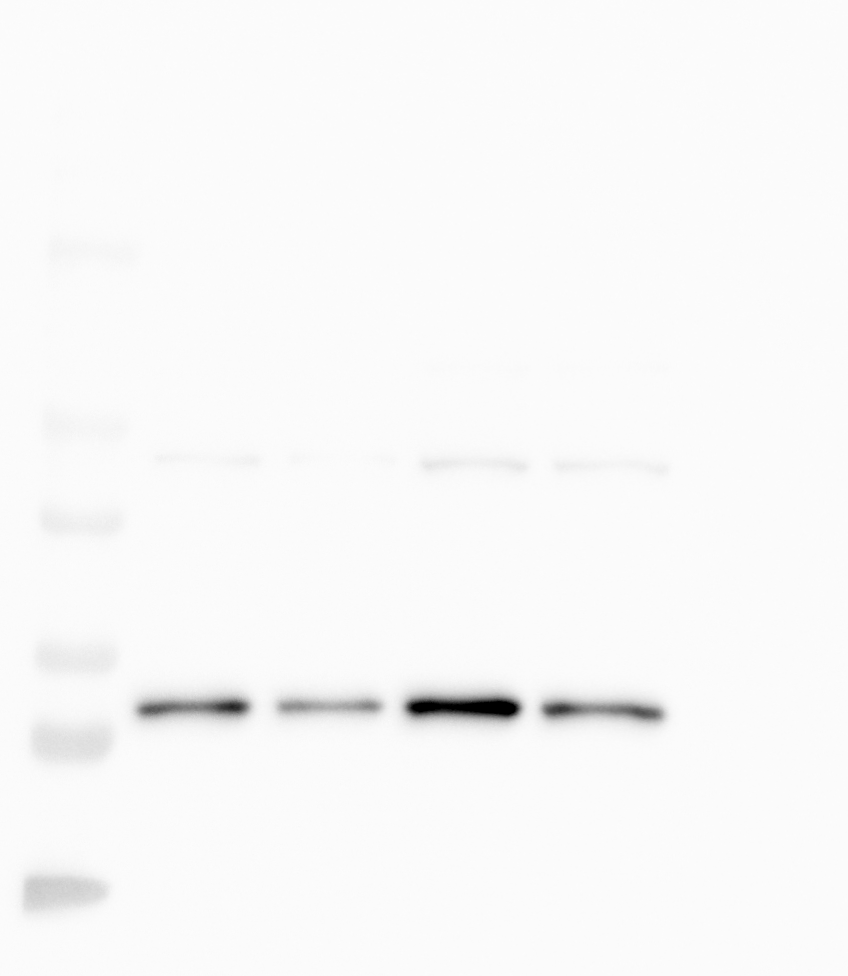

Supplement: Supplementary file 2 [file DataSheet1.zip › Original Western blot images collection/Experiment 4 Chemiluminescence image INSIG1(U20S).tif]

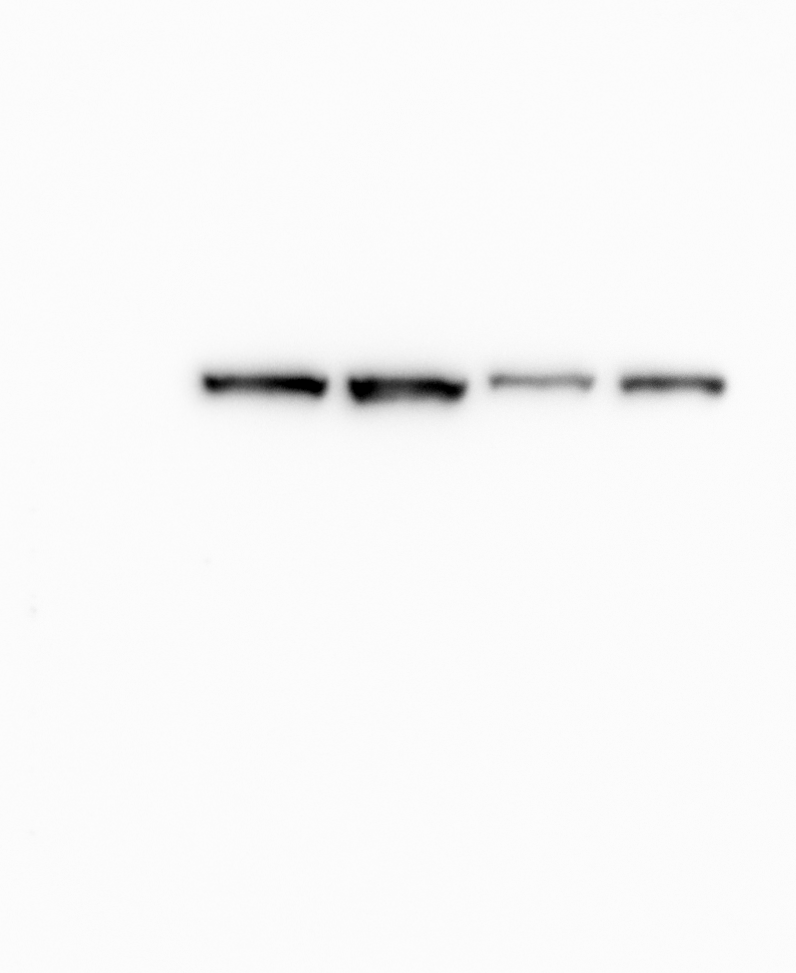

Supplement: Supplementary file 2 [file DataSheet1.zip › Original Western blot images collection/Experiment 4 Chemiluminescence image LPCAT1(MG63).tif]

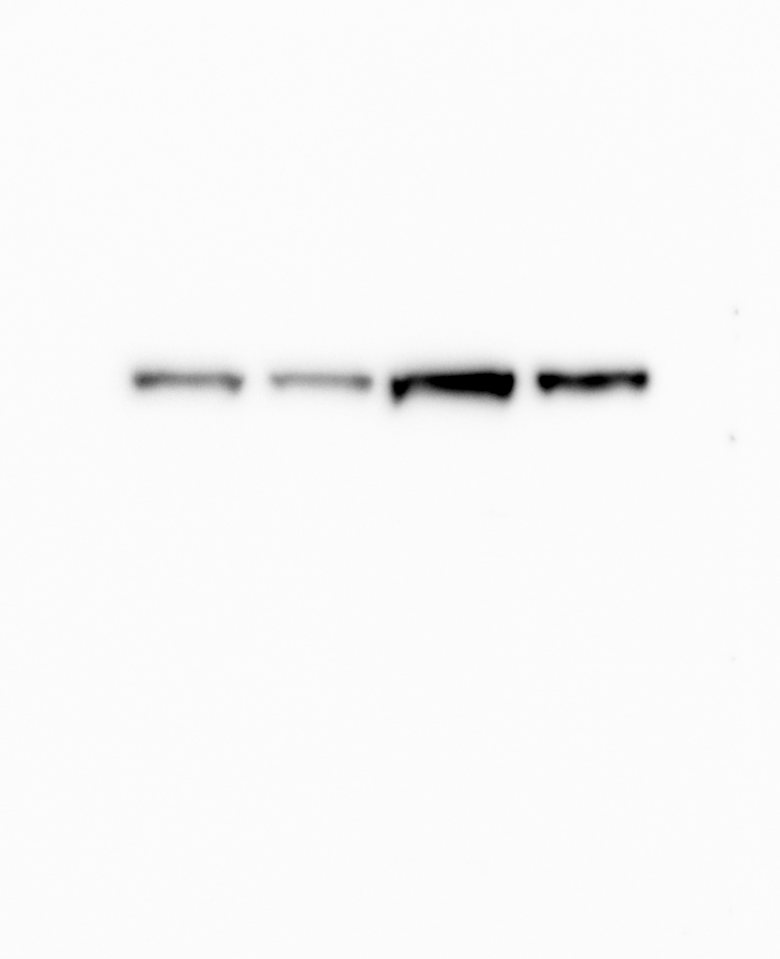

Supplement: Supplementary file 2 [file DataSheet1.zip › Original Western blot images collection/Experiment 4 Chemiluminescence image LPCAT1(U20S).tif]

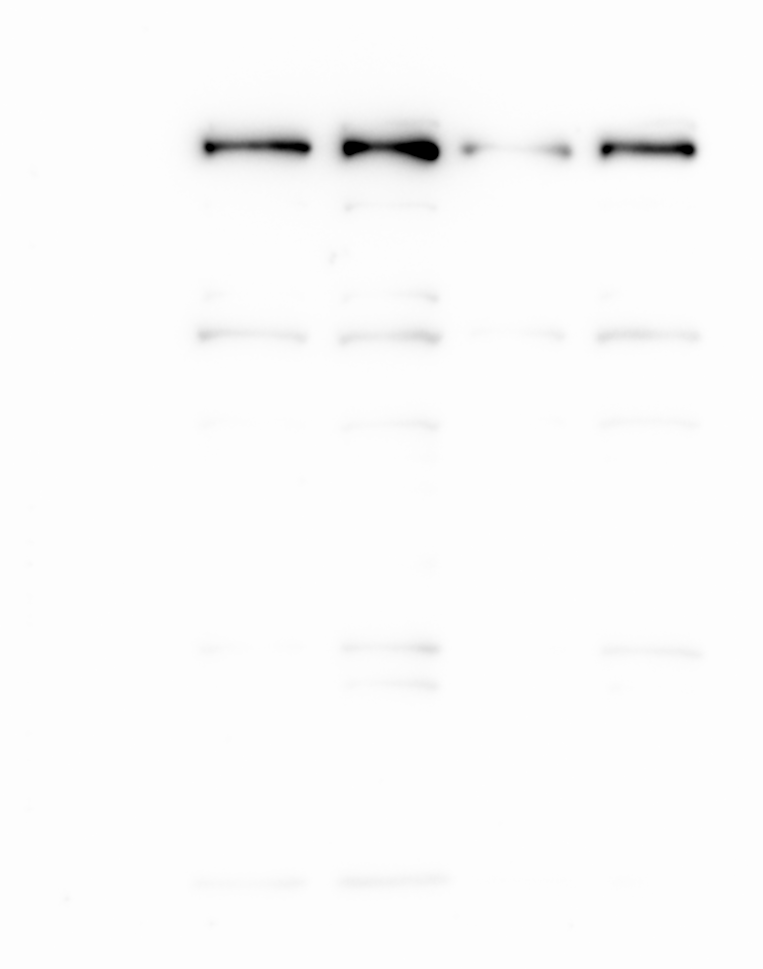

Supplement: Supplementary file 2 [file DataSheet1.zip › Original Western blot images collection/Experiment 4 Chemiluminescence image N-cadherin(MG63).tif]

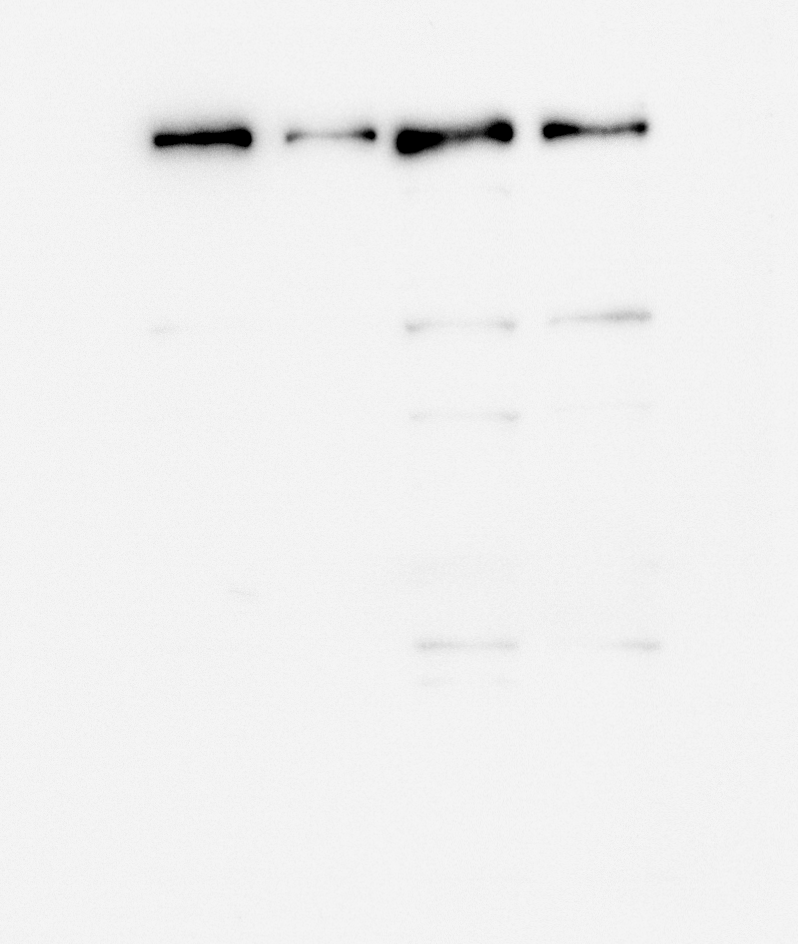

Supplement: Supplementary file 2 [file DataSheet1.zip › Original Western blot images collection/Experiment 4 Chemiluminescence image N-cadherin(U20S).tif]

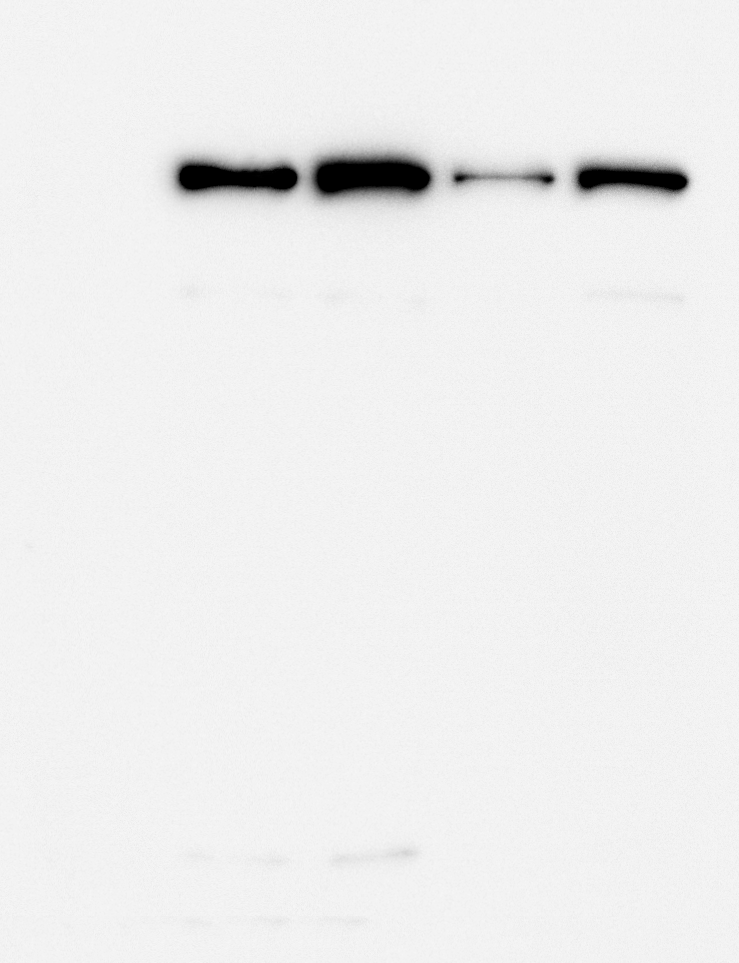

Supplement: Supplementary file 2 [file DataSheet1.zip › Original Western blot images collection/Experiment 4 Chemiluminescence image SREBP1(MG63).tif]

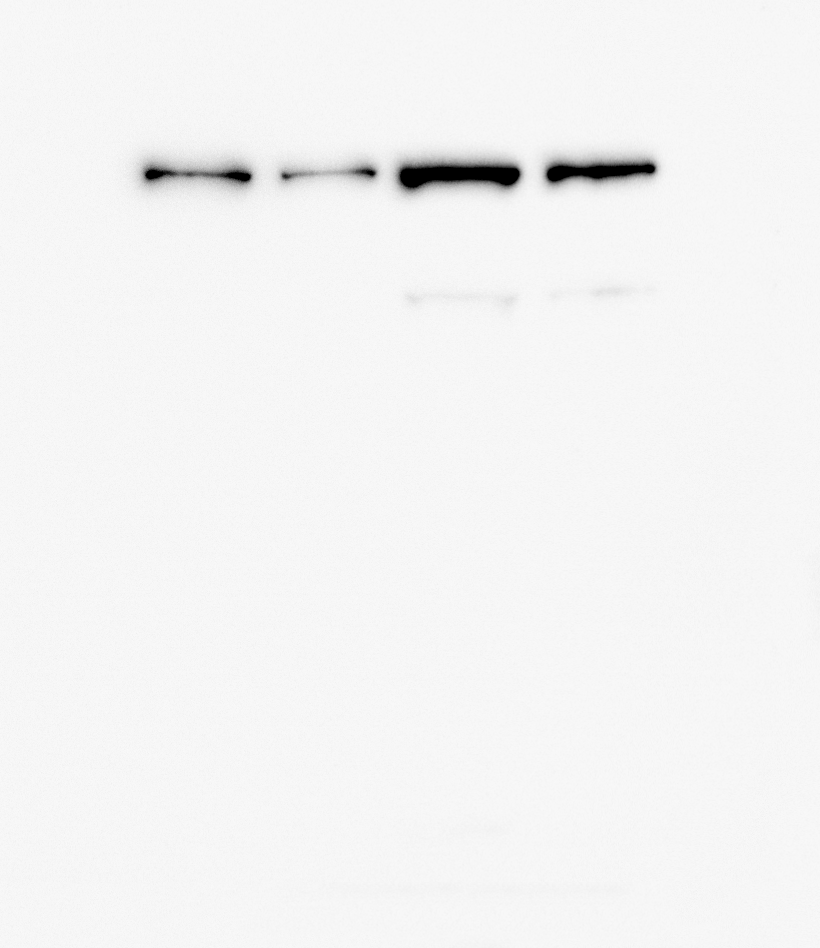

Supplement: Supplementary file 2 [file DataSheet1.zip › Original Western blot images collection/Experiment 4 Chemiluminescence image SREBP1(U20S).tif]

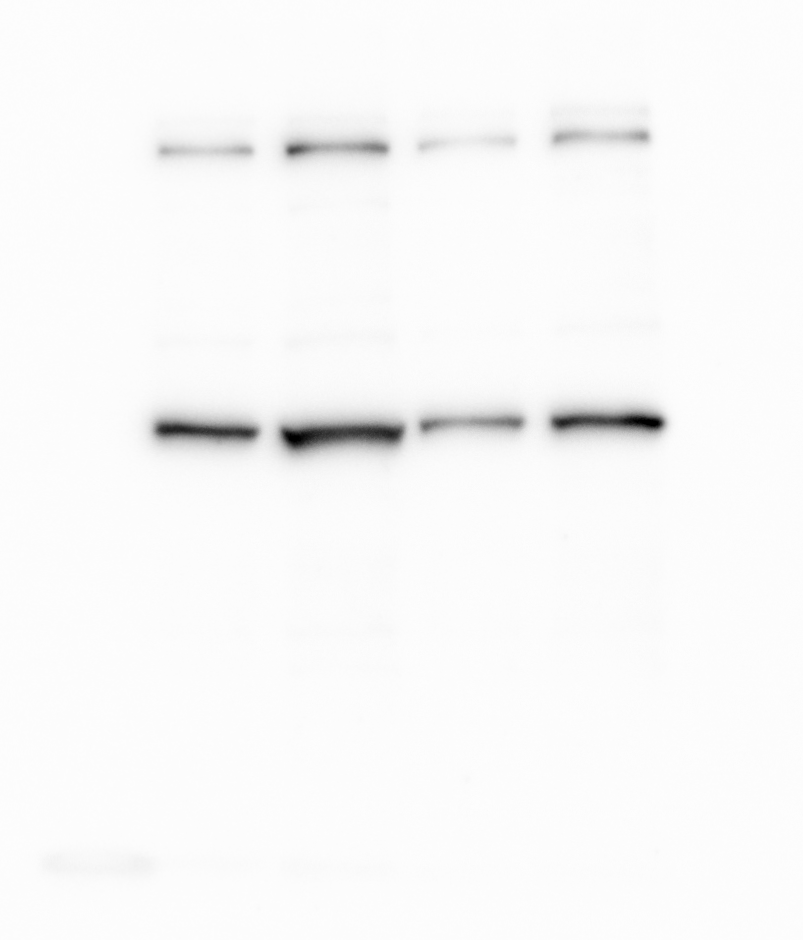

Supplement: Supplementary file 2 [file DataSheet1.zip › Original Western blot images collection/Experiment 4 Chemiluminescence image Vimentin(MG63).tif]

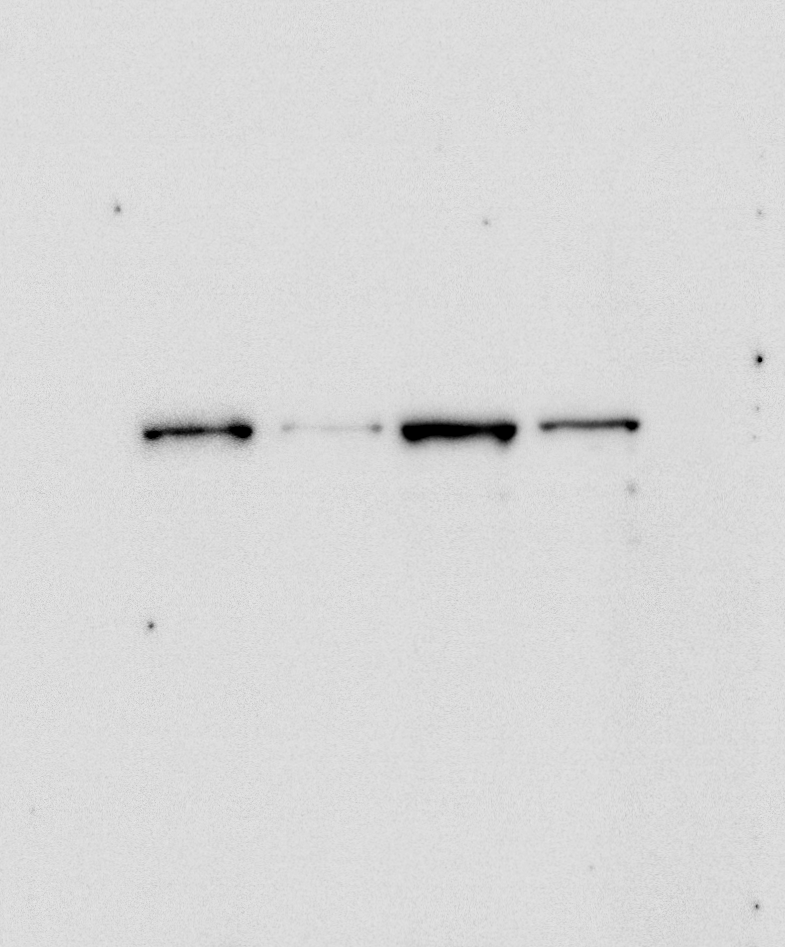

Supplement: Supplementary file 2 [file DataSheet1.zip › Original Western blot images collection/Experiment 4 Chemiluminescence image Vimentin(U20S).tif]

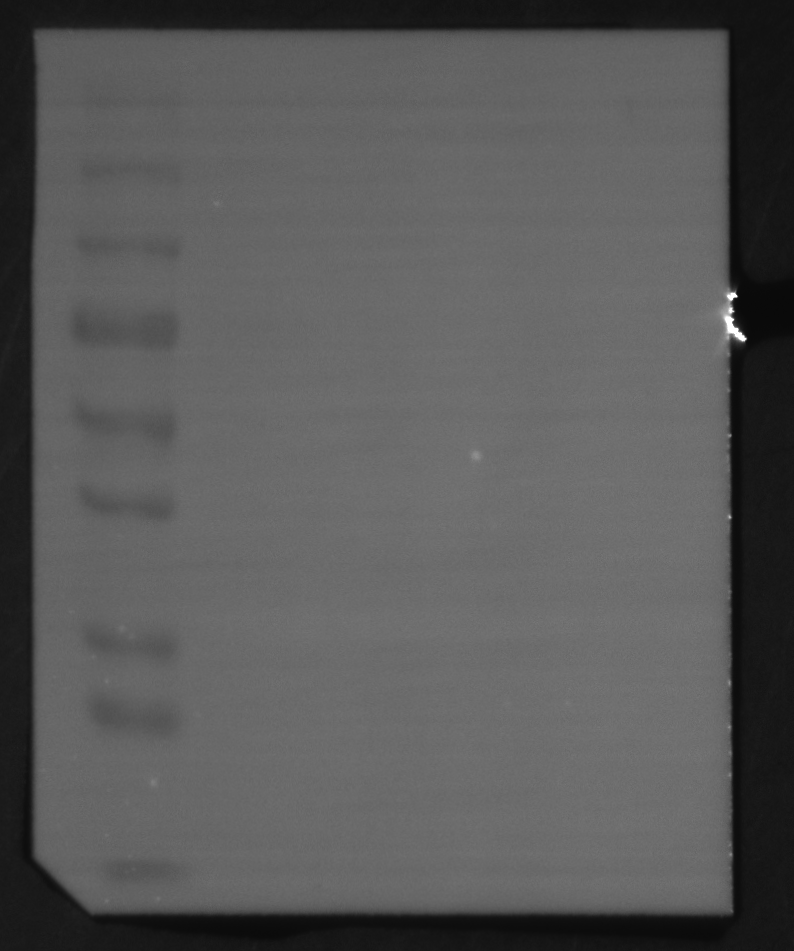

Supplement: Supplementary file 2 [file DataSheet1.zip › Original Western blot images collection/Experiment 4 White light image GAPDH(MG63).tif]

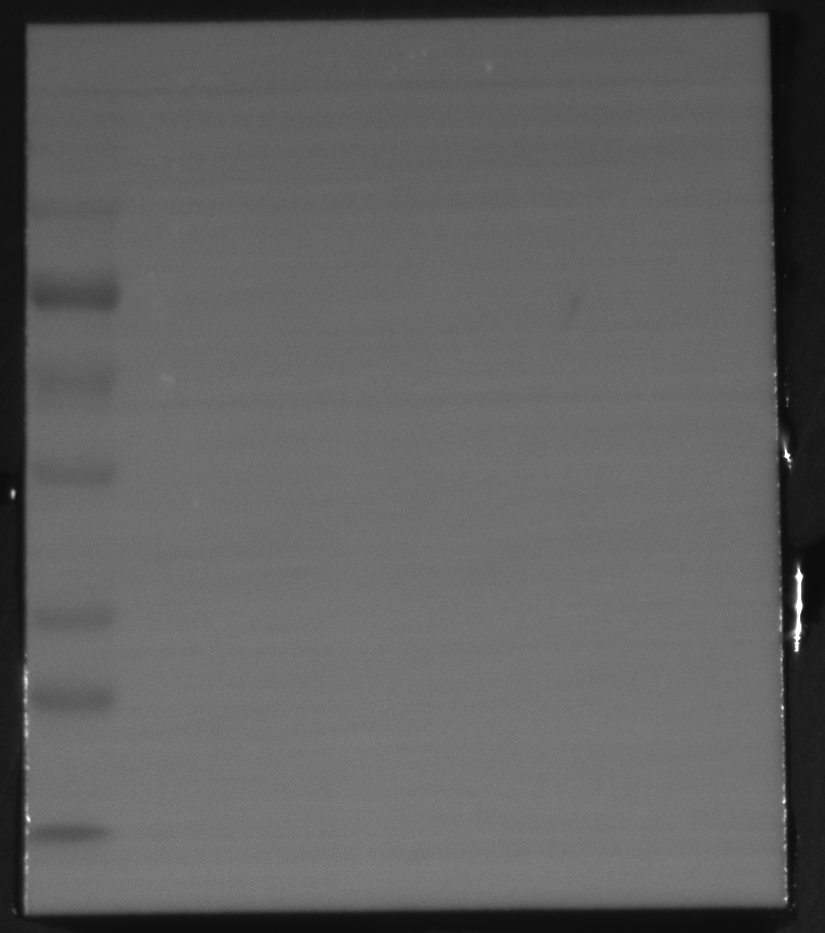

Supplement: Supplementary file 2 [file DataSheet1.zip › Original Western blot images collection/Experiment 4 White light image GAPDH(U20S).tif]

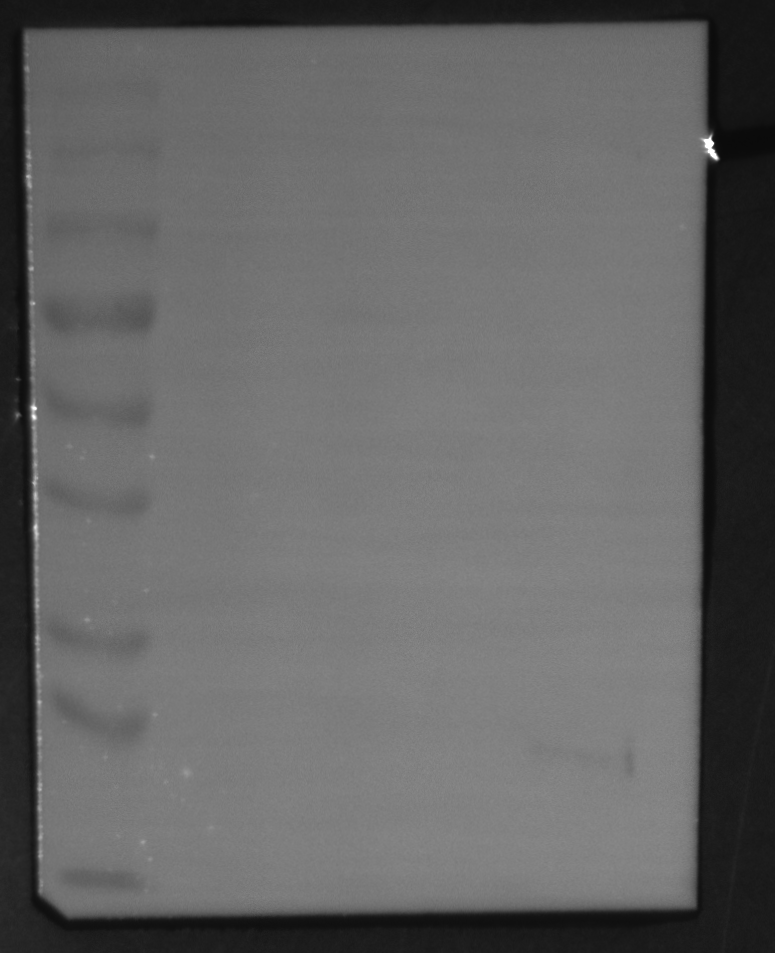

Supplement: Supplementary file 2 [file DataSheet1.zip › Original Western blot images collection/Experiment 4 White light image INSIG1(MG63).tif]

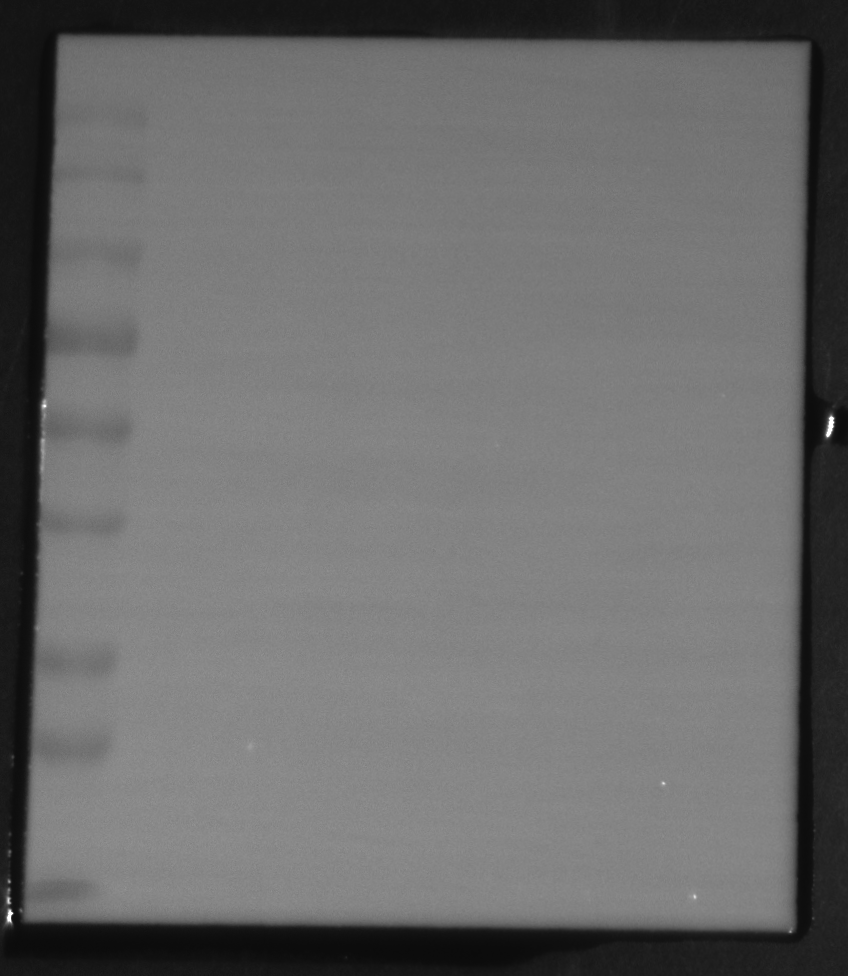

Supplement: Supplementary file 2 [file DataSheet1.zip › Original Western blot images collection/Experiment 4 White light image INSIG1(U20S).tif]

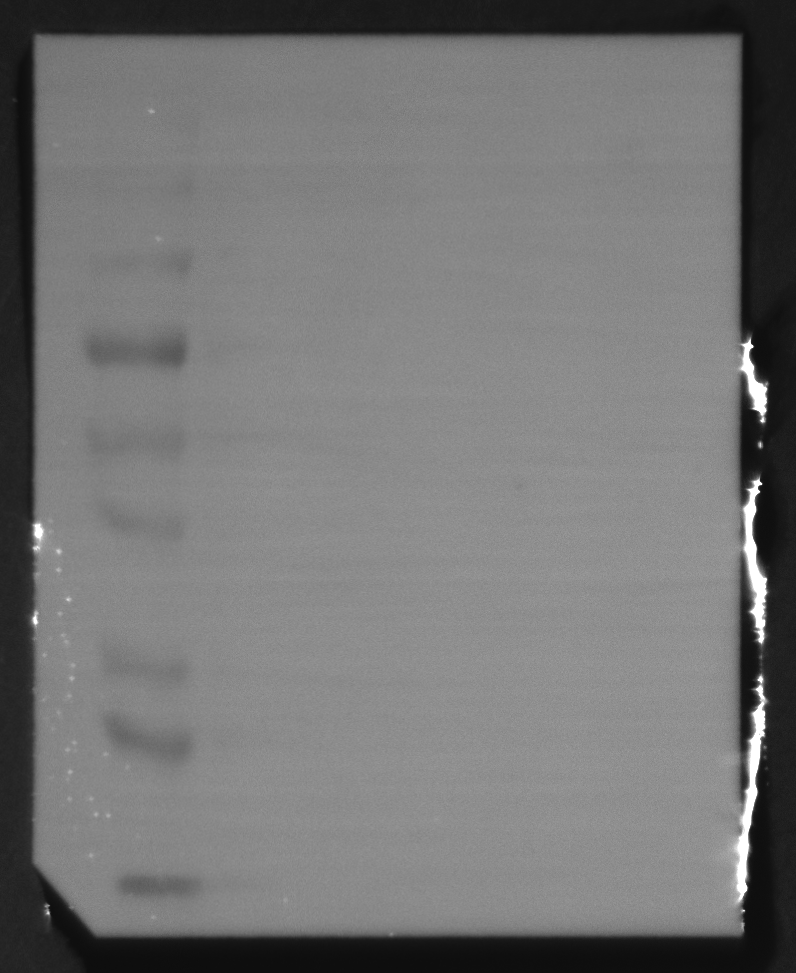

Supplement: Supplementary file 2 [file DataSheet1.zip › Original Western blot images collection/Experiment 4 White light image LPCAT1(MG63).tif]

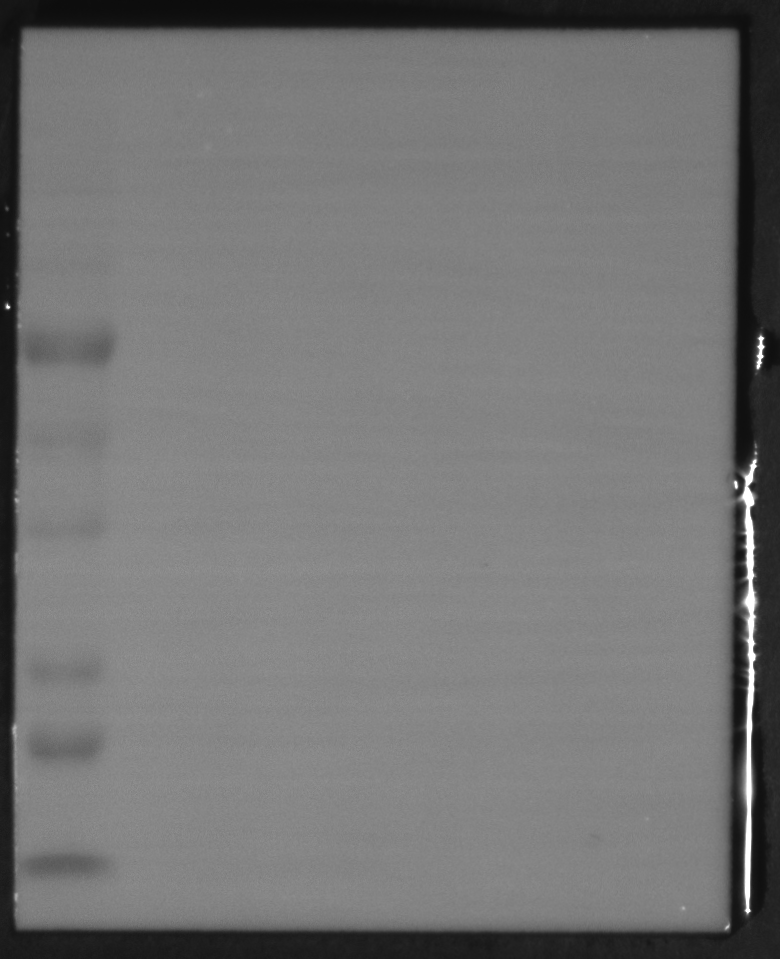

Supplement: Supplementary file 2 [file DataSheet1.zip › Original Western blot images collection/Experiment 4 White light image LPCAT1(U20S).tif]

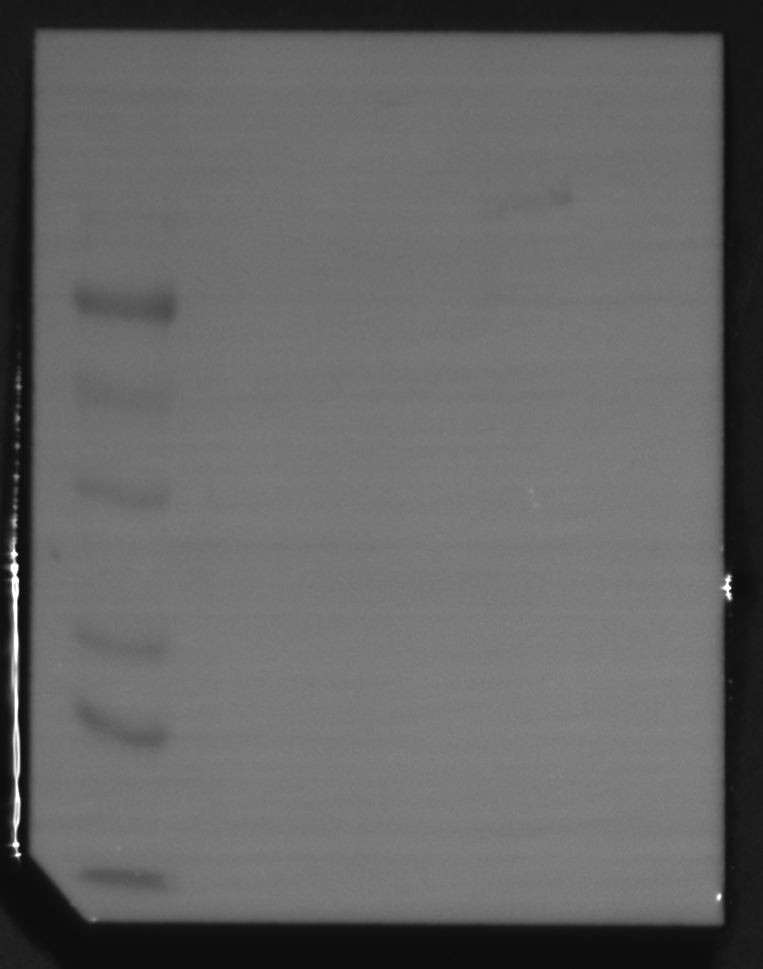

Supplement: Supplementary file 2 [file DataSheet1.zip › Original Western blot images collection/Experiment 4 White light image N-cadherin(MG63).tif]

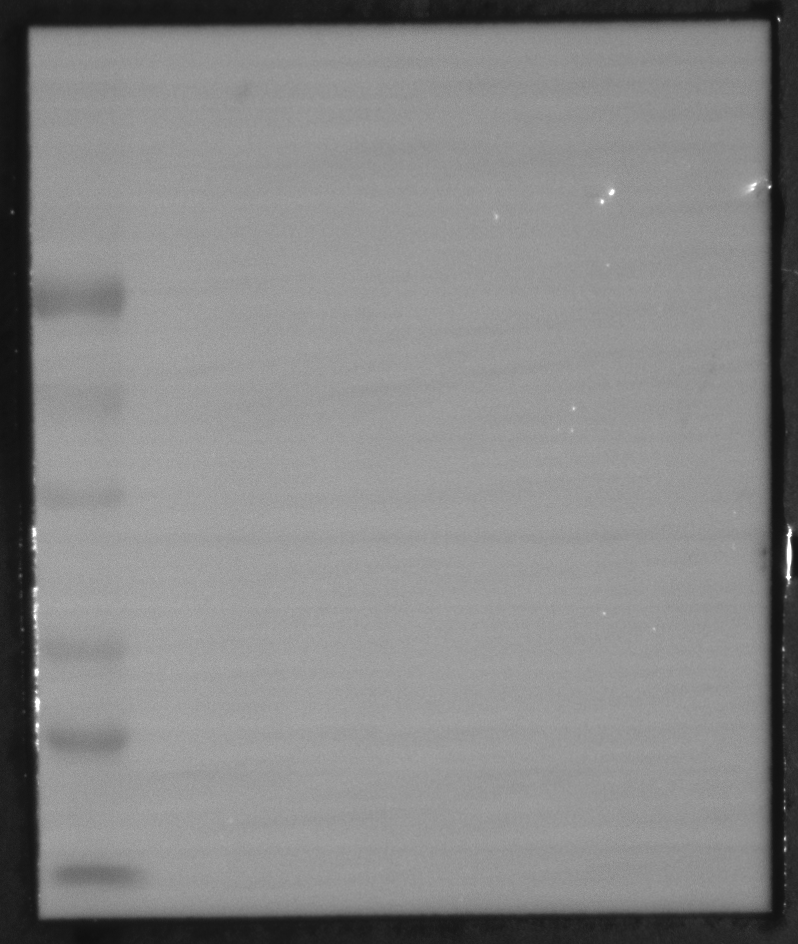

Supplement: Supplementary file 2 [file DataSheet1.zip › Original Western blot images collection/Experiment 4 White light image N-cadherin(U20S).tif]

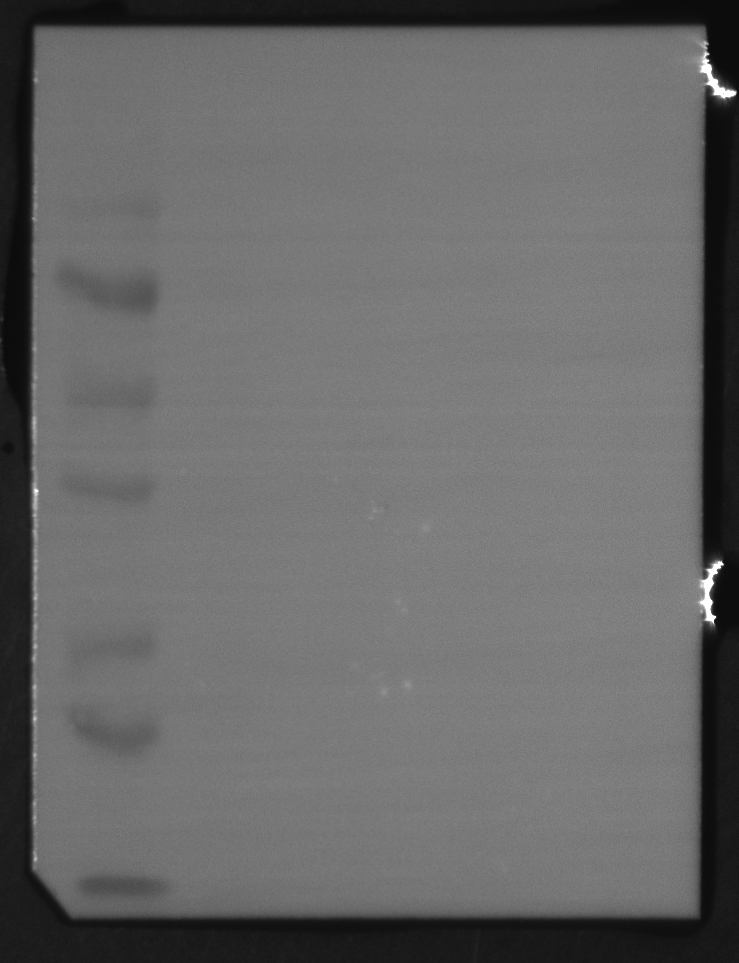

Supplement: Supplementary file 2 [file DataSheet1.zip › Original Western blot images collection/Experiment 4 White light image SREBP1(MG63).tif]

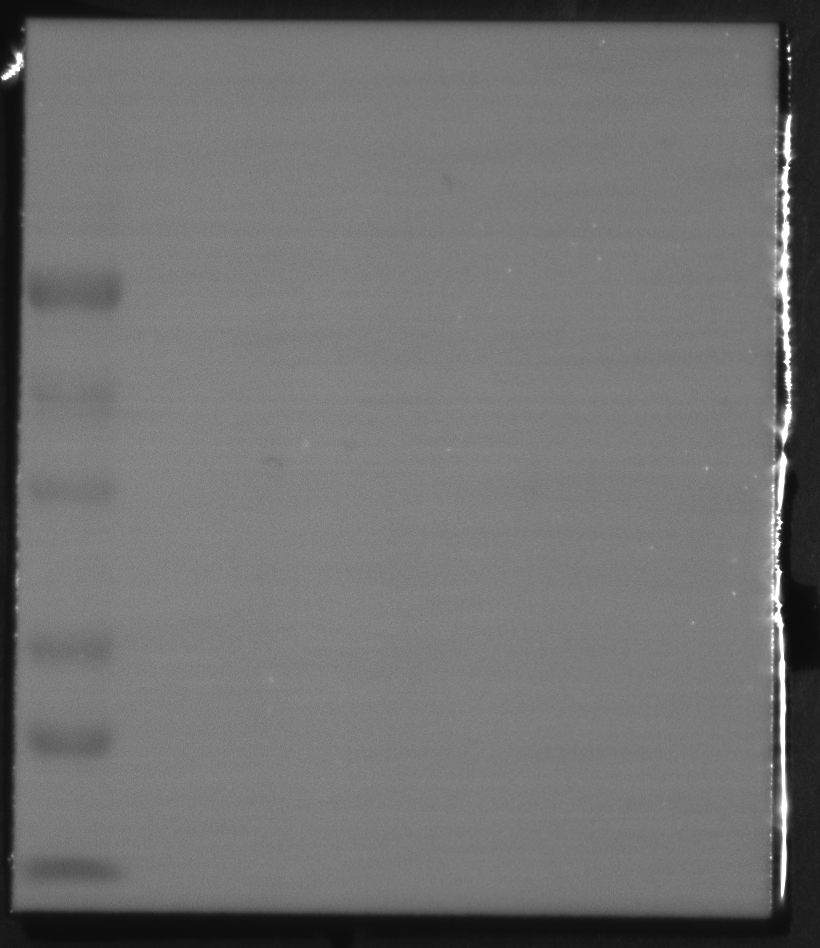

Supplement: Supplementary file 2 [file DataSheet1.zip › Original Western blot images collection/Experiment 4 White light image SREBP1(U20S).tif]

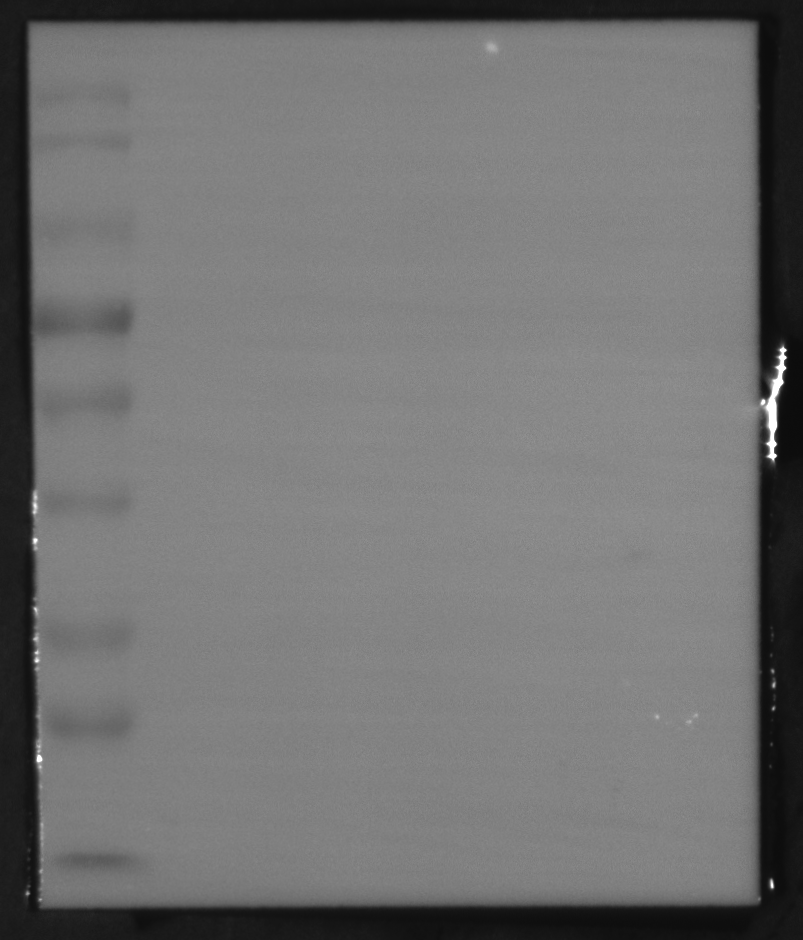

Supplement: Supplementary file 2 [file DataSheet1.zip › Original Western blot images collection/Experiment 4 White light image Vimentin(MG63).tif]

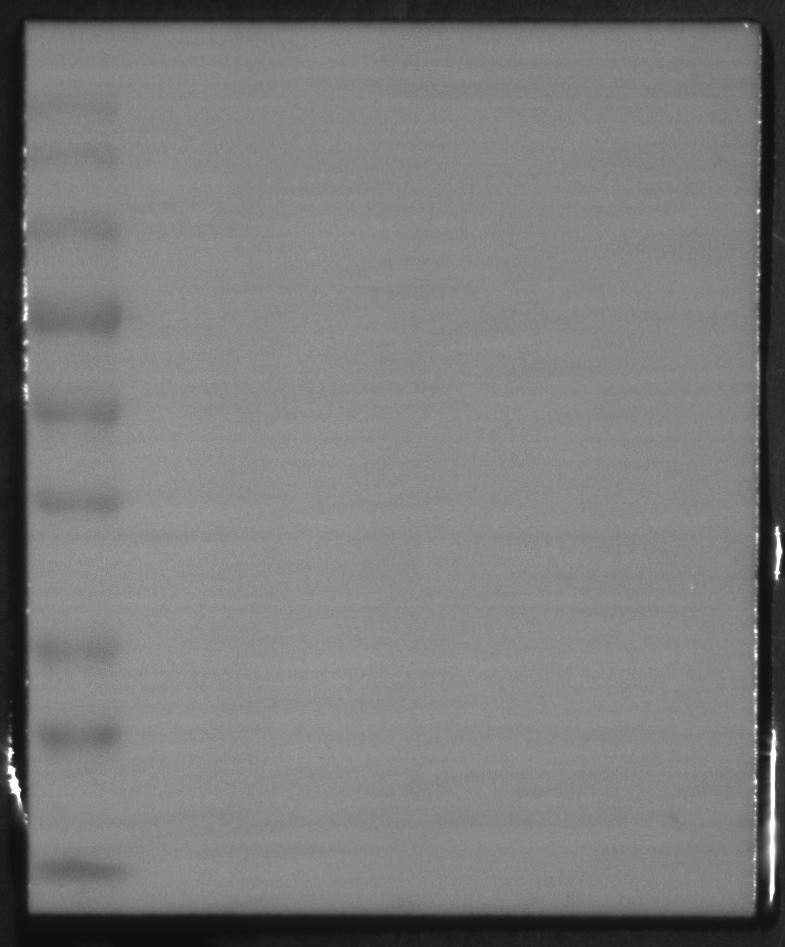

Supplement: Supplementary file 2 [file DataSheet1.zip › Original Western blot images collection/Experiment 4 White light image Vimentin(U20S).tif]

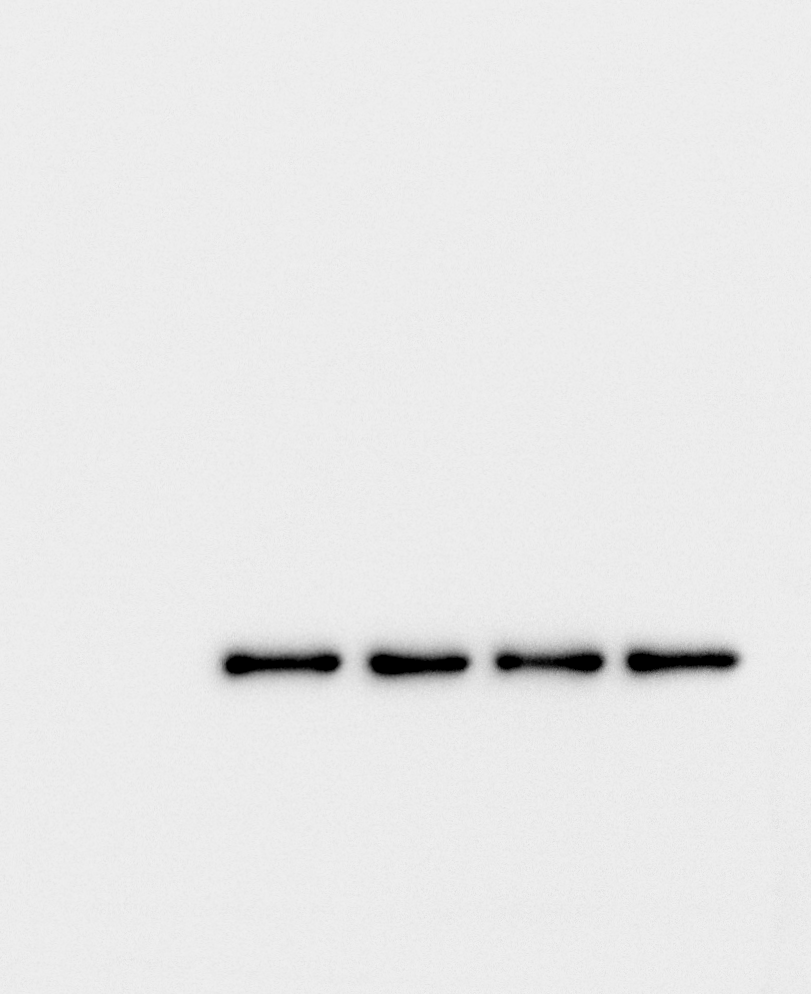

Supplement: Supplementary file 2 [file DataSheet1.zip › Original Western blot images collection/Experiment 5 Chemiluminescence image GAPDH.tif]

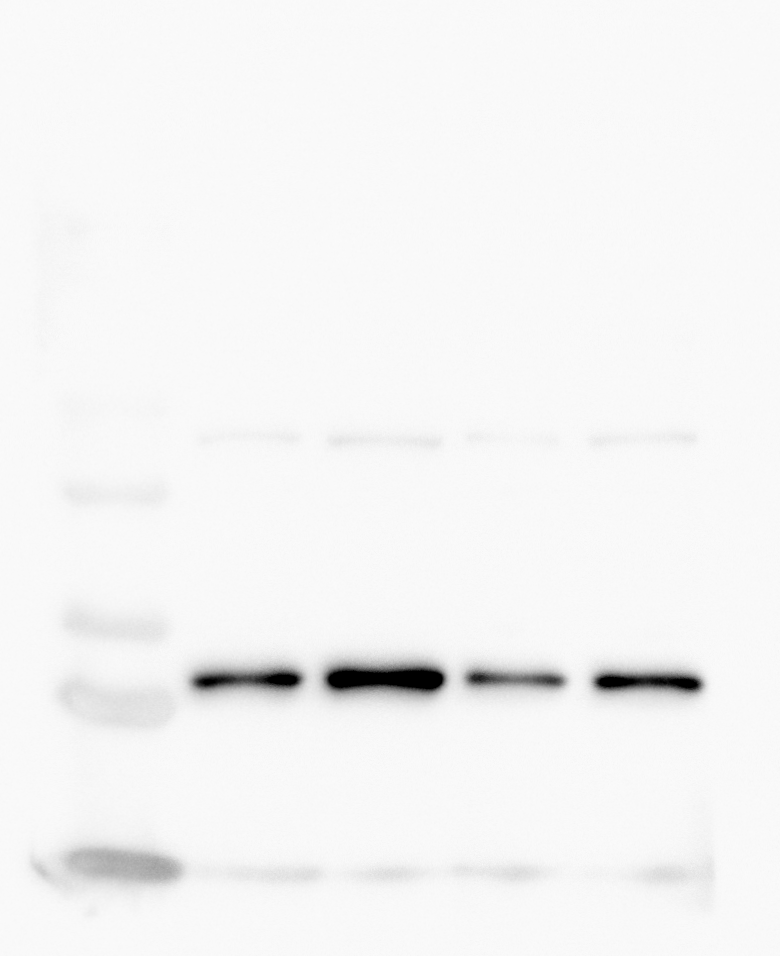

Supplement: Supplementary file 2 [file DataSheet1.zip › Original Western blot images collection/Experiment 5 Chemiluminescence image INSIG1.tif]

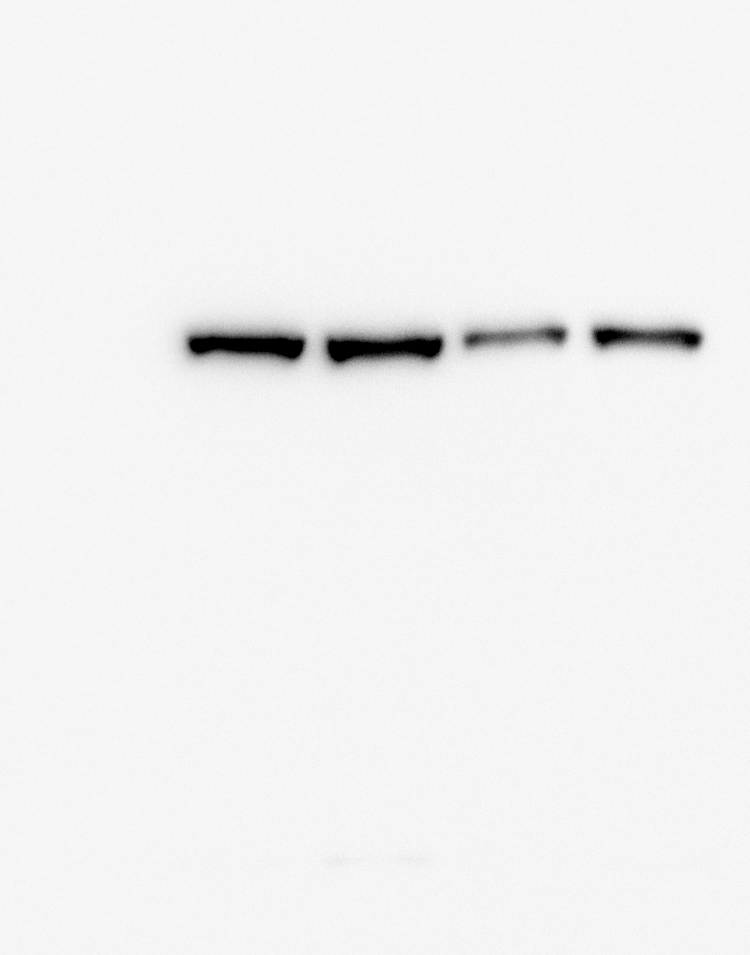

Supplement: Supplementary file 2 [file DataSheet1.zip › Original Western blot images collection/Experiment 5 Chemiluminescence image LPCAT1.tif]

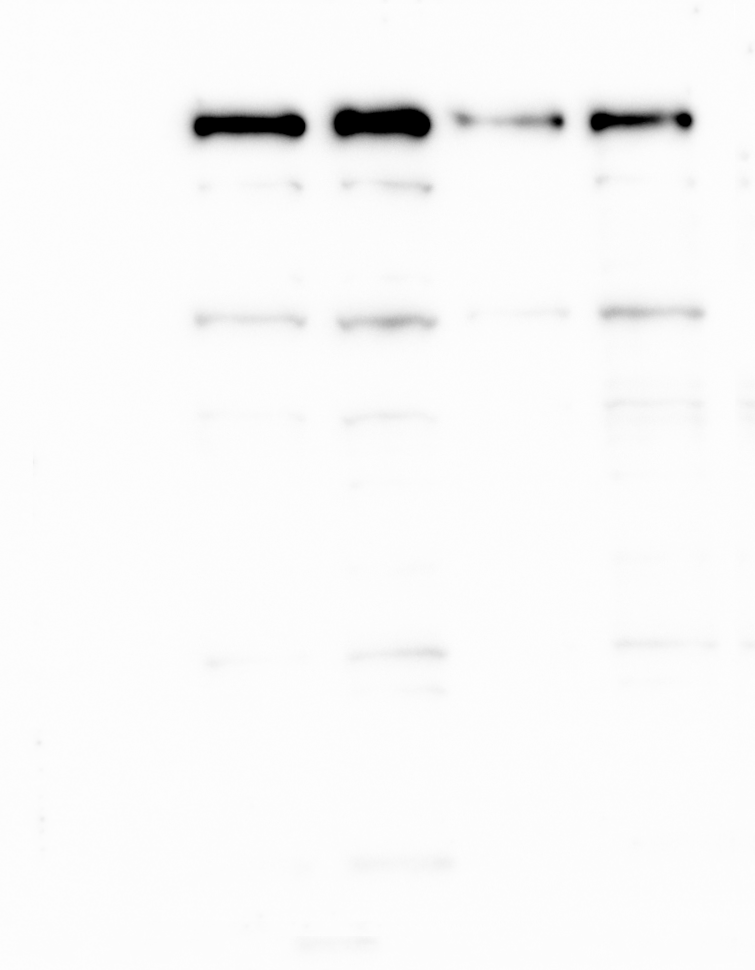

Supplement: Supplementary file 2 [file DataSheet1.zip › Original Western blot images collection/Experiment 5 Chemiluminescence image N-cadherin.tif]

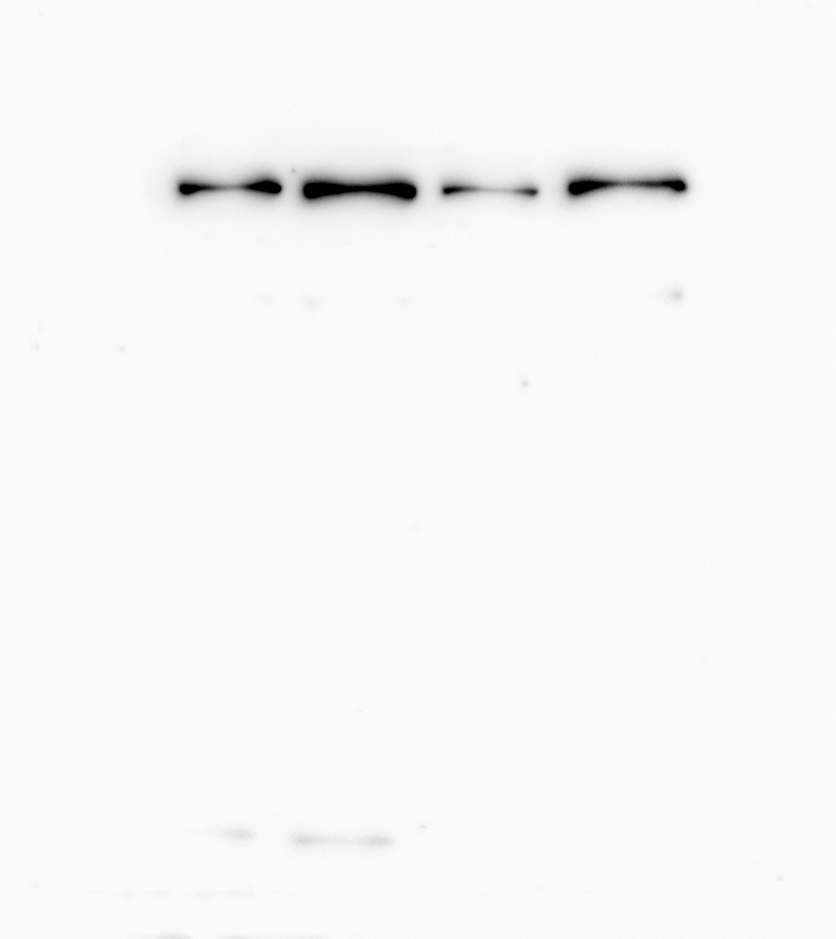

Supplement: Supplementary file 2 [file DataSheet1.zip › Original Western blot images collection/Experiment 5 Chemiluminescence image SREBP1.tif]

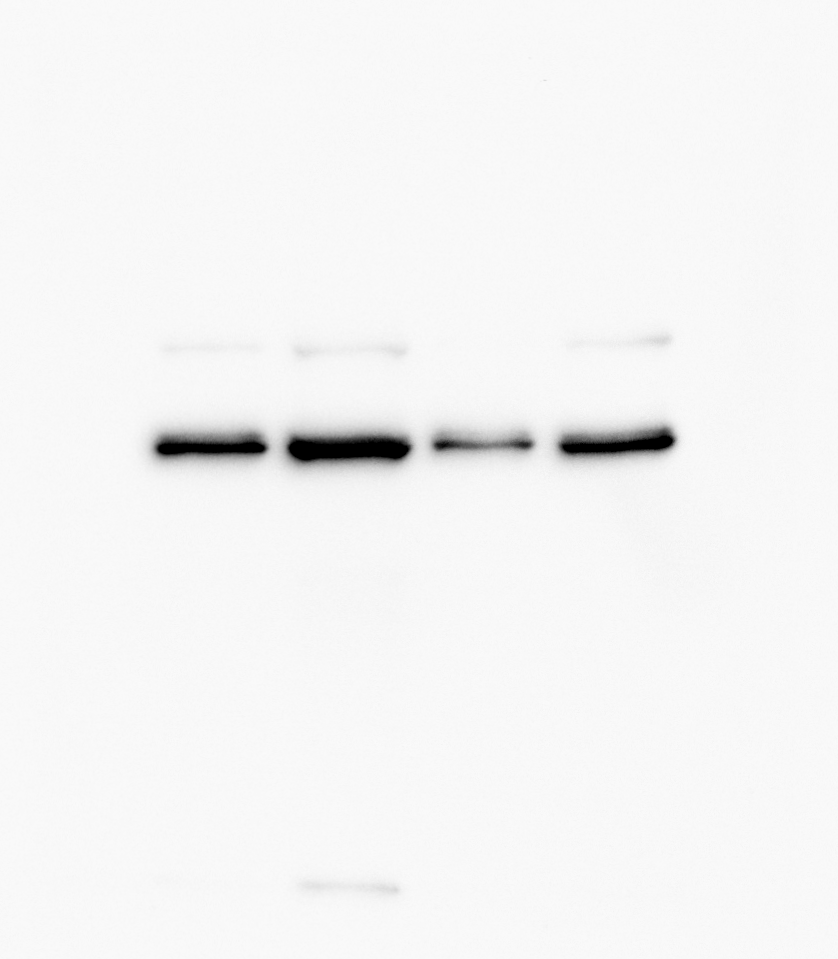

Supplement: Supplementary file 2 [file DataSheet1.zip › Original Western blot images collection/Experiment 5 Chemiluminescence image Vimentin.tif]

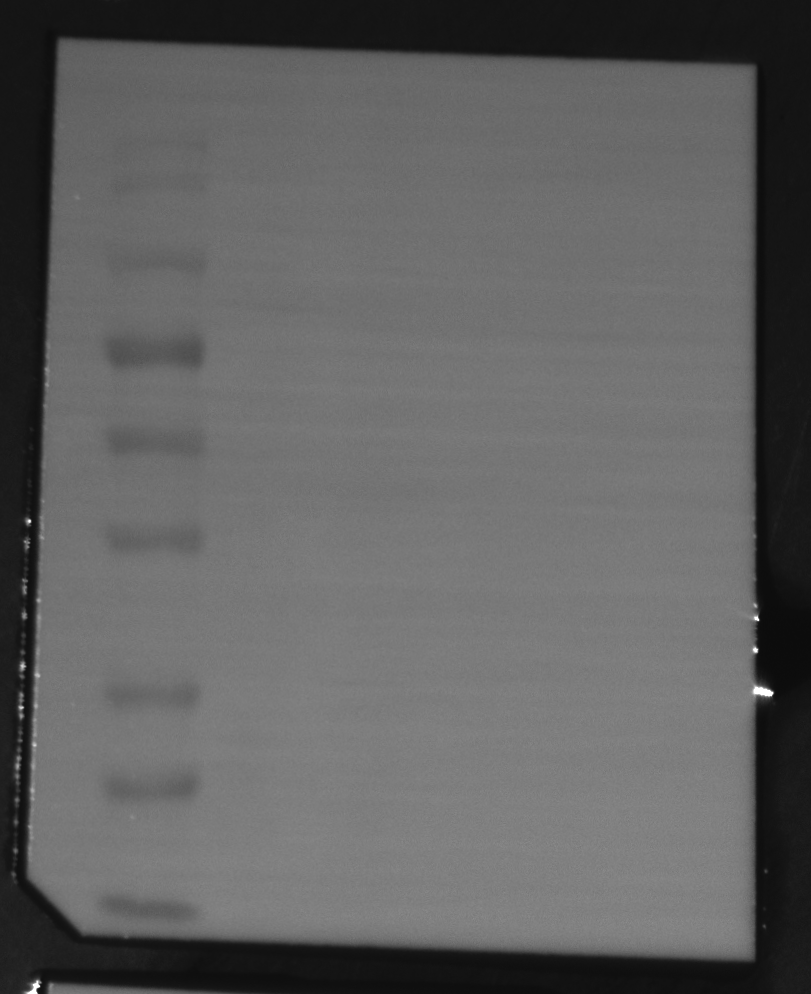

Supplement: Supplementary file 2 [file DataSheet1.zip › Original Western blot images collection/Experiment 5 White light image GAPDH.tif]

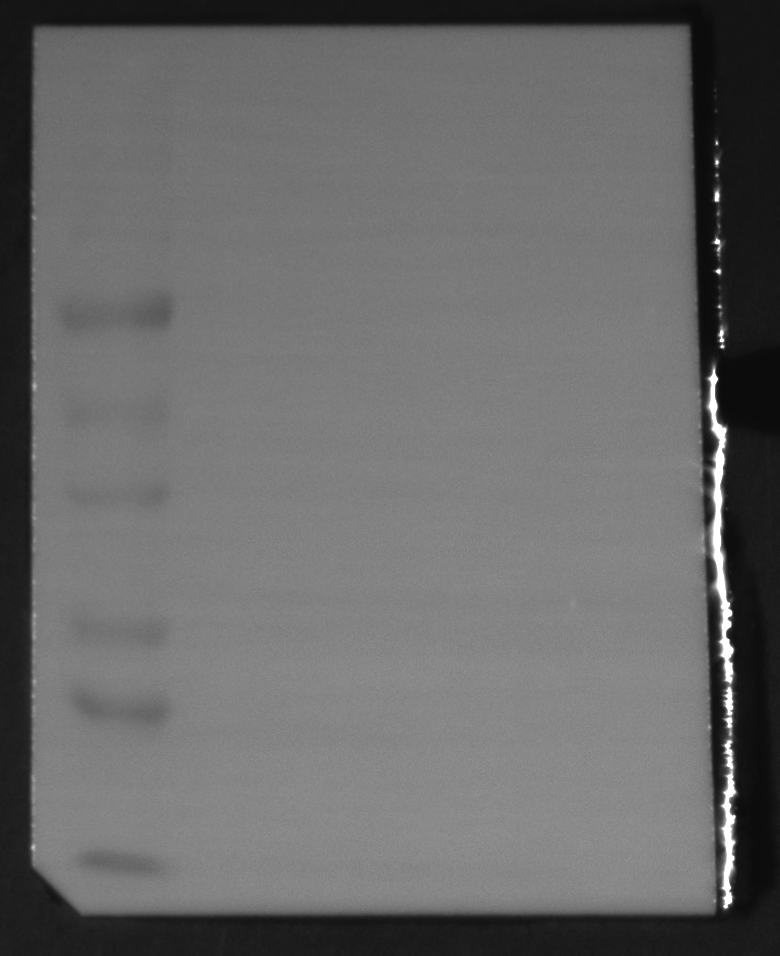

Supplement: Supplementary file 2 [file DataSheet1.zip › Original Western blot images collection/Experiment 5 White light image INSIG1.tif]

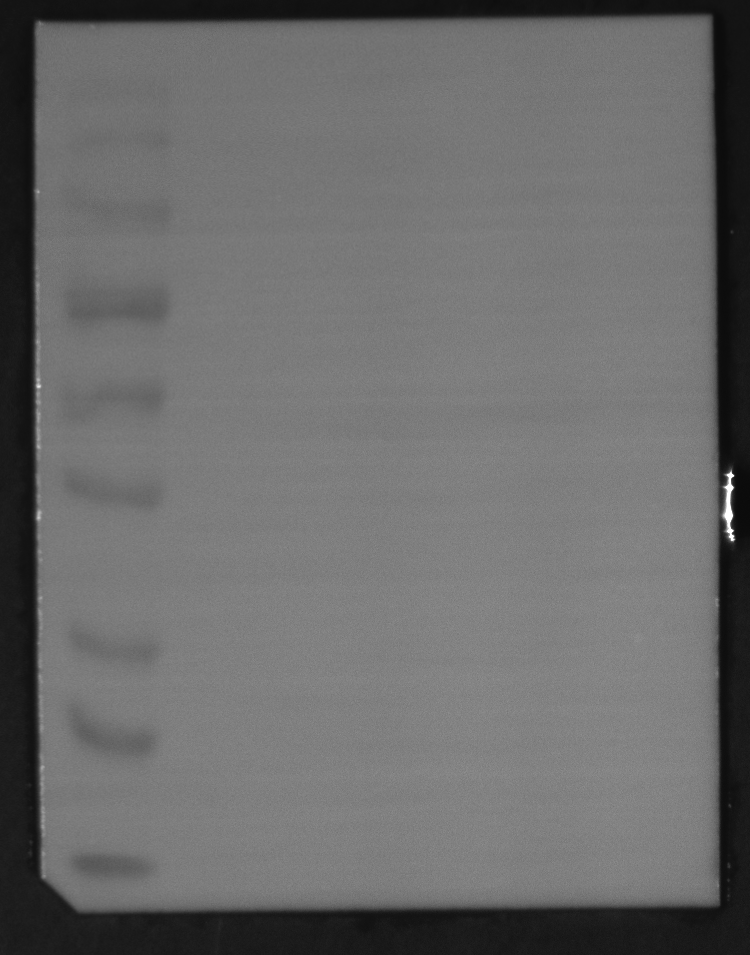

Supplement: Supplementary file 2 [file DataSheet1.zip › Original Western blot images collection/Experiment 5 White light image LPCAT1.tif]

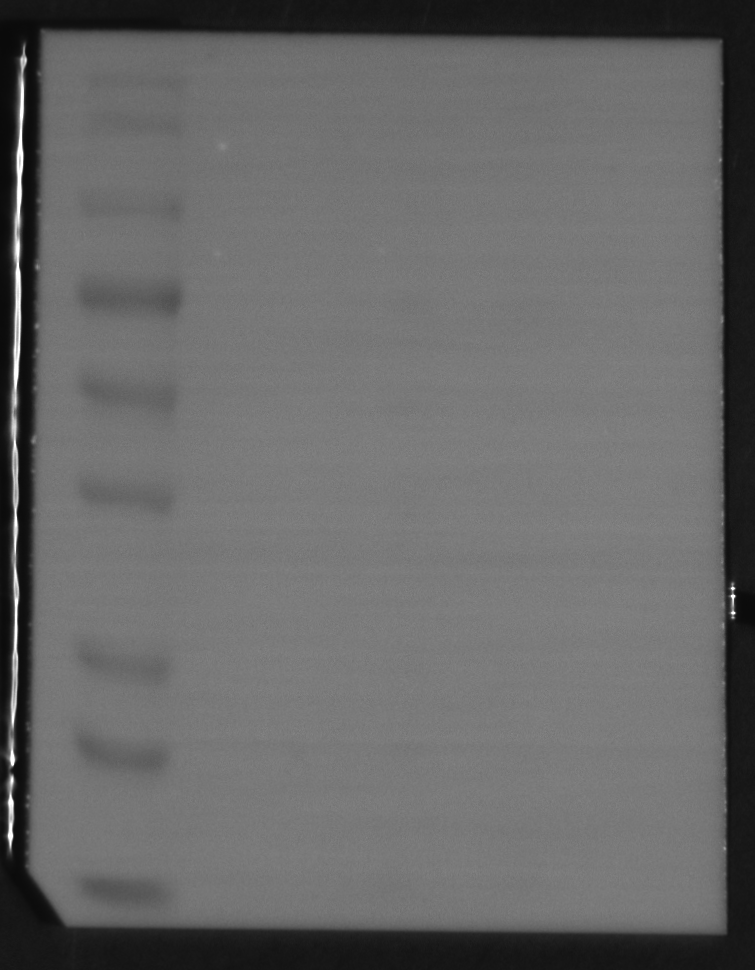

Supplement: Supplementary file 2 [file DataSheet1.zip › Original Western blot images collection/Experiment 5 White light image N-cadherin.tif]

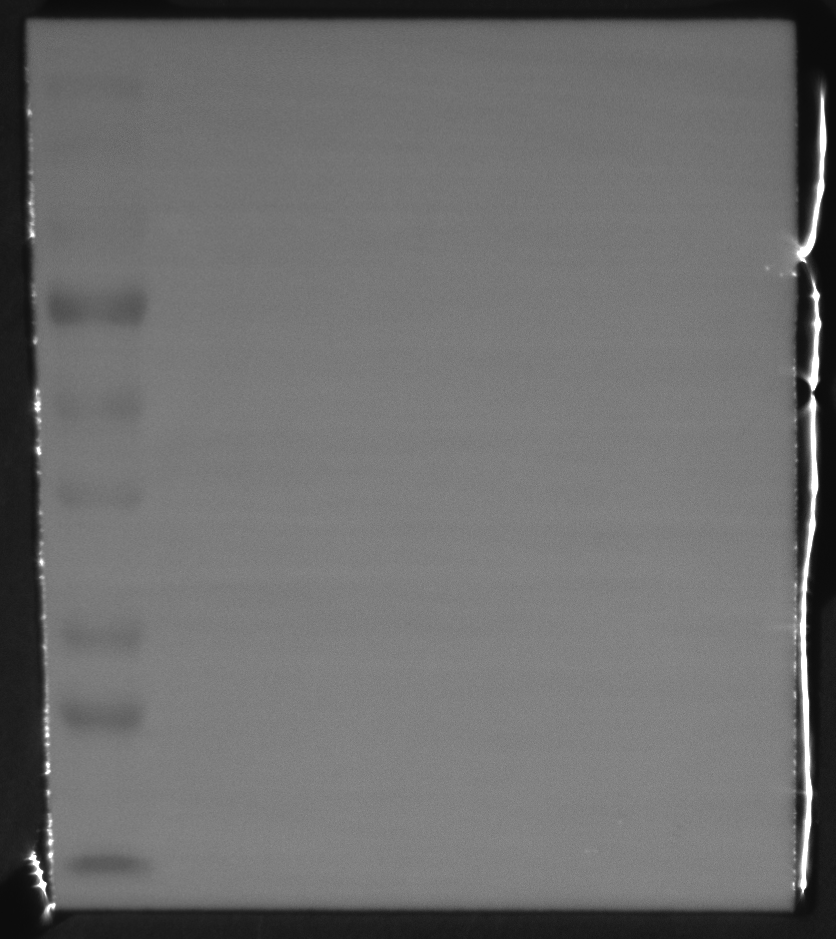

Supplement: Supplementary file 2 [file DataSheet1.zip › Original Western blot images collection/Experiment 5 White light image SREBP1.tif]

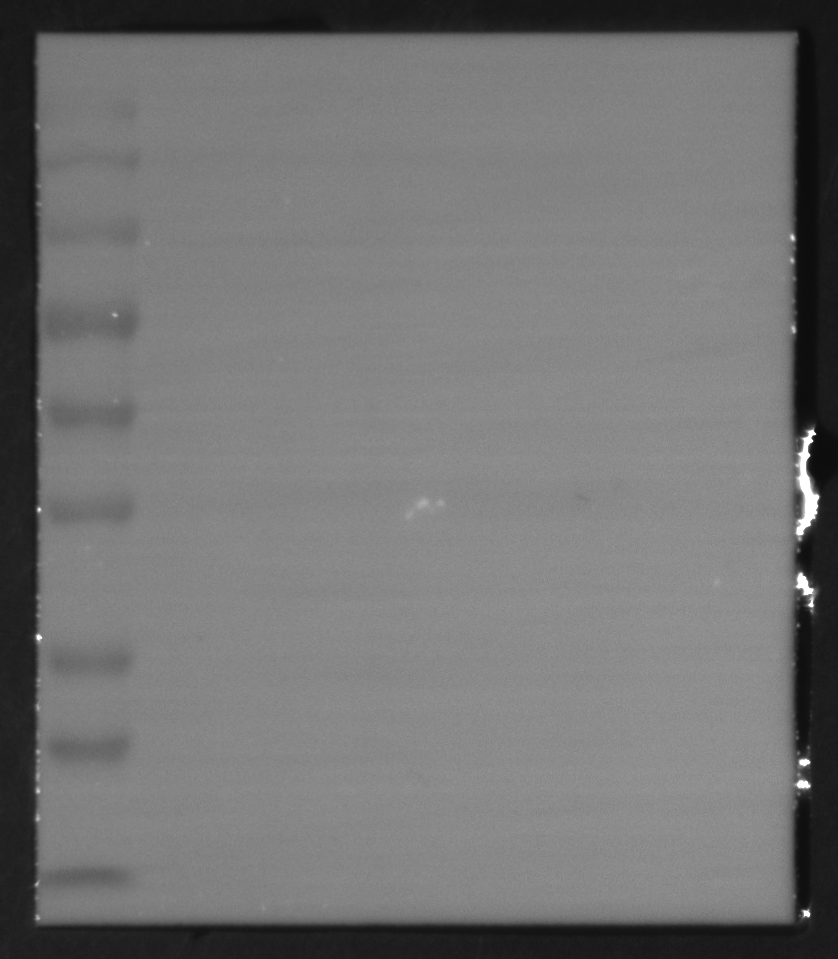

Supplement: Supplementary file 2 [file DataSheet1.zip › Original Western blot images collection/Experiment 5White light image Vimentin.tif]

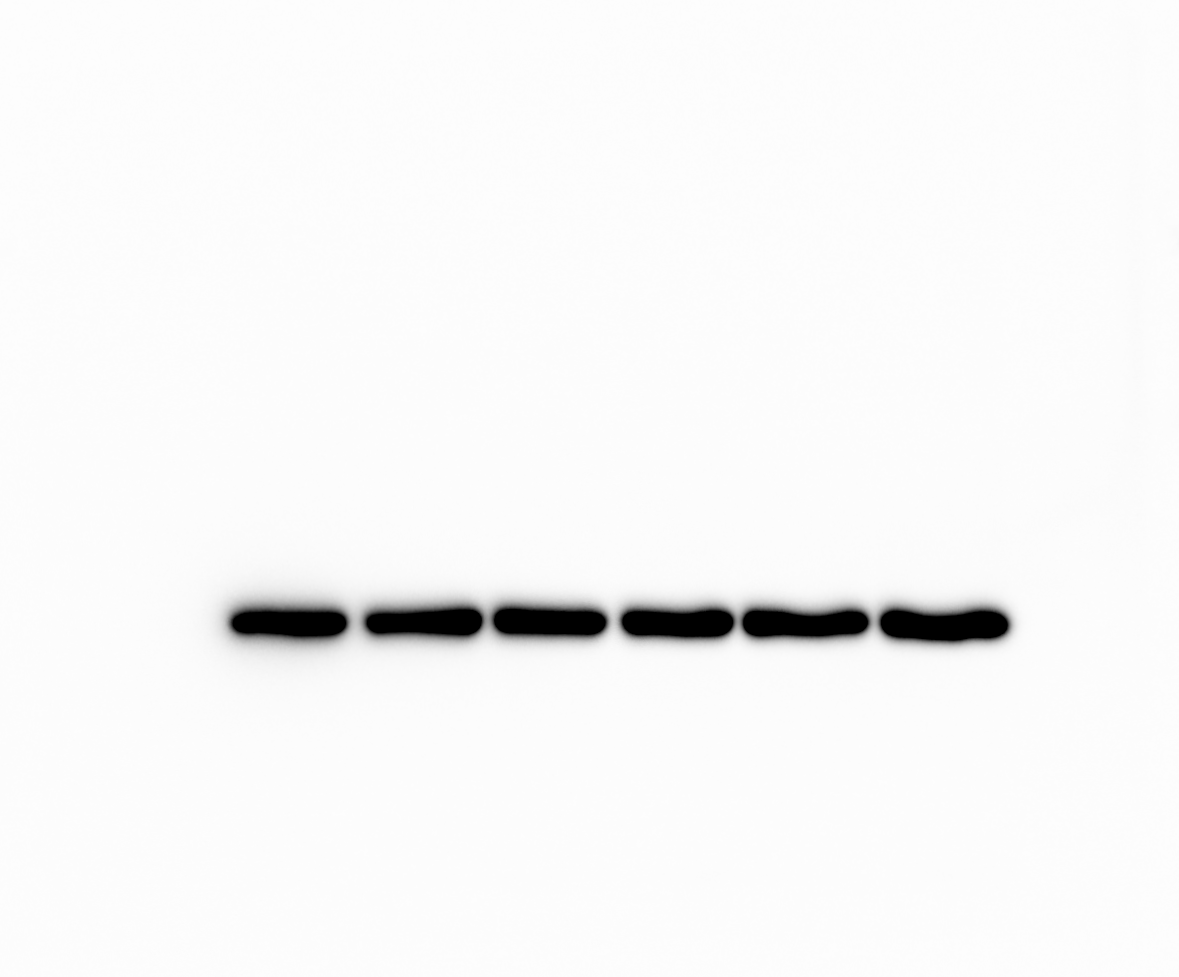

Supplement: Supplementary file 2 [file DataSheet1.zip › Original Western blot images collection/Experiment 6 Chemiluminescence image GAPDH.tif]

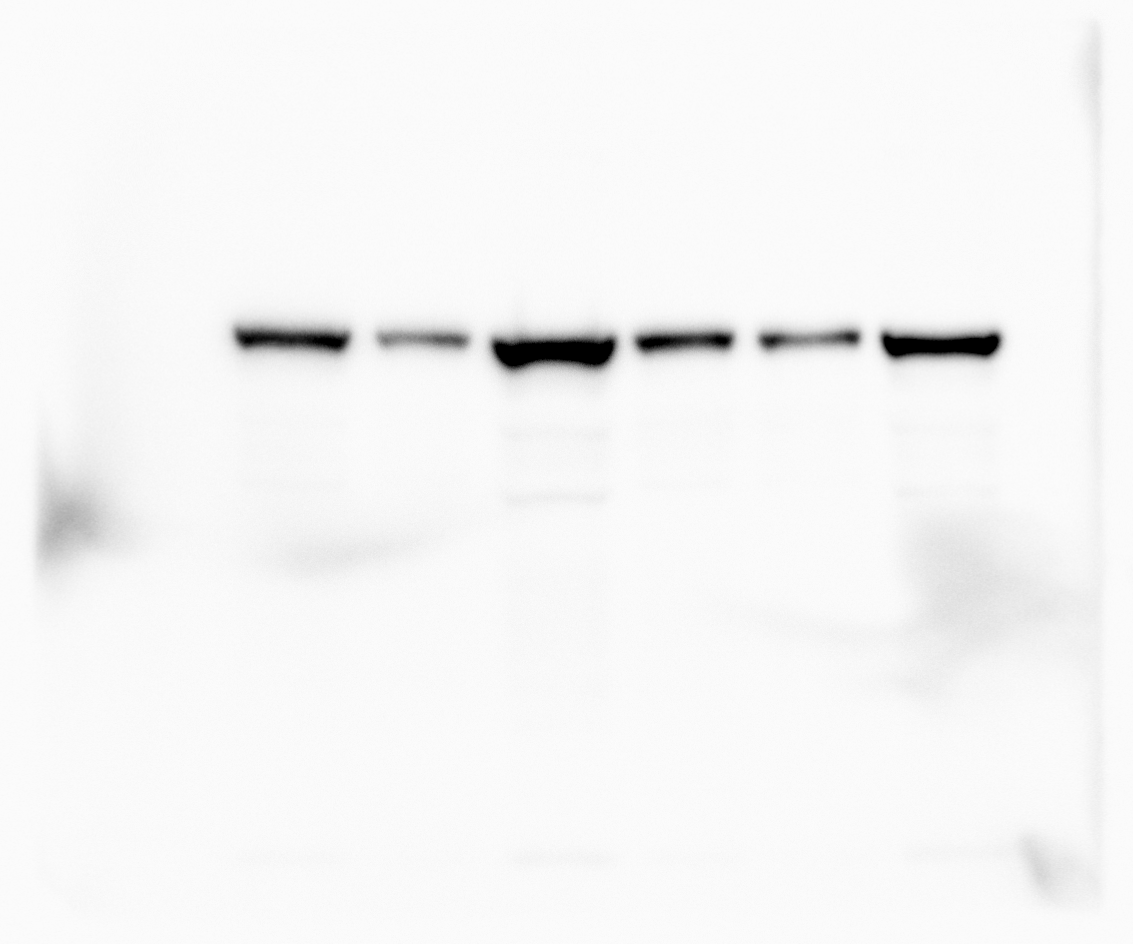

Supplement: Supplementary file 2 [file DataSheet1.zip › Original Western blot images collection/Experiment 6 Chemiluminescence image LPCAT1.tif]

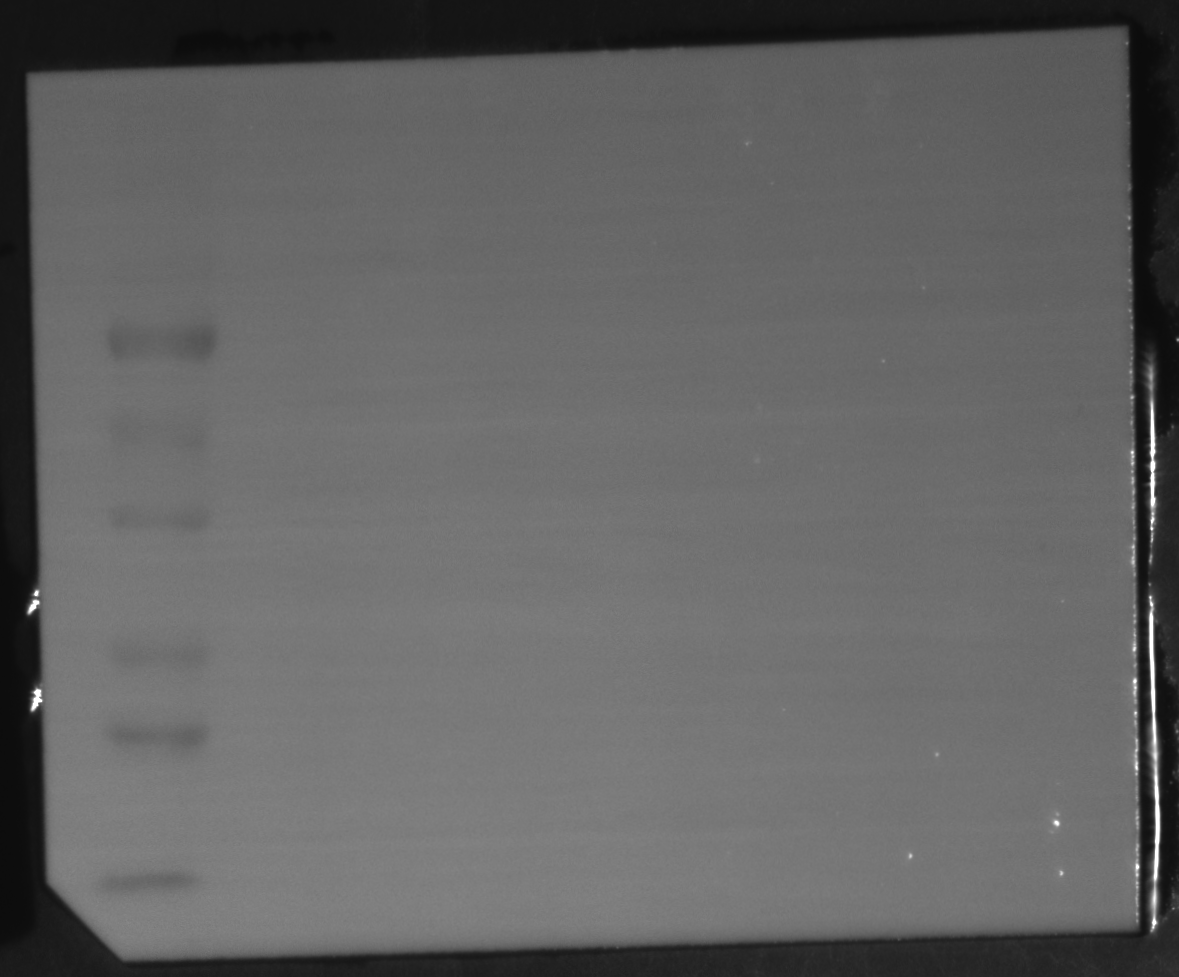

Supplement: Supplementary file 2 [file DataSheet1.zip › Original Western blot images collection/Experiment 6 White light image GAPDH.tif]

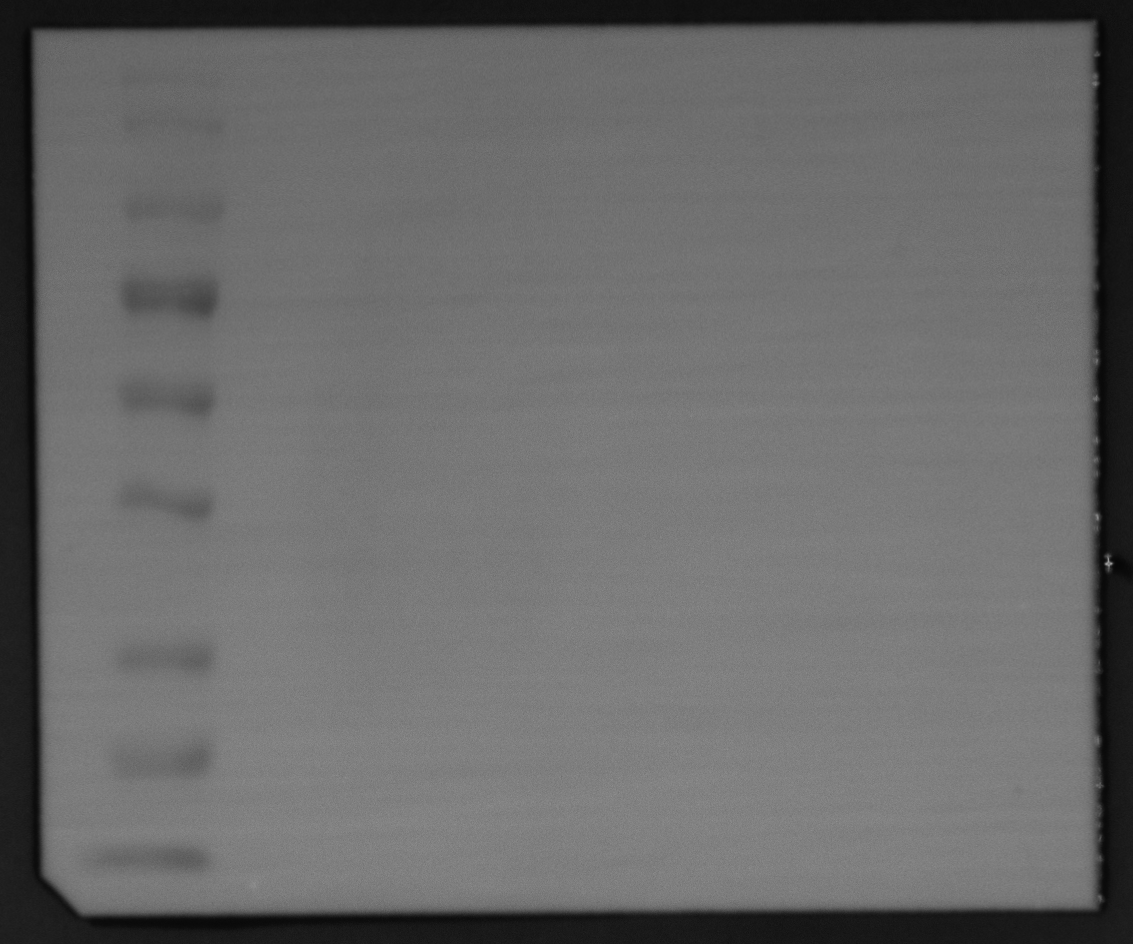

Supplement: Supplementary file 2 [file DataSheet1.zip › Original Western blot images collection/Experiment 6 White light image LPCAT1.tif]
